# A simplified modelling framework facilitates more complex representations of plant circadian clocks

**DOI:** 10.1371/journal.pcbi.1007671

**Published:** 2020-03-16

**Authors:** Mathias Foo, Declan G. Bates, Ozgur E. Akman

**Affiliations:** 1 School of Mechanical, Aerospace and Automotive Engineering, Coventry University, Coventry, United Kingdom; 2 Warwick Integrative Synthetic Biology Centre, School of Engineering, University of Warwick, Coventry, United Kingdom; 3 College of Engineering, Mathematics and Physical Sciences, University of Exeter, Exeter, United Kingdom; University of Connecticut School of Medicine, UNITED STATES

## Abstract

The circadian clock orchestrates biological processes so that they occur at specific times of the day, thereby facilitating adaptation to diurnal and seasonal environmental changes. In plants, mathematical modelling has been comprehensively integrated with experimental studies to gain a better mechanistic understanding of the complex genetic regulatory network comprising the clock. However, with an increasing number of circadian genes being discovered, there is a pressing need for methods facilitating the expansion of computational models to incorporate these newly-discovered components. Conventionally, plant clock models have comprised differential equation systems based on Michaelis-Menten kinetics. However, the difficulties associated with modifying interactions using this approach—and the concomitant problem of robustly identifying regulation types—has contributed to a complexity bottleneck, with quantitative fits to experimental data rapidly becoming computationally intractable for models possessing more than ≈50 parameters. Here, we address these issues by constructing the first plant clock models based on the S-System formalism originally developed by Savageau for analysing biochemical networks. We show that despite its relative simplicity, this approach yields clock models with comparable accuracy to the conventional Michaelis-Menten formalism. The S-System formulation also confers several key advantages in terms of model construction and expansion. In particular, it simplifies the inclusion of new interactions, whilst also facilitating the modification of regulation types, thereby making it well-suited to network inference. Furthermore, S-System models mitigate the issue of parameter identifiability. Finally, by applying linear systems theory to the models considered, we provide some justification for the increased use of aggregated protein equations in recent plant clock modelling, replacing the separate cytoplasmic/nuclear protein compartments that were characteristic of the earlier models. We conclude that as well as providing a simplified framework for model development, the S-System formalism also possesses significant potential as a robust modelling method for designing synthetic gene circuits.

## Author summary

Mathematical models have been widely employed as a complement to experimental work in elucidating the underlying mechanistic behaviour of the plant circadian clock. In this study, we investigate the use of a simplified modelling strategy, the S-System framework, to reduce the computational complexity of such models. We test the efficacy of our approach by constructing S-System versions of five established plant clock models, which we fit to synthetic and experimental gene expression data. We demonstrate that the S-System formulation can generate fits to expression data that are very similar to those of its more complex counterparts, yielding predictions with better accuracy in some cases. Our work reveals three principal benefits of the S-System formalism in modelling gene regulatory networks (GRNs) characterised by entrainable oscillations, typified by the circadian clock. Firstly, it enables new interactions to be incorporated in a very easy manner. Secondly, it facilitates the inference of unknown regulation types. Thirdly, it mitigates the parameter identifiability problem, thereby making it a suitable modelling method for designing GRN control circuits for synthetic biology applications. Taken together, our findings identify the S-System framework as a promising alternative approach for computational modelling of oscillatory GRN networks.

## Introduction

### Circadian clock networks

Most living organisms possess innate molecular clock machineries that govern their daily activity [1]. These machineries, known as circadian clocks, are responsible for the generation of endogenous oscillations in gene expression with a period close to 24 hours. Circadian oscillations enable the anticipation of diurnal environmental changes and the coordination of biological processes to occur at the optimal time of day. Some important biological functions that are circadian regulated include the mammalian sleep/wake cycle, fungal spore formation and plant leaf movement (see *e.g*. [2–4]). Moreover, interruption of the circadian rhythm can lead to a number of pathophysiological conditions, including poor metabolism, psychiatric disorders and immune system dysfunction (see *e.g*. [5–8]). At the molecular level, the underlying core mechanism of the circadian rhythm is generated via interlocking feedback loops between regulatory genes. This discovery was first made in the fruitfly and its significance is evident in the award of the 2017 Nobel Prize in Physiology or Medicine to the pioneers of molecular circadian systems research [9, 10].

### Modelling the plant circadian clock

The discovery of multiple plant circadian genes has revealed the complexity of the underlying gene regulatory network (GRN), driving the use of mathematical models to help unravel the mechanisms controlling circadian timekeeping, as evident through the active development of clock models in the higher plant *Arabidopsis thaliana*. Since the construction of the first *Arabidopsis* model in 2005 by Locke *et al*. [11], which comprised only two key clock genes, the models have expanded considerably in size and complexity, with frequent updates to incorporate more interactions as new experimental data became available. From a modeller’s perspective, updating a model to include new interactions is greatly facilitated if the model structure has low inherent complexity—*i.e*. an efficient parametrisation and minimal nonlinear terms, in the case of differential equation models. Overly complex models incur a high computational cost in terms of parameter optimisation [12, 13], thereby limiting their predictive capacity. They also tend to cloud the core behaviour of the system of interest, particularly the identification of possible network motifs. In view of this, many studies have focused their attention on reducing model complexity, including some notable examples that specifically addressed the plant clock.

### Reducing model complexity in plant clock models

In [14], instead of considering all circadian genes individually, the authors grouped several key circadian genes together and analysed the behaviour of the resulting reduced set of differential equations. In [15], the authors reduced the complexity of the model by identifying the *kernel* of the GRN—those genes that are solely accountable for generating circadian rhythms with behaviour similar to that of the wild-type.

In [12], the authors focused on reproducing key circadian characteristics (*e.g*. entrainment and photoperiodism) through the use of continuous-time Boolean models. The Boolean framework, in which genes are assumed to be either ‘OFF’ (0) if expression is below some threshold or ‘ON’ (1) if expression is above the threshold, yielded the following two key reductions in complexity: (i) all the parameters governing the time taken for the production of a transcription factor (TF) and its effect on a downstream gene (*e.g*. transcription rates, translation rates, degradation rates *etc*.) are telescoped into a single delay parameter; (ii) the complex nonlinear functions governing the expression of each gene are replaced by a constrained set of Boolean functions (logic gates), meaning that all the architectures consistent with a given circuit diagram can be systematically explored. In related work, distributed delays were used to represent TF production pathways in three established clock models. This approach replaces the set of parameters governing the delays in each pathway with a pair of parameters that control the mean and variance of the delay distribution (assumed to be a gamma function), leading to simplified differential equation models with markedly reduced parameterisations [16].

While those aforementioned approaches do dramatically reduce model complexity, they can come at the expense of either reducing the ability of the model to match quantitative behaviour (*e.g*. an inability to simulate amplitude modulation in the case of the Boolean models) or of favouring fits to specific biological phenotypes.

### S-Systems—A simplified framework for modelling the plant circadian system

The task of developing mathematical models of reduced complexity that still preserve accuracy presents a significant challenge to modellers. In the context of plant circadian systems biology, the network of identified clock genes is set to increase in size, given the extensive research in this area. A model structure is therefore required that facilitates modification and updating as further progress is made experimentally. The majority of plant clock models developed thus far have been sets of nonlinear differential equations based on Michaelis-Menten kinetics (see *e.g*. [11, 17–19]). However, modifying models based on this formalism to incorporate additional interactions (or revise existing ones) in the light of new experimental data can significantly increase computational complexity. This is predominately due to the inherent structure of Michaelis-Menten models, for which different regulation types (*i.e*. transcriptional activation and inhibition) are modelled with different nonlinear functions. The effect of this functional heterogeneity is further amplified when multiple transcriptional regulators are combined. Accordingly, if larger systems (*e.g*. of the order of 100 parameters or more) are to become amenable to quantitative modelling, alternative model formulations that mitigate this issue are required.

Here, we develop a minimal framework for modelling the plant circadian system using ordinary differential equations (ODEs), focusing mainly on simplifying the nonlinear functions governing gene expression. Our framework assumes a simple, homogenous model structure based on an extension of the S-System formalism originally developed by Savageau [20] to model biochemical systems, with specific modifications that enable the interactions between plant circadian genes and the photic environment to be represented. We note that although S-Systems have been used to model gene regulatory networks previously (see *e.g*. [21, 22]), circadian clocks have not been modelled with this formalism to date.

We investigate the capacity of our framework to quantitatively reproduce circadian dynamics by constructing modified S-System versions of a suite of established plant clock models. By fitting these S-System formulations to synthetic and experimental expression timeseries, and validating our fits in each case against hold-out data, we demonstrate that our formalism yields models with comparable predictive power to their Michaelis-Menten based counterparts. In addition, by employing a frequency response analysis technique from linear systems theory, we provide a mechanistic understanding of the progressive simplification of protein production pathways adopted by more recent models of the plant clock.

Finally, we highlight the advantages conferred by the S-System framework in modelling GRNs and synthetic GRN controllers, and also discuss possible further extensions to the framework to facilitate model construction and increase prediction accuracy.

## Materials and methods

### S-System modelling

The S-System modelling framework arose from biochemical system theory (see *e.g*. [20]), with the initial purpose of describing metabolic pathways. The original S-System model introduced in [20] can be written in the form

$$\frac{dX_{i}}{dt}=\alpha_{i}\prod_{j=1}^{n+m}X_{j}^{g_{i,j}}-\beta_{i}\prod_{j=1}^{n+m}X_{j}^{h_{i,j}},\;1\leq i\leq n,$$ (1)

where the dependent variables {*X*_1_, …, *X*_*n*_} represent the biochemical species of interest and the independent variables {*X*_*n*+1_, …, *X*_*n*+*m*_} represent forcing terms. For each *X*_*i*_, *α*_*i*_ represents the production rate constant, and the *g*_*i*,*j*_s are the exponents associated with production processes, whilst *β*_*i*_ denotes the degradation rate constant, and the *h*_*i*,*j*_s are the exponents associated with degradation processes.

Over the course of its development, the S-System framework has been used as an alternative approach for modelling a broad range of biological processes (see [23, 24] and references therein). These include signal transduction [25], metabolism [26] and enzyme kinetics [27–29]. In [30], the authors compared the validity of S-System and Michaelis-Menten models of enzyme-catalysed reactions in a rigorous manner, demonstrating that the two models had similar accuracy over the same concentration ranges. The accuracy of these two formulations has also been compared when modelling fully developed pathway systems—*e.g*. the fermentation pathway in yeast [31], purine metabolism in humans [32] and sphingolipid metabolism in yeast [33]—where both formulations have demonstrated comparable performance. In [34], the authors compared different kinetic models for the flowering time GRN in *Arabidopsis*, finding that S-System and Michaelis-Menten formulations possessed similar predictive capacity. The design principles predicted by the S-System approach for gene regulation in [35, 36] and protein modification in [37] have subsequently been verified in numerous experiments. The promising results of these studies prompted us to consider utilising S-Systems to model the plant clock.

### Extending the S-System modelling framework to circadian clocks

The generation of circadian rhythms in plants (and other organisms) is primarily governed by three mechanisms: (i) transcription—the process in which one or more TFs can bind to the specific promoter region of a gene to regulate the conversion of DNA into RNA; (ii) translation—the process in which protein is created by ribosomes in the cytoplasm following RNA transcription, prior to moving into the nucleus to control transcription; and (iii) protein modification—processes such as protein stabilisation and/or degradation that mediate the efficacy of a TF [38].

In order to model these mechanisms, as represented in the clock circuits of interest, some modifications to Eq (1) are required. Firstly, we introduce an extra term that represents the contribution from external light inputs. Secondly, to account for (i) complex TF regulation of gene expression, and (ii) protein modification, we introduce suitable summation operations to the first and second terms of Eq (1). With that, the modified S-System model can be written as below:

$$\frac{dX_{i}}{dt}=\alpha_{i}\prod_{j=1}^{n_{i}^{P}}{\left(\sum_{k=1}^{n}b_{i,j,k}X_{k}\right)}^{g_{i,j}}-\sum_{j=1}^{n_{i}^{D}}\beta_{i,j}X_{i}(\prod_{k=1}^{n}X_{k}^{h_{i,j,k}})+\sum_{j=1}^{n_{i}^{L}}\gamma_{i,j}U_{ij},\;1\leq i\leq n.$$ (2)

For a given *i*, *X*_*i*_ is the expression level of the *i*th clock gene/protein species, and each *U*_*ij*_ = *U*_*ij*_(*X*_1_, …, *X*_*n*_, *L*_*I*_(*t*)) represents the effect on *X*_*i*_ of a process regulated by the external light signal *L*_*I*_(*t*). Note that the explicit dependence of *U*_*ij*_ on {*X*_1_, …, *X*_*n*_} reflects the fact that our target models include the effect of light-regulated protein complexes on gene/protein expression, in addition to the effect of direct light regulation. For the clock models considered in this study, we assume that *U*_*ij*_ is a low-order polynomial function of its arguments. Furthermore, given the limited understanding and experimental evidence regarding the precise effect of light on many circadian genes and proteins (in terms of dimerisation *etc*.), no exponent is associated with *U*_*ij*_.

For all models considered here, *L*_*I*_(*t*) is assumed to be a periodic square wave with minimum and maximum values of 0 and 1 respectively, and *t* = 0 is taken to correspond to dawn, meaning that *L*_*I*_(*t*) is given by

$$L_{I}\left(t\right)=\{\begin{array}{cc} 1 & \text{if}\;0\leq t\;\text{mod}\;\text{24}<P;\\ 0 & \text{otherwise,} \end{array}$$ (3)

where *P* is the photoperiod. Accordingly, *P* = 0 and *P* = 24 correspond to constant dark (DD) and constant light (LL), respectively, while setting *P* = 12 simulates a symmetric light-dark cycle, *i.e*. alternating 12 hour periods of light and dark (12L:12D).

We further remark that in Eq (2), $n_{i}^{P}$, $n_{i}^{D}$ and $n_{i}^{L}$ denote the number of processes involved in the production, degradation and light regulation of *X*_*i*_, respectively. As in the original S-System formulation of Eq (1), *α*_*i*_ represents the production rate constant of *X*_*i*_, and the *g*_*i*,*j*_s are the exponents associated with production. The *b*_*i*,*j*,*k*_s are Boolean variables, *b*_*i*,*j*,*k*_ ∈ {0, 1}, which determine the species contributing to each particular production process. The degradation of *X*_*i*_ is determined by the rate constants of each contributing process $\left\{\beta_{i,j}:1\leq j\leq n_{i}^{D}\right\}$ together with the associated exponents $\left\{h_{i,j,k}:1\leq j\leq n_{i}^{D},1\leq k\leq n\right\}$. The *β*_*i*,*j*_s can take both positive and negative values, with the former representing degradation and the latter representing stabilisation. Finally, the *γ*_*i*,*j*_s determine the strength of the light-regulated processes affecting expression of *X*_*i*_. We note that the original S-System formulation can be recovered from Eq (2) by setting $n_{i}^{P}=n$, *b*_*i*,*j*,*k*_ = *δ*_*j*,*k*_, $n_{i}^{D}=n$, *β*_*i*,*j*_ = *β*_*i*_ *δ*_*i*,*j*_, *h*_*i*,*j*,*k*_ = *h*_*i*,*k*_ − *δ*_*i*,*k*_ and $n_{i}^{L}=0$, where *δ*_*i*,*j*_, *δ*_*j*,*k*_ and *δ*_*i*,*k*_ denote the Kronecker delta in each case. Moreover, the original S-System model (*cf*. Eq (1)) is itself a special case of the Generalised Mass Action (GMA) model [39, 40]. This has the general form

$$\frac{dX_{i}}{dt}=\sum_{k=1}^{T_{i}}\pm \gamma_{i,k}\prod_{j=1}^{n+m}X_{j}^{f_{i,j,k}},\;1\leq i\leq n,$$ (4)

where {*X*_1_, …, *X*_*n*_} and {*X*_*n*+1_, …, *X*_*n*+*m*_} again denote the dependent and independent variables, respectively. However, our extended S-System formulation (*cf*. Eq (2)) cannot, in general, be expressed in this form.

Having introduced our modified S-System modelling framework (hereafter termed the *extended S-System formalism*), we discuss in more detail below how it can be employed to describe the transcription, translation and protein modification mechanisms that are characteristic of our target clock models.

#### Extended S-System modelling of transcription

In general, there are two main types of transcriptional regulation: transcriptional activation, which increases gene transcription, and transcriptional inhibition, which decreases gene transcription. Conventionally, the mechanisms are modelled using a combination of Michaelis-Menten and Hill-type functions. For a gene *G* that is regulated by a single transcriptional regulator *P*, transcriptional activation is often modelled with an equation of the form

$$\frac{dG}{dt}=\frac{aP^{n}}{K^{n}+P^{n}}-bG,$$ (5)

while for transcriptional inhibition, the model structure is often given by

$$\frac{dG}{dt}=\frac{aK^{n}}{K^{n}+P^{n}}-bG.$$ (6)

In both equations, *K* is the Michaelis-Menten kinetic constant (threshold for activation/inhibition), *a* and *b* respectively parameterise the transcription and degradation rate constants and *n* represents the Hill coefficient (degree of binding cooperativity).

In the extended S-System formalism, both transcriptional activation and transcriptional inhibition, combined with the linear degradation used in Eqs (5) and (6), can be represented with the single model structure

$$\frac{dX_{i_{G}}}{dt}=\alpha_{i_{G}}X_{j_{P}}^{g_{i_{G},j_{P}}}-\beta_{i_{G}}X_{i_{G}},$$ (7)

where $X_{i_{G}}=G$ and $X_{j_{P}}=P$ (*i*_*G*_ ≠ *j*_*P*_). In this formulation, $g_{i_{G},j_{P}}>0$ models activation and $g_{i_{G},j_{P}}<0$ models inhibition. As in the original S-System formulation, $\alpha_{i_{G}}$ represents the production rate constant of $X_{i_{G}}$ (*i.e*. the transcription rate), $g_{i_{G},j_{P}}$ is the exponent associated with activation/inhibition, whilst $\beta_{i_{G}}$ denotes the degradation rate constant. It should be noted that this model of transcriptional regulation holds a key advantage over the standard Hill-type approach of Eqs (5) and (6) when it comes to network inference, *i.e*. when one is interested in identifying the type of regulation at each node of the GRN from the available experimental data: the regulation type can simply be inferred from the sign of the fitted value of $g_{i_{G},j_{P}}$, thereby avoiding the use of more complex nonlinear terms capable of smoothly interpolating between activation and inhibition [41].

We further note that when a gene is regulated by multiple TFs, modellers are required to select whether the TF interactions should be represented using the continuous analog of a multi-input OR logic gate (in which the terms modelling the effect of each TF on gene expression are summed together), the analog of a multi-input AND logic gate (in which the terms are multiplied together), or a combination thereof [12, 41, 42]. In plant clock modelling, inhibitors tend to be combined with other regulators using AND gates, whilst activators are combined using OR gates (*e.g*. [11, 43–45]). By default, the standard S-System model only implements the multi-input AND logic gate (we note that other logic gates can of course still be approximated with this approach, depending on the range of the input variables around the appropriate nominal operating point). Our extended S-System formulation, however, enables a broader set of multi-input logic gates to be natively represented, and in particular, the gates used in the plant clock models of interest.

#### Extended S-System modelling of translation

A standard approach to modelling translation in circadian clock models is to explicitly represent the shuttling of the translated protein between the cytoplasm (where translation occurs) and the nucleus (where the protein can regulate transcription) [38]. This shuttling mechanism, taken from [11, 43], is often described using the following pair of differential equations:

$$\begin{array}{cc} \frac{dP_{C}}{dt} & =aG-r_{C}P_{C}+r_{N}P_{N}-b_{C}P_{C},\\ \frac{dP_{N}}{dt} & =r_{C}P_{C}-r_{N}P_{N}-b_{N}P_{N}. \end{array}$$ (8)

In the above, *G* is the gene, whilst *P*_*C*_ and *P*_*N*_ denote cytoplasmic and nuclear protein, respectively. *a* is the translation rate, *r*_*N*_ and *r*_*C*_ are the shuttling rates, and *b*_*C*_ and *b*_*N*_ denote the degradation rate constant of each protein form. This model assumes that the translated protein is not subjected to further activities such as complex formation, protein stabilisation and/or protein degradation.

Protein shuttling can be represented in the extended S-System formalism as

$$\begin{array}{cc} \frac{dX_{i_{P_{C}}}}{dt} & =\alpha_{i_{P_{C}}}X_{j_{G}}^{g_{i_{P_{C}},j_{G}}}-\beta_{i_{P_{C}}}X_{i_{P_{C}}},\\ \frac{dX_{i_{P_{N}}}}{dt} & =\alpha_{i_{P_{N}}}X_{i_{P_{C}}}^{g_{i_{P_{N}},i_{P_{C}}}}-\beta_{i_{P_{N}}}X_{i_{P_{N}}}, \end{array}$$ (9)

where $X_{j_{G}}=G$, $X_{i_{P_{C}}}=P_{C}$ and $X_{i_{P_{N}}}=P_{N}$ (with all three indices $\left\{j_{G},i_{P_{C}},i_{P_{N}}\right\}$ distinct). In Eq (9), $\alpha_{i_{P_{C}}}$ represents the production rate constant of $X_{i_{P_{C}}}$ (*i.e*. the translation rate) and $\left\{\beta_{i_{P_{C}}},\beta_{i_{P_{N}}}\right\}$ are the protein degradation rate constants. For fixed values of the latter, the rate of protein shuttling between cytoplasm and nucleus is determined by the production rate constant $\alpha_{i_{P_{N}}}$ of $X_{i_{P_{N}}}$, together with the exponents $g_{i_{P_{C}},j_{G}}$ and $g_{i_{P_{N}},i_{P_{C}}}$.

Many of the earlier plant clock models (*e.g*. [11, 43]) used protein shuttling to promote oscillatory behaviour [38]. However, due to the limited knowledge available regarding time-dependent expression patterns of cytoplasmic and nuclear proteins in plant clock GRNs, it has now become common practice to consider the aggregated effect of these two proteins instead, representing them with a single ODE of the form below:

$$\frac{dP}{dt}=aG-bP.$$ (10)

In this simple model, *P* and *G* represent protein and gene respectively, whilst *a* is the translation rate and *b* is the degradation rate constant. Although this is already in our extended S-System form, its most general representation is

$$\frac{dX_{i_{P}}}{dt}=\alpha_{i_{P}}X_{j_{G}}^{g_{i_{P},j_{G}}}-\beta_{i_{P}}X_{i_{P}},$$ (11)

where $X_{i_{P}}=P$, $X_{j_{G}}=G$ (*i*_*P*_ ≠ *j*_*G*_), and $\alpha_{i_{P}}$ and $\beta_{i_{P}}$ are the translation and degradation rate constants, respectively. The exponent $g_{i_{P},j_{G}}$ in Eq (11) can model both linear and nonlinear dependence of protein production on mRNA dependence (*e.g*. setting $g_{i_{P},j_{G}}=1$ recovers Eq (10)).

The aggregated protein model of Eq (10) has been used in more recent plant clock models (*e.g*. see [44, 45]). Moreover, as we show later, linear systems theory (frequency response analysis) can provide some insights into why a single ODE can be sufficient to describe protein translation in the models of interest.

#### Extended S-System modelling of protein modification

After translation, proteins may undergo protein complex formation, protein stabilisation and/or protein degradation, amongst other processes (*i.e*. post-translational protein modification). Following [15, 44, 45], the formation of a protein complex *C* composed of *N* proteins {*P*_1_, *P*_2_, … *P*_*N*_} can be modelled by the equation

$$\frac{dC}{dt}=aP_{1}P_{2}\ldots P_{N}-bC,$$ (12)

where *a* and *b* denote the rates of protein-protein association and complex degradation, respectively.

On the other hand, if we assume for example, that protein *P* is translated from gene *G*, stabilised by *P*_*S*_ and degraded by *P*_*D*_, then following [15, 44, 45], these protein-mediated stabilisation and degradation processes can be modelled by the equation

$$\frac{dP}{dt}=aG-bP+c_{S}PP_{S}-c_{D}PP_{D},$$ (13)

where parameters *a*, *b*, *c*_*S*_ and *c*_*D*_ represent the rates of translation, degradation, stabilisation and protein-mediated degradation, respectively.

Note that Eq (12) is already in the extended S-System form with the exponents *g*_*i*,*j*_ and *h*_*i*,*j*,*k*_ set to unity. Likewise, it can also be clearly seen that Eq (13) can be cast in the form of Eq (2) through appropriate choices of coefficients and exponents.

### Extended S-System formulations of existing plant clock models

Using the framework outlined above, we constructed the extended S-System versions of four well-established plant clock models of varying complexity—JL2005 [11], JL2006 [43], AP2012 [44] and KF2014 [45], where we have used the initials of the leading author’s first and last names followed by the year of publication to name the models. Each of these models employed the conventional Michaelis-Menten based modelling approach. Here, we append the notation ‘S’ to each plant clock model to denote its S-System variant (*e.g*. JL2005S denotes the extended S-System formulation of JL2005). In order to assess the degree to which these variants could reproduce the dynamics of the standard ODE representations, each extended S-System formulation was fitted to a synthetic dataset generated by the original model (*training data*) [12]. The out-of-sample error was then evaluated by scoring the extended S-System model against a second, distinct synthetic dataset also generated by the original model (*validation data*).

In addition, to further probe the predictive capacity of our formalism, we developed an extended S-System variant, MF2016KS, of a more recent clock model, MF2016K [15], which was fitted to an experimental training dataset. We also constructed a version of this model, MF2016KSorig, using the standard S-System formulation of Eq (1), and fitted it to the same dataset in order to assess the extent to which the extended formalism improved data-fitting. Mirroring the method used for synthetic data, the out-of-sample error for each model was evaluated with an experimental validation dataset.

The original ODE formulations for each model are given as eqs. (S1.1) (JL2005), eqs. (S1.3) (JL2006), eqs. (S1.5) (AP2012), eqs. (S1.7) (KF2014) and eqs. (S1.9) (MF2016K) of section 1 in S1 Text.

### Fitting to synthetic data

For each model, we first generated timeseries for all circadian genes and proteins under transition from a 12L:12D light-dark cycle (*i.e*. alternating intervals of 12 hours of light and 12 hours of dark) to a constant light regime (LL). The parameters of the corresponding extended S-System formulation were then fitted to this synthetic training set by minimising the weighted mean squared error (WMSE) between the simulated and generated timeseries, *i.e*. by finding

$${\hat{\Theta }}_{LL}^{S}=\underset{\Theta }{\text{argmin}}\;W(X_{LL},{\hat{X}}_{LL}\left(\Theta \right)),$$ (14)

where

$$W(X_{LL},{\hat{X}}_{LL}\left(\Theta \right))=\frac{1}{N_{G}}\sum_{i=1}^{N_{G}}W_{i}(X_{i}^{LL},{\hat{X}}_{i}^{LL}\left(\Theta \right))$$ (15)

and

$$W_{i}(X_{i}^{LL},{\hat{X}}_{i}^{LL}\left(\Theta \right))=\frac{1}{N_{T}}\sum_{j=1}^{N_{T}}{\left(\frac{X_{i}^{LL}\left(t_{j}\right)-{\hat{X}}_{i}^{LL}\left(t_{j},\Theta \right)}{A_{i}}\right)}^{2}$$ (16)

with

$$A_{i}=\underset{1\leq j\leq N_{T}}{\text{max}}X_{i}^{LL}\left(t_{j}\right).$$ (17)

The total WMSE, *W*, is the sum of the individual WMSEs, *W*_*i*_, computed for each of the *N*_*G*_ circadian components in the plant clock model, for a given parameter set Θ. As different genes/proteins have different amplitudes, the weights *A*_*i*_ in the expression for *W*_*i*_ normalise each timeseries to its maximum value, in order to mitigate bias in the optimisation procedure when fitting the model parameters. In Eqs (15) and (16),

$$X_{LL}\left(t\right)=(X_{1}^{LL}\left(t\right),\ldots ,X_{N_{G}}^{LL}\left(t\right))$$ (18)

represent the timeseries generated from the original plant clock model in the simulated 12L:12D→LL transition, whilst

$${\hat{X}}_{LL}\left(t,\Theta \right)=({\hat{X}}_{1}^{LL}\left(t,\Theta \right),\ldots ,{\hat{X}}_{N_{G}}^{LL}\left(t,\Theta \right))$$ (19)

are the timeseries generated by the extended S-System variant in the same simulated transition for parameters Θ and *N*_*T*_ is the number of timeseries points used to score each circadian component. The minimisation was carried out using the MATLAB function fminsearch, which implements the Nelder-Mead simplex algorithm [46].

Next, for each model, we compared the dynamics of the original model and its extended S-System formulation under a different light condition—the transition from a 12L:12D light-dark cycle to constant dark (DD). To quantitatively assess the performance of the model on this validation set, the total WMSE was calculated using Eqs (15) and (16) as

$$W(X_{DD},{\hat{X}}_{DD}({\hat{\Theta }}_{LL}^{S})),$$ (20)

where

$$X_{DD}\left(t\right)=(X_{1}^{DD}\left(t\right),\ldots ,X_{N_{G}}^{DD}\left(t\right))$$ (21)

denotes the timeseries generated from the original model in the simulated 12L:12D→DD transition, and

$${\hat{X}}_{DD}\left(t,\Theta \right)=({\hat{X}}_{1}^{DD}\left(t,\Theta \right),\ldots ,{\hat{X}}_{N_{G}}^{DD}\left(t,\Theta \right))$$ (22)

is the corresponding timeseries generated by the extended S-System formulation for parameters Θ.

The parameter sets used to generate synthetic data for each model are listed in S2 Table (JL2005), S5 Table (JL2006), S8 Table (AP2012) and S11 Table (KF2014). We refer to these parameter sets as the nominal parameter values in each case. The MATLAB files used to generate synthetic data from each of these clock models can be downloaded at https://github.com/mathiasfoo/essystemplantcircadian.

### Fitting to experimental data

In [15], two models of the plant circadian clock were developed—the full model, labelled MF2016, and the reduced kernel model, labelled MF2016K. The kernel model describes the core genetic circuitry that is responsible for generating wild-type behaviour of the plant circadian clock. Both the original (MF2016KSorig) and extended S-System (MF2016KS) formulations of this model were fitted to experimental data recorded in a 12L:12D→LL transition by finding the parameter set

$${\hat{\Theta }}_{LL}^{E}=\underset{\Theta }{\text{argmin}}\;W(D_{LL},{\hat{X}}_{LL}\left(\Theta \right)),$$ (23)

calculated using Eqs (15) and (16), where

$$D_{LL}\left(t\right)=(D_{1}^{LL}\left(t\right),\ldots ,D_{N_{G}}^{LL}\left(t\right))$$ (24)

denotes the experimental timeseries and

$${\hat{X}}_{LL}\left(t,\Theta \right)=({\hat{X}}_{1}^{LL}\left(t,\Theta \right),\ldots ,{\hat{X}}_{N_{G}}^{LL}\left(t,\Theta \right))$$ (25)

represent the timeseries generated by MF2016KS for parameters Θ in a simulated 12L:12D→LL transition. Like in the case of synthetic data, minimisation was carried out using fminsearch. We note that in fitting MF2016KSorig to experimental data, we made a minor amendment to the light forcing term in Eq (3), setting the minimum value of *L*_*I*_(*t*) to 0.001 instead of 0. This was because in the original S-System formulation, production terms involving the expression $L_{I}{\left(t\right)}^{g_{i,j}}$ become undefined if *L*_*I*_(*t*) = 0 and *g*_*i*,*j*_ < 0 (see Eq (1) above and eqs. (S1.11) in S1 Text).

Replicating the approach used for synthetic data-fitting, the validation goodness-of-fit was then calculated using experimental timeseries recorded in a 12L:12D→DD transition as

$$W(D_{DD},{\hat{X}}_{DD}({\hat{\Theta }}_{LL}^{E})),$$ (26)

where

$$D_{DD}\left(t\right)=(D_{1}^{DD}\left(t\right),\ldots ,D_{N_{G}}^{DD}\left(t\right))$$ (27)

denotes the experimental timeseries and

$${\hat{X}}_{DD}\left(t,\Theta \right)=({\hat{X}}_{1}^{DD}\left(t,\Theta \right),\ldots ,{\hat{X}}_{N_{G}}^{DD}\left(t,\Theta \right))$$ (28)

is the corresponding simulation of MF2016KS or MF2016KSorig for parameters Θ.

The experimental data used for training and validation is presented as DataSet S1 and Table S1 of the Supporting Information in [15]. Note that not all experimental data was available in the literature for all circadian genes in all light conditions (for example, there was no data available for LHY protein under LL and DD conditions—see [15, 45]). To address this issue, the authors in [15] had used a data processing approach that combined synthetic and experimental data to produce timeseries for unavailable components. In this work, we use the same processed data from [15] to implement the parameter optimisation procedure described above. We also compare the fits to this data obtained with the two S-System models of the kernel structure, MF2016KS and MF2016KSorig, with the fit obtained previously in [15] using the Michaelis-Menten model formulation, MF2016K. The MATLAB files used for implementing MF2016K, MF2016KS and MF2016KSorig can be downloaded at https://github.com/mathiasfoo/essystemplantcircadian.

#### Assessing relative quality of fit using the AIC

In order to quantify the relative quality of the fits to the experimental training data obtained with MF2016KS, MF2016KSorig and MF2016K, we employed the widely-used Aikake Information Criterion (AIC), which calculates the best approximating model to a given dataset with respect to Kullback-Leibler information loss [47, 48]. For a given model, the AIC is defined as

$$\text{AIC}=-2\;\text{ln}(\hat{L})+2K,$$ (29)

where $\text{ln}(\hat{L})$ is the maximised log-likelihood and *K* is the total number of estimated parameters. For the models considered here, since optimal parameter estimates were obtained by minimising a weighted least squares cost function, it can be shown that

$$\text{ln}\left(\hat{L}\right)=-\frac{N_{G}N_{T}}{2}\text{ln}\left(2\pi +1\right)-N_{T}\sum_{i=1}^{N_{G}}\text{ln}\left(A_{i}\right)-\frac{N_{G}N_{T}}{2}\text{ln}(W(D_{LL},{\hat{X}}_{LL}\left({\hat{\Theta }}_{LL}^{E}\right))),$$ (30)

where the *A*_*i*_s (defined in Eq (17)) are the cost function weights and ${\hat{\Theta }}_{LL}^{E}$ (defined in Eq (23)) is the parameter set that minimises the cost function [49].

Writing AIC_*i*_ for the AIC value of the *i*th model, we rank the three models of interest by calculating the AIC differences

$$\Delta_{i}\left(\text{AIC}\right)={\text{AIC}}_{i}-\underset{1\leq i\leq 3}{\text{min}}{\text{AIC}}_{i},$$ (31)

and the corresponding Aikake weights, defined below:

$$w_{i}\left(\text{AIC}\right)=\frac{\text{exp}(-\frac{1}{2}\Delta_{i}\left(\text{AIC}\right))}{\sum_{i=1}^{3}\text{exp}(-\frac{1}{2}\Delta_{i}\left(\text{AIC}\right))}.$$ (32)

Weight *w*_*i*_(AIC) can be interpreted as the probability that the *i*th model is the best (in the sense of minimising K-L information loss), given the data and set of candidate models. Furthermore, the strength of evidence in favour of model *i* over model *j* is quantified by the ratio *w*_*i*_(AIC)/*w*_*j*_(AIC) [47–50].

Finally, since *N*_*G*_, *N*_*T*_ and *A*_*i*_ in Eq (30) are fixed across the models, it follows that the AIC differences and Aikake weights can be computed from the following simplified expression for the AIC value of a given model

$$\text{AIC}=N_{G}N_{T}\;\text{ln}(W(D_{LL},{\hat{X}}_{LL}\left({\hat{\Theta }}_{LL}^{E}\right)))+2\left(K_{\Theta }+1\right).$$ (33)

In the above, *K*_Θ_ denotes the number of model parameters that are optimised using the weighted least squares cost function to calculate ${\hat{\Theta }}_{LL}^{E}$.

### Variability in optimised parameter values

In order to quantify the parameter variability associated with our optimisation procedure when fitting extended S-System models to synthetic/experimental data, we performed the following analysis. For each model, following an initial parameter optimisation run, five further runs were executed in which the initial parameters were sampled from a multivariate normal distribution with its mean set to the optimal values of the initial run and its covariance matrix set to the identity matrix. Parameter sampling was carried out using the MATLAB function mvnrnd. During the search process, if no further improvement was observed in the cost function (*i.e*. the WMSE value of the fit), this was taken to indicate the presence of a local minimum and the optimisation run was terminated. For each model, six parameter sets that could reproduce the synthetic/experimental data were thus generated in this manner. To obtain a robust measure of the variability of a given parameter *θ*, we then computed its normalised Median Absolute Deviation (nMAD), using the following equation:

$$\text{nMAD(}\theta \text{)}=\frac{\text{median}(|\theta_{i}-\tilde{\theta }|)}{|\tilde{\theta }|}.$$ (34)

In the above, *θ*_*i*_ is the optimal value of *θ* obtained for the *i*th run and $\tilde{\theta }=\text{median}\left(\theta_{i}\right)$.

## Results

### Extended S-System representations of the models

For each of the five plant clock models considered in this study, our extended S-System formulations of the corresponding GRN circuits are presented as eqs. (S1.2) (JL2005S), eqs. (S1.4) (JL2006S), eqs. (S1.6) (AP2012S), eqs. (S1.8) (KF2014S) and eqs. (S1.10) (MF2016KS) of section 1 in S1 Text.

### Fits to synthetic data

The parameter values yielding the best fits of each model to the corresponding training dataset are given in S3 Table (JL2005S), S6 Table (JL2006S), S9 Table (AP2012S) and S12 Table (KF2014S). Below, we discuss the performance of each optimised model in turn.

#### JL2005

**Fig 1:**
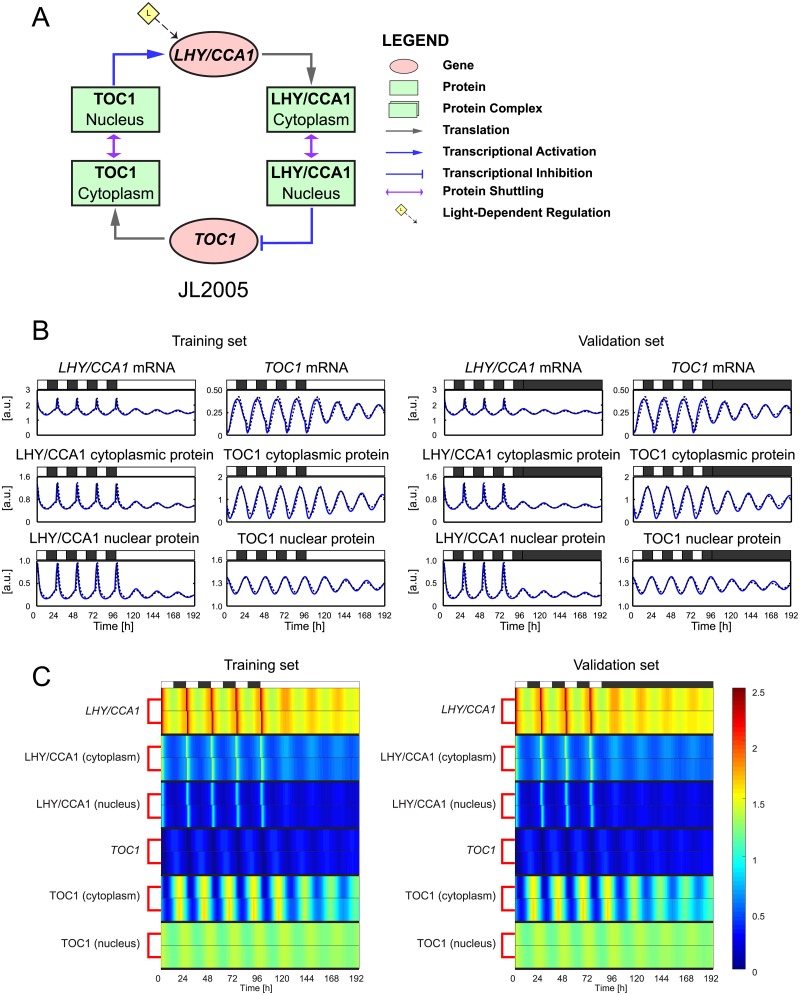
The JL2005S plant clock model—Fits to synthetic data. **A**: Regulatory circuit diagram for JL2005 [11]. Genes and proteins are represented as ovals and rectangles respectively. Grey solid lines represent translation. Blue solid lines with arrow heads (resp. bar heads) represent transcriptional activation (resp. inhibition). Double headed arrows denote protein shuttling between the cytoplasm and nucleus. The yellow diamond denotes light-dependent regulation. **B**: Comparison between expression timeseries in JL2005 (blue solid lines) and its extended S-System formulation JL2005S (black dashed lines). JL2005S was optimised to training data generated from JL2005 in a simulated 12L:12D → LL transition (left panels). For validation, the behaviour of the models was then evaluated in a simulated 12L:12D → DD transition (right panels). White and black bars represent intervals of light and dark. **C**: Heatmap representation of the timeseries shown in B. Each gene/protein is grouped together, with the JL2005 timeseries plotted in the top row of each group and the JL2005S timeseries plotted in the bottom row.

The simplest plant clock model we consider here is JL2005 (Fig 1A), which only comprises two circadian genes, *LHY/CCA1* and *TOC1*. In [11], this circuit is described with seven ODEs, in which one equation is used exclusively to represent the interaction of light with a light-sensitive protein, and is therefore decoupled from the others. Since this light-sensitive protein is not part of the core plant clock, we omit it from the timeseries and heatmap plots used to present the fitting results below.

The fits of JL2005S to the training and validation sets are shown in Fig 1B and 1C. In Fig 1B, we plot the timeseries, while in Fig 1C, we plot the corresponding heatmaps. For the heatmaps, each gene or protein is represented by two rows, where the top and bottom rows represent the expression dynamics generated by JL2005 and JL2005S respectively. The timeseries and heatmaps demonstrate excellent agreement between the two models, as quantified by the small WMSE values given in S4 Table. Both models attain peaks and troughs at the same times with near-identical amplitudes, demonstrating the capacity of the extended S-System formulation to reproduce the dynamics of the original equations with a simpler model structure. We also note that the model parameters are quite tightly constrained (*i.e*. have low variability across optimisation runs), with all parameters having nMAD values less than 0.25 (see S12A Fig and S3 Table).

#### JL2006

**Fig 2:**
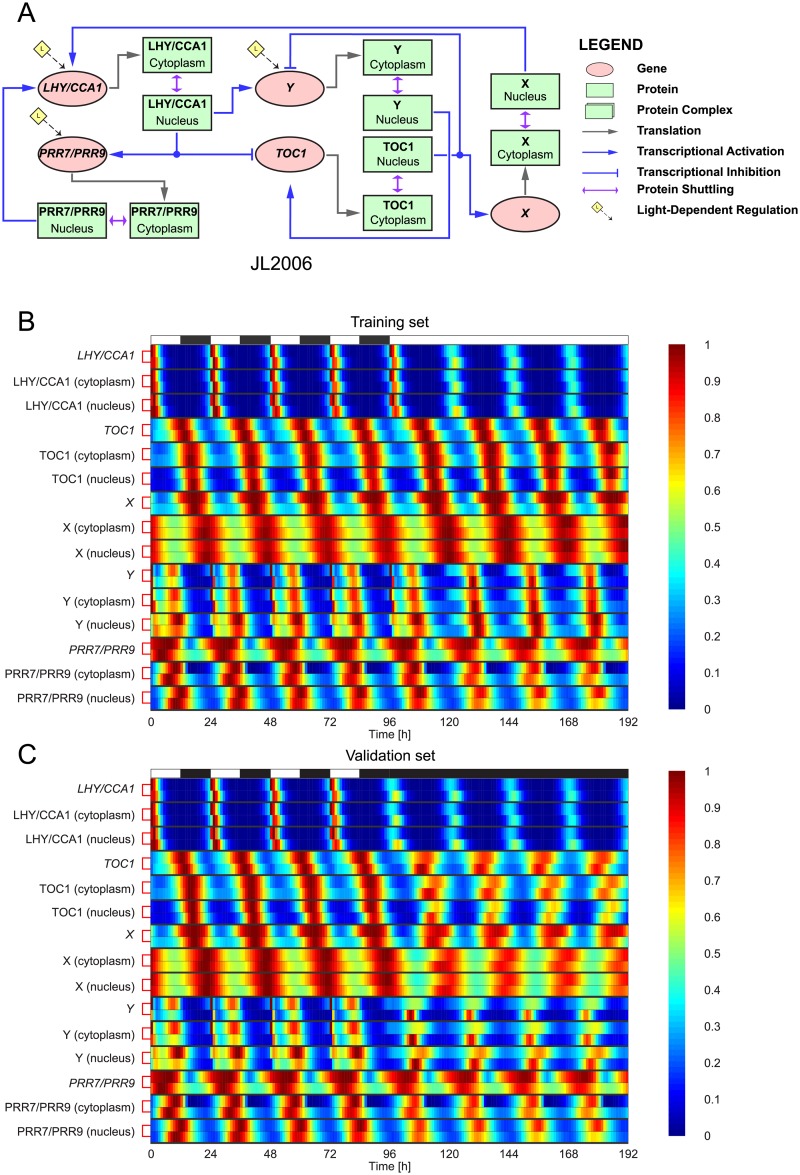
The JL2006S plant clock model—Fits to synthetic data. **A**: Regulatory circuit diagram for JL2006 [43]. The same symbols were used as in Fig 1. **B-C**: Comparison between expression timeseries in JL2006 and its extended S-System formulation JL2006S. JL2006S was optimised to training data generated from JL2006 in a simulated 12L:12D → LL transition (B). For validation, the behaviour of the models was then evaluated in a simulated 12L:12D → DD transition (C). Timeseries are presented as heatmaps in which each gene/protein is grouped together, with the top and bottom rows in each group showing JL2006 and JL2006S expression levels, respectively. To aid visualisation, each timeseries has been normalised to its maximum value. White and black bars represent intervals of light and dark.

The second plant clock model, JL2006 was constructed by expanding JL2005 from a two-gene to a five-gene circuit [43] (see Fig 2A). Note that in JL2006, there are two *speculative* genes labelled *X* and *Y*, reflecting the fact that when modifying JL2005 to better fit experimental data, Locke *et al*. proposed that there should be a gene (*X*) that acts as an intermediate genetic component between *TOC1* and *LHY/CCA1*, and another gene (*Y*) that acts as an intermediate genetic component between *LHY/CCA1* and *TOC1*. The identity of these two genes was eventually discovered and they will be discussed when we consider AP2012 below. JL2006 comprises 16 ODEs. As with JL2005, we omit the light-sensitive protein from the presentation of the fitting results, as it is not part of the core circadian clock.

Fig 2B and 2C show the fits of JL2006S to synthetic data in the form of heatmaps (the corresponding WMSE values are given in S7 Table). Because the expression amplitude of each gene and protein differs significantly, we normalised each timeseries by its maximum value when plotting the heatmaps, yielding a maximum relative amplitude of one (the unnormalised timeseries are shown in S1 and S2 Figs). For the training dataset, excellent agreement was observed between the two models, with both formulations attaining peaks and troughs at very similar times with similar amplitudes. Furthermore, JL2006S reproduces the acute light responses of the *Y* gene and Y protein that occur at dawn in light-dark cycles. For the validation dataset, both models also show good agreement, with the exception of *Y* mRNA and Y protein in DD conditions, where higher amplitudes are observed for JL2006S.

In terms of parameter variability, it can be seen in S12B Fig and S6 Table that similarly to JL2005S, the parameters of JL2006S are fairly well constrained, with the majority (56/61 parameters) having nMAD values less than 0.5, and all parameters having nMAD values less than 0.8.

#### AP2012

Although the extended S-System approach was very successful in reproducing the circadian dynamics of JL2005 and JL2006, these two plant clock models are characterised by simple transcription and translation mechanisms, where post-translational processes such as protein complex formation, protein stabilisation and protein degradation are not considered. The capacity of the extended S-System approach to describe these more complicated protein modification steps was assessed by applying it to the third plant clock model, AP2012 [44].

In AP2012, the previously unknown gene *Y* in JL2006 had been identified as *GI*. The other unknown component of JL2006, gene *X*, had been removed on the basis of new experimental work indicating that TOC1 protein was a transcriptional *inhibitor* of *LHY/CCA1* [44, 51, 52], rather than a transcriptional *activator* as had been initially assumed (this change in TOC1 function was also predicted by Boolean modelling in [12]). Modifying the regulation of *LHY/CCA1* by TOC1 in accordance with these findings resulted in the new model being able to fit a broader range of experimental data, leading to the removal of gene *X* from the model (and its subsequent extensions).

**Fig 3:**
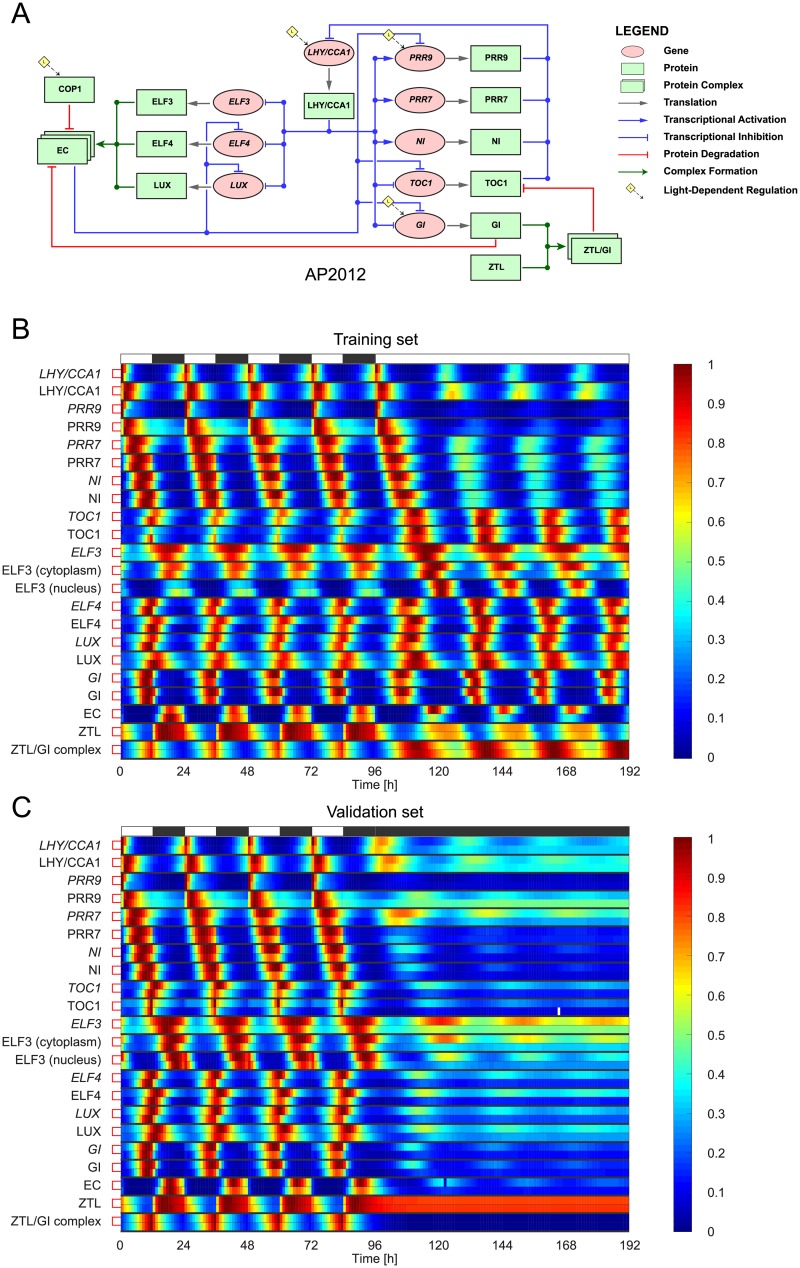
The AP2012S plant clock model—Fits to synthetic data. **A**: Regulatory circuit diagram for AP2012 [44]. The same symbols were used as in Fig 1, with the addition of green solid lines representing complex formation. **B-C**: Comparison between expression timeseries in AP2012 and its extended S-System formulation AP2012S. AP2012S was optimised to training data generated from AP2012 in a simulated 12L:12D → LL transition (B). For validation, the behaviour of the models was then evaluated in a simulated 12L:12D → DD transition (C). Timeseries are presented as heatmaps in which each gene/protein is grouped together, with the top and bottom rows in each group showing AP2012 and AP2012S expression levels, respectively. To aid visualisation, each timeseries has been normalised to its maximum value. White and black bars represent intervals of light and dark.

The circuit diagram for AP2012 is shown in Fig 3A. AP2012 is described by 28 ODEs. Heatmaps comparing the normalised dynamics of the model and its extended S-System formulation are shown in Fig 3B and 3C, with the corresponding unnormalised timeseries given in S3 and S4 Figs. In addition to the light-sensitive protein, we also omit the heatmap and timeseries of all COP1-related proteins in our plots, as these are not part of the core plant circadian network.

Like JL2005S and JL2006S, AP2012S displays excellent agreement on the training dataset. For the validation dataset, despite the simulated genes/proteins in AP2012S having similar peak and trough phases to their counterparts in AP2012, and a correspondingly small fitting error (*cf*. S10 Table), the extended S-System formulation generates an oscillation in DD that decays faster than the original formulation.

The parameter variability analysis of AP2012S shows that the optimisation process yields fairly constrained fits for this model also: the vast majority of parameters (96/115) have nMAD values less than 0.5 and the largest nMAD value is ≈0.94 (see S12C Fig and S9 Table).

#### KF2014

**Fig 4:**
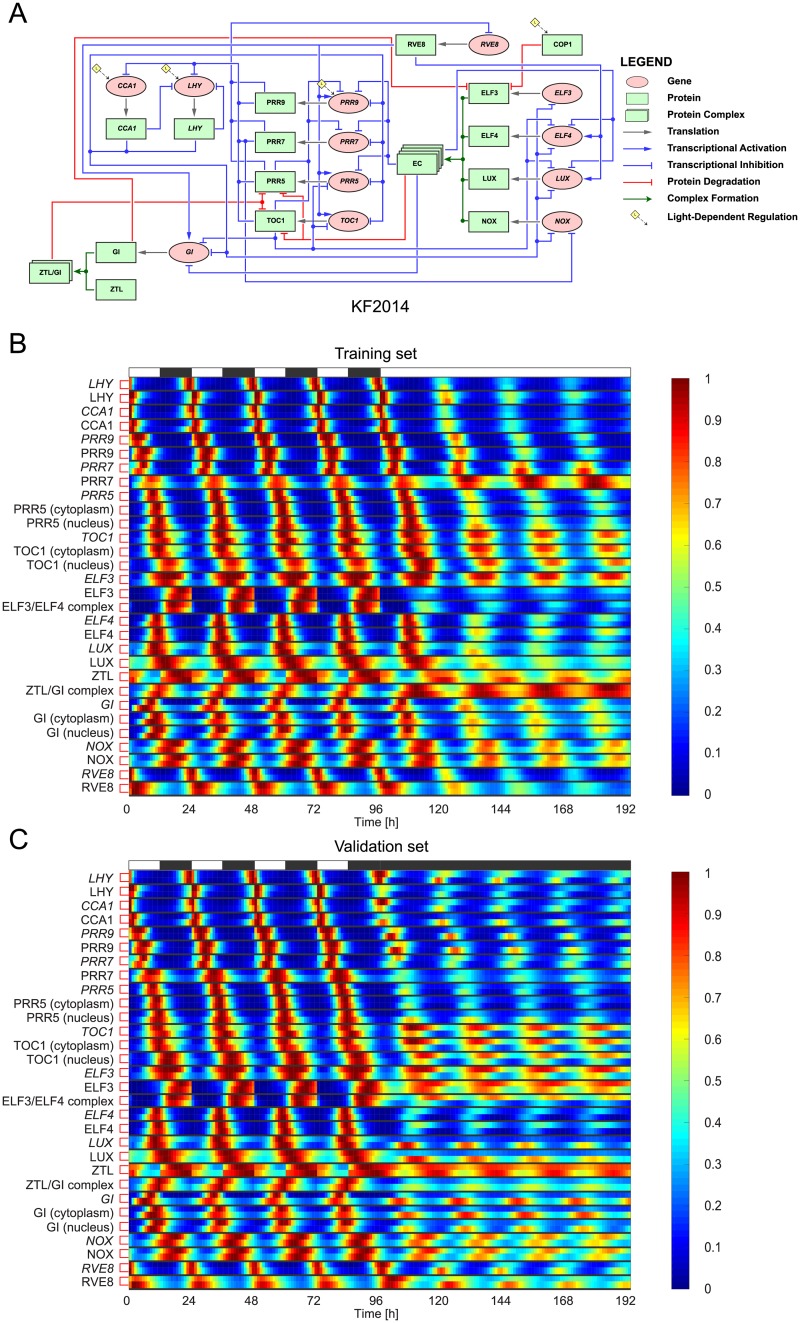
The KF2014S plant clock model—Fits to synthetic data. **A**: Regulatory circuit diagram for KF2014 [45]. The same symbols were used as in Fig 1. **B-C**: Comparison between expression timeseries in KF2014 and its extended S-System formulation KF2014S. KF2014S was optimised to training data generated from KF2014 in a simulated 12L:12D → LL transition (B). For validation, the behaviour of the models was then evaluated in a simulated 12L:12D → DD transition (C). Timeseries are presented as heatmaps in which each gene/protein is grouped together, with the top and bottom rows in each group denoting KF2014 and KF2014S expression levels, respectively. To aid visualisation, each timeseries has been normalised to its maximum value. White and black bars represent intervals of light and dark.

The fourth model, KF2014 [45], is the most comprehensive plant clock model available to date: its circuit diagram is shown in Fig 4A. KF2014 was constructed and validated against a large number of experiments reported in the literature (approximately 800 timeseries datasets spanning 59 published papers [45]). This results in the model being able to reproduce experimental findings across a broad range of different conditions. KF2014 is described by 35 ODEs. Heatmaps comparing the normalised dynamics of KF2014 and KF2014S are shown in Fig 4B and 4C (for the corresponding unnormalised timeseries, see S5 and S6 Figs). Like AP2012, the light-sensitive protein and all COP1-related proteins are omitted in our timeseries and heatmap plots.

For the training data, the heatmaps and timeseries indicate good agreement between the expression dynamics of the two models. For the validation set, both models show good agreement for the majority of components, with the exception of ELF3/ELF4 complex, *LUX* mRNA, LUX protein, *GI* mRNA, GI cytoplasmic protein and GI nuclear protein (*cf*. S6 Fig and S13 Table). However, the difference lies mainly in the amplitudes—the timing of peak and trough expression is similar in both models.

Finally, the variability in optimised parameter values is comparable to that observed for AP2012S, with a similar proportion of parameters (127/152) having nMAD values less than 0.5 and the maximum nMAD value being ≈0.94 (see S12D Fig and S12 Table).

### Fits to experimental data

#### MF2016K

**Fig 5:**
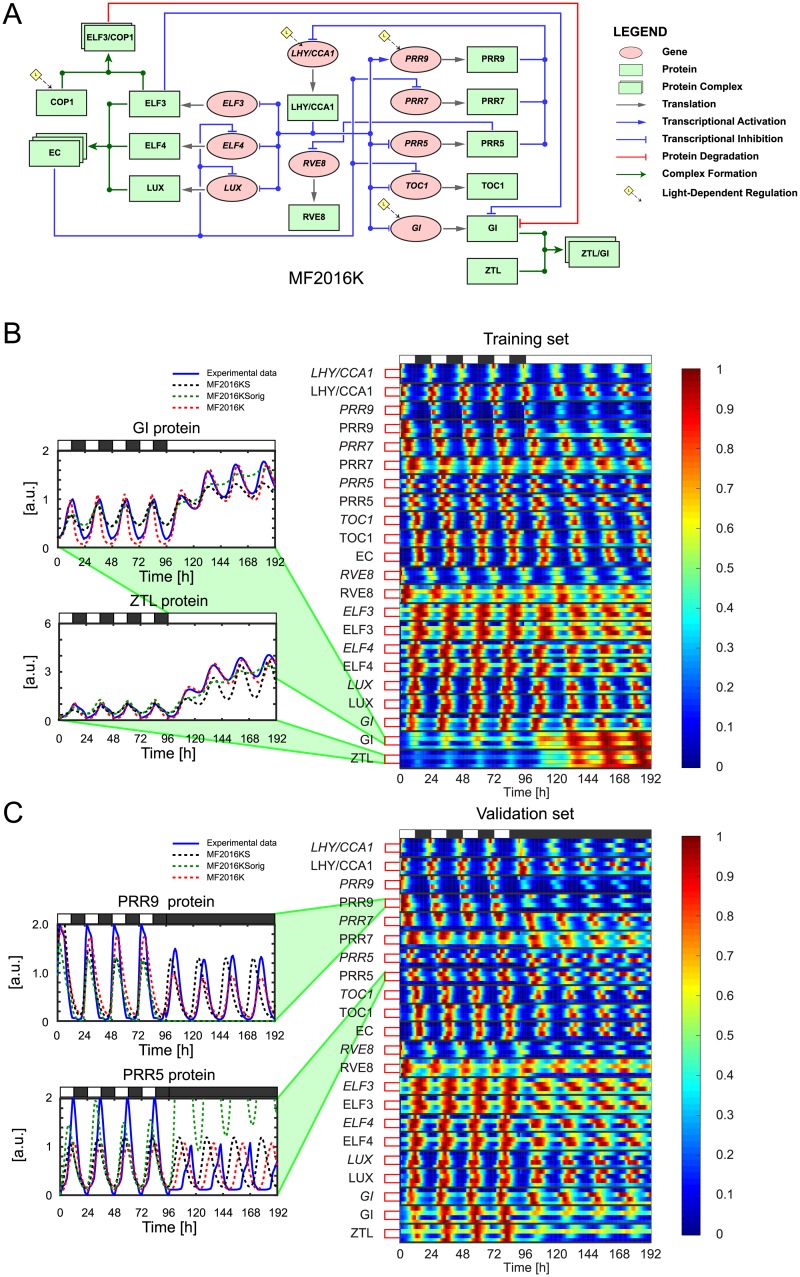
The MF2016KS, MF2016K and MF2016KSorig plant clock models—Fits to experimental data. **A**: Regulatory circuit diagram for MF2016K [15]. The same symbols were used as in Fig 1. **B-C**: Comparison between experimental expression timeseries and the corresponding simulations generated by the extended S-System formulation MF2016KS, the Michaelis-Menten formulation MF2016K and the original S-System formulation MF2016KSorig. The models were optimised to experimental data recorded in a 12L:12D → LL transition (B). For validation, the behaviour of each model in a simulated 12L:12D → DD transition was then evaluated against experimental data recorded in the same conditions (C). Timeseries are presented as heatmaps in which each gene/protein is grouped together, with the first row in each group showing experimental expression levels and the second, third and fourth rows showing simulated expression levels from models MF2016KS, MF2016K and MF2016KSorig, respectively. To aid visualisation, each timeseries has been normalised to its maximum value. White and black bars represent intervals of light and dark. In order to highlight the differences in predictive capacity between the three models, (B) shows the unnormalised expression timeseries for GI and ZTL proteins, while (C) shows the unnormalised expression timeseries for PRR9 and PRR5 proteins.

Here, we assess the ability of the extended S-System formulation to reproduce experimental data, where the modelled interaction between circadian genes follows the kernel version of the ODE system developed in [15]. The kernel model MF2016K shown in Fig 5A is described by 24 ODEs. Fig 5B and 5C show heatmaps comparing the normalised expression timeseries of the experimental datasets to the fits obtained previously with MF2016K in [15] and the fits obtained in this study with the extended/original S-System formulations of the model (MF2016KS/MF2016KSorig). The unnormalised timeseries for all three models are plotted in S7 and S8 Figs. The parameter sets yielding the best fits to experimental data are presented in S14 Table (MF2016KS), S16 Table (MF2016KSorig) and S17 Table (MF2016K), whilst the corresponding WMSE values are given in Table 1 (all models) and S15 Table (detailed cost breakdown for MF2016KS).

**Table 1:** Ranking model fits to experimental data. Here, *N*_*G*_ and *N*_*T*_ are the number of gene/protein timeseries and the number of timepoints per timeseries used for fitting, respectively. $W(D_{LL},{\hat{X}}_{LL}({\hat{\Theta }}_{LL}^{E}))$ is the weighted mean squared error (WMSE) of the best fit to the training data and $W(D_{DD},{\hat{X}}_{DD}({\hat{\Theta }}_{LL}^{E}))$ is the corresponding WMSE value of the fit to the validation data. Δ_*i*_(AIC) and *w*_*i*_(AIC) denote the AIC differences and Aikake weights for each model, respectively, calculated using Eqs (31)–(33).

	Model		
	MF2016KS	MF2016KSOrig	MF2016K
*N*_*G*_	24		
*N*_*T*_	194		
*K*_Θ_	72	78	77
$W(D_{LL},{\hat{X}}_{LL}({\hat{\Theta }}_{LL}^{E}))$	0.03647	0.04461	0.03649
$W(D_{DD},{\hat{X}}_{DD}({\hat{\Theta }}_{LL}^{E}))$	0.13557	1.70248	0.05629
Δ_*i*_(AIC)	0	950.03	12.55
*w*_*i*_(AIC)	0.99812	0	0.00188

For the training dataset, the heatmaps and timeseries indicate good agreement between both MF2016KS and the experimental data, with the extended S-System model yielding very similar expression dynamics to the Michaelis-Menten formulation (see Fig 5B and S7 Fig). The comparable performance between the models in reproducing the experimental data is reflected by their near-identical WMSE values, although MF2016KS has fewer parameters than MF2016K (see Table 1). The standard S-System formulation, MF2016KSorig, did not reproduce the behaviour of either GI or ZTL protein (see Fig 5B and S7 Fig). Thus, despite being able to adequately reproduce the dynamics of other genes and proteins, MF2016KSorig has a larger WMSE value than the other two models.

The AIC values in Table 1 quantify this comparison in model performance, with MF2016KS, MF2016K and MF2016KSorig yielding Aikake weights of *w*_KS_ = 0.9981, *w*_K_ = 0.0019 and *w*_KSorig_ = 0, respectively. These weights exclude MF2016KSorig as a viable candidate for the best model (in the sense of K-L divergence) and imply strongly that the best model is MF2016KS (the evidence ratio $\frac{w_{\text{KS}}}{w_{K}}$ indicates that it is 530 times more likely to be so than MF2016K).

For the validation dataset, although the simulated MF2016KS waveforms (and hence amplitudes and periods) of most circadian components are close to those of the experimental timeseries, peak expression occurs earlier in the model following release from LD into DD (see Fig 5C and S8 Fig). In addition, for ZTL protein, the MF2016KS timeseries has a larger amplitude than the experimental expression profile. MF2016K yields a better fit to the data in this case (particularly to ZTL protein), as reflected by its lower WMSE value (*i.e*. its lower out-of-sample error). MF2016KSorig has a high out-of-sample error, predominately due to some simulated PRR9, PRR7 and PRR5 components having much higher amplitudes than their experimental counterparts in DD (see Fig 5C and S8 Fig).

The parameter variability analysis of MF2016KS mirrors the general trend observed in the fits of the larger models to synthetic data. The overwhelming majority of parameters (63/72) have nMAD values less than 0.5 and the largest nMAD value is ≈0.93 (see S12E Fig and S14 Table).

### The aggregated protein model—Linear systems analysis

As noted earlier, the later plant clock models (*e.g*. AP2012 and KF2014) used a single equation to represent the production of TF, except in cases where experimental data was available that distinguished between cytoplasmic and nuclear forms (*e.g*. ELF3 and GI in [53] and PRR5, TOC1 and GI in [54]). This was in contrast to the paired equations used to represent protein shuttling between cytoplasm and nucleus that were employed in the earlier models (*e.g*. JL2005 and JL2006).

To understand why the practice of aggregating protein forms did not appear to adversely affect the predictive capacity of the later models, we used sine-sweeping—a frequency response analysis method from linear systems theory [55]—to approximate the transfer functions describing the production of TF in the models that explicitly describe transport between cytoplasmic and nuclear compartments. Sine-sweeping is widely used in the field of system identification to obtain simplified empirical transfer functions. The method is applicable when the system of interest displays linear dominant behaviour (*i.e*. when the response to a sinusoidal input signal is itself sinusoidal, with the same input frequency and some phase shift). For a linear dominant system, sine-sweeping provides a means of obtaining an approximate, simplified linear model in the event that the linearisation of the full nonlinear model is not straightforward. When we applied the method to the plant clock models incorporating protein shuttling, JL2005 and JL2006, we observed linear dominant behaviour in each case, thereby validating the approach. Here, we present the results obtained for JL2006 (similar results were obtained for JL2005).

**Fig 6:**
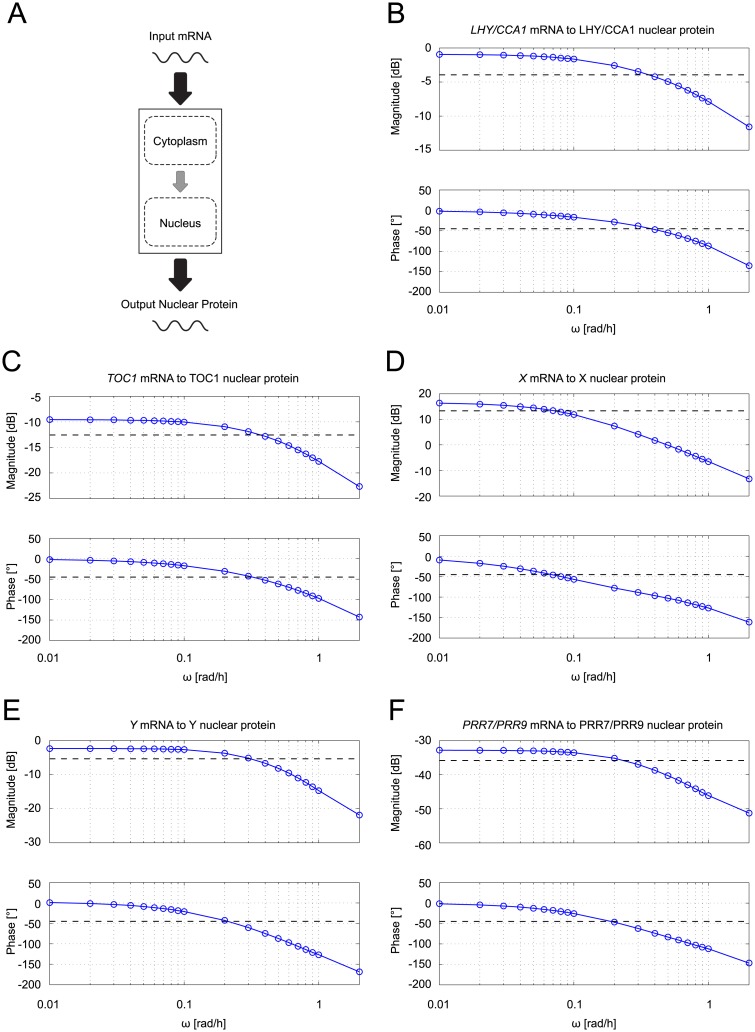
Frequency response analysis of protein shuttling in JL2006. **A**: For each circadian gene, a sinusoidal mRNA signal with varying frequencies is provided as the input to the system and the nuclear protein is observed as the resulting output. **B-F**: Bode plots obtained from sine-sweeping the five different circadian genes of the model. In each magnitude plot, the black dashed line represents the magnitude that is 3dB lower than that at *ω* = 0. For each phase plot, the black dashed line represents a phase shift of -45°. For each gene, if the protein shuttling mechanism can be approximated by a first-order transfer function, then the intersections of the blue solid lines and the black dashed lines in both the magnitude and phase plots should occur at the same frequency. **B**: *LHY/CCA1* mRNA ↦ LHY/CCA1 nuclear protein. **C**: *TOC1* mRNA ↦ TOC1 nuclear protein. **D**: *X* mRNA ↦ X nuclear protein. **E**: *Y* mRNA ↦ Y nuclear protein. **F**: *PRR7/PRR9* mRNA ↦ PRR7/PRR9 nuclear protein.

In applying sine-sweeping to the protein shuttling mechanism, the system input is the mRNA expression timeseries and the system output is the resulting nuclear protein expression timeseries (see Fig 6A). We then drive the system with sinusoidal input signals with frequencies in the range 0.01 rad/h to 2 rad/h. From systems theory, it follows that if the system is linear (or linear dominant), the output obtained for a sinusoid of given frequency will also be sinusoidal with the same frequency, but with a scaled amplitude and phase shift. By calculating the amplitude scaling and phase shifts over the frequencies of interest, we obtain a Bode plot. This in turn allows us to approximate the transfer function of the system, which enables the system’s response to any input signal (*i.e*. any mRNA expression timeseries) to be estimated (see section 2 in S1 Text and S9 Fig). The order *N* of the transfer function is of particular importance in our case, as it specifies the minimum number of linear differential equations required to represent the system. *N* can be approximated from the Bode plot by exploiting the fact that for an *N*th order transfer function, the slope of the magnitude plot at the corner frequency is (−20 × *N*) dB/decade and the phase shift at this frequency is (−45 × *N*)° (the corner frequency is defined as the frequency at which the magnitude plot has decreased by 3dB from its plateau level—for more details, see [56]).

The protein shuttling equations in JL2006 have the general form given in Eq (8) but with nonlinear rather than linear degradation terms (*cf*. eqs. (S1.3) in S1 Text). The Laplace transforms of the linearised protein shuttling equations in JL2006 yield second-order transfer functions, as detailed in section 3 of S1 Text. For each gene, we would therefore expect the Bode plot obtained with sine-sweeping to have a slope of -40 dB/decade at the corner frequency and a corresponding phase shift of -90°. However, as can be seen in Fig 6B–6F, for all the circadian genes, the phase shift at the corner frequency is instead close to -45° and the corresponding slope in the magnitude plot is close to -20 dB/decade (with the exception of *LHY/CCA1*, for which the slope is approximately -10 dB/decade). Thus, the protein shuttling mechanism in each case appears to be quite well approximated with a first-order transfer function.

To understand the reason for this observed difference, we further analysed the protein equations used for JL2006. A second order system can be approximated by a first-order system if the two poles (*i.e*. the roots of the denominator of the transfer function) are far apart—in other words, if the system has one fast pole and one slow pole. Using gene *Y* as a case study, we found that the two poles are indeed far apart (see eq. (S3.5) in S1 Text and S10 Fig), with one pole having a value approximately 30 times greater than the other. As shown in S11 Fig, this scenario results in the second-order transfer function quite closely resembling a first-order transfer function, which implies that a single, aggregated protein equation is sufficient to represent the protein translation pathway (see eqs. (S3.6) and (S3.7) in S1 Text).

## Discussion

### Extended S-System formulations can reproduce the dynamics of existing plant clock models and experimental data

In this study, we have investigated the use of a simplified modelling framework based on S-Systems to describe the behaviour of the plant circadian clock. We tested the efficacy of this new approach by constructing the extended S-System formulations of five different existing plant clock models. Four of these models—the extended S-System versions of JL2005 [11], JL2006 [43], AP2012 [44] and KF2014 [45]—were optimised to synthetic training data generated from the original models, whilst the extended S-System version of the fifth model—MF2016K [15]—was optimised to experimental training data. To assess predictive capacity, the goodness-of-fit obtained for each model on a validation dataset (*i.e*. one distinct from the training data) was then computed.

For the two simplest models considered, JL2005 and JL2006, very close agreement was observed between the extended S-System formulations and the original models for both the training and validation datasets (Figs 1 and 2, S1 and S2 Figs), with near-identical simulated and target expression timeseries in some of the model components. For AP2012 and KF2014, which incorporate a greater number of genetic components and more complex regulation mechanisms, excellent fits were again observed for the training data (Figs 3B and 4B, S3 and S5 Figs). On validation data, whilst AP2012S gives a close match to AP2012 during the light-dark cycle, the extended S-System formulation generates a much more pronounced damping (with phase shifts in some components) following release into constant dark (Fig 3C and S4 Fig). Similarly to AP2012S, KF2014S generates timeseries that closely match KF2014 in the LD portion of the validation dataset, with a more pronounced deviation between the models observed following DD release (Fig 4C and S6 Fig).

Interestingly, despite AP2012S’s poorer fit to validation data, a comparable predictive performance to AP2012 is observed when qualitatively modelling short- and long-period mutant phenotypes. This can be seen in Table 2, which compares the predicted period phenotypes generated by the two model formulations against the corresponding experimentally-observed phenotype for a range of knockout/knockdown and overexpression mutants in different light conditions. Indeed, the table shows that both AP2012 and its extended S-System formulation correctly simulate 10/16 phenotypes (albeit not all the same ones), despite the latter not having been fitted to any mutant data.

**Table 2:** Comparisons between simulated and experimental free-running period shifts *δτ* in different constant light conditions and genetic backgrounds for plant models AP2012 and AP2012S. For all light-mutant combinations considered, *δτ* was calculated as the difference between the mutant and wild-type periods: *δτ* = *δτ*_*mut*_ − *δτ*_*wt*_. Hence, *δτ* < 0 corresponds to a short-period mutant (denoted by a − sign) and *δτ* > 0 corresponds to a long-period mutant (denoted by a + sign). The study providing the experimental values used to calculate *δτ* is reported in the rightmost column in each case. In the above, Δ denotes knockout/knockdown, ‘OX’ denotes overexpression, ‘arr.’ denotes an arrhythmic oscillation, LL denotes constant light and DD denotes constant dark. Knockout/knockdown mutant behaviour was simulated by reducing the transcription rate of the target TF by 80%, with the exception of the ZTL mutant for which the protein production rate constant was decreased by 80% instead (there is no term for *ZTL* mRNA production in AP2012—see eqs. (S1.5) in S1 Text). Overexpression mutant behaviour was simulated by increasing the translation rate of the target TF two-fold. Simulated periods were calculated by using the MATLAB function findpeaks to obtain all the differences between two successive maxima in the circadian rhythm, and then averaging across all circadian gene components.

Mutant	Light condition	sgn(*δτ*): exp.	sgn(*δτ*): simulated		Citation
			(AP2012S)	(AP2012)	
Δ*lhy*/*cca*1	LL	-	+	-	[57]
Δ*toc*1	LL	-	-	-	[58]
Δ*prr*7	LL	+	-	+	[59]
Δ*prr*9	LL	+	+	-	[59]
Δ*prr*9*prr*7	LL	+	+	+	[59]
Δ*ztl*	LL	+	+	+	[60]
Δ*gi*	LL	-	-	-	[61]
Δ*elf*4	LL	arr.	arr.	arr.	[62]
Δ*elf*3	LL	arr.	arr.	arr.	[63]
Δ*lux*	LL	arr.	arr.	arr.	[64]
ELF4-OX	LL	+	-	-	[65]
ELF3-OX	LL	+	-	-	[65]
Δ*gi*	DD	arr.	-	-	[66]
Δ*elf*4	DD	arr.	arr.	+	[67]
Δ*ztl*	DD	+	+	+	[68]
ELF3-OX	DD	+	-	-	[65]

In terms of experimental data-fitting, the results obtained for MF2016KS mirrored those of the more complex models on synthetic data. The extended S-System model generates expression timeseries that give good matches to the training data (Fig 5B and S7 Fig). Moreover, the extended S-System formalism yielded a superior model in this case, compared to both the original Michaelis-Menten formulation and a model based on the standard S-System framework, as quantified by an AIC analysis (Table 1). Similarly to AP2012S and KF2014S, however, a greater discrepancy between MF2016KS and data is observed during the LD to DD transition, predominately characterised by a phase shift (Fig 5C and S8 Fig). The Michaelis-Menten model gives a better fit in this case, albeit with a larger number of parameters.

### The extended S-System framework facilitates model development, network inference and synthetic circuit design

The results of our fits to synthetic and experimental data demonstrate that the extended S-System formulation is capable of yielding models with comparable predictive capacity to a set of canonical plant clock models. Furthermore, this approach confers several advantages for GRN modelling compared to the conventional Michaelis-Menten based framework that is predominately used in computational circadian biology.

The first such advantage relates to model development and expansion. The extended S-System formulation enables new interactions to be added easily, as depending on whether such an interaction affects the production, degradation or light regulation of the target component, it can simply be incorporated into the corresponding term of Eq (2) through an appropriate choice of coefficients, exponents and upper product/summation bounds. Conversely, interactions can also be removed in a straightforward manner.

The second advantage relates to network inference, which is the concomitant of the first advantage. In the extended S-System formalism, the type of regulation implemented by a given network component is simply determined by the sign of the corresponding exponent *g*_*i*,*j*_ in the first term of Eq (2), with *g*_*i*,*j*_ < 0 indicating an inhibitor and *g*_*i*,*j*_ > 0 indicating an activator. The regulation type can therefore be inferred together with all the other parameters specifying Eq (2) during data-fitting, without having to modify the production term. Indeed, for all the data-fitting presented here, no constraints were imposed on the *g*_*i*,*j*_s for the initial optimisations to training data. For synthetic data-fitting, the inferred patterns of activation and inhibition were checked against the corresponding model, whilst for optimisation to experimental data, the activation/inhibition pattern was checked against the experimental literature. In each case, almost all signs were correctly inferred. In the event that one or more signs were incorrectly identified, these were reversed and a further optimisation run was performed.

To put these two advantages into perspective, in order to add a new interaction to one of the plant clock models using the Michaelis-Menten model structure, we would need to specify *a priori* whether this was a positive or negative interaction, given the different functions used to represent activation and inhibition (*cf*. Eqs (5) and (6)) and then estimate the two new parameters associated with this interaction. If the interaction type was unknown, two separate optimisations would be required. On the other hand, using the extended S-System formulation, we would only require an estimate of the exponent associated with the new interaction (*cf*. Eq (2)) and if the interaction type were unknown, a single optimisation would be sufficient to infer it. In this vein, recent experimental work appears to imply a switch in LHY ↦ *PRR9* regulation from activation to inhibition [69]. Revising this interaction could be done in a model based on the extended S-System formalism in a very straightforward manner, by reversing the sign of the exponent associated with the interaction and then reoptimising parameters. In addition, the interaction type (activation or inhibition) that was predicted to give the best fit to data could be established by simply leaving the sign of the exponent unconstrained during the parameter optimisation process.

A third key advantage relates to the design of synthetic feedback control circuits for mitigating perturbations to GRNs (*e.g*. changes to steady-state expression levels associated with infection). Indeed, our previous work [70] has shown that using Michaelis-Menten based models to obtain accurate simulations of closed-loop control strategies requires *consistent* estimates of the Michaelis constants for all components affected by the control signal(s) (here, by consistent we mean multiple optimisation runs started from different initial conditions locate similar parameter values that reproduce the data). This is because variability in these estimates can lead to a simulated saturated response in the relevant components (*i.e*. if *P* ≫ *K* in Eqs (5) and (6)). This in turn can result in an inaccurate prediction of controller behaviour, as shown in Figure 3 of [70]. However, the propensity of Hill functions to generate a saturated response means that, in practice, optimisations to data can lead to large variations in the inferred values of the corresponding Michaelis constants—in other words, models utilising such functions can suffer from poor parameter identifiability.

Finally, although our previous analysis concurred with [71] that accurately estimating exponents in power-law based models can be challenging (see Figures 4 and 7 of [70]), it also showed that these estimates are more consistent than those obtained for the corresponding parameters in Michaelis-Menten based models. This suggests that the S-System formalism can mitigate the parameter identifiability issue, identifying the approach as a viable complementary modelling framework for designing robust synthetic controllers.

### Future directions

In addition to demonstrating the significant potential of extended S-Systems for plant clock modelling, our work also provides potential insight into the shift from representing protein pathways with two equations for separate cytoplasmic and nuclear compartments (*e.g*. JL2005, JL2006), to a single, aggregated equation (*e.g*. AP2012, KF2014, MF2016) in the established suite of *Arabidopsis* models. Specifically, our sine sweeping results show that in the models employing two pathway equations, the resulting second-order transfer function relating mRNA to active protein can be well-approximated with a first-order transfer function, owing to the two poles being far apart (*i.e*. due to time-scale separation), as shown in Fig 6 and S11 Fig. A single pathway equation would therefore have yielded the same results in each case, implying that in the construction of these models, the data-fitting procedure did not exploit the greater flexibility conferred by the second-order transfer function and/or the increased flexibility was not required to obtain good fits. This apparent redundancy, together with a desire for reduced parametrisations, may partly have driven the subsequent models to jettison separate compartments. It would therefore be of interest to extend our extended S-System framework to integrate distributed delay-based models of protein pathways [19, 72, 73], as these yield tunable transfer functions of arbitrary order, whilst maintaining a compact parametrisation [16]. In addition, although our modification to the original S-System framework extends the range of transcriptional regulation mechanisms that can be modelled beyond the multi-input AND gate of the original formulation (*cf*. Eqs (1) and (2)), it still does not cover the full range of biologically realistic logic gates. This limitation could be addressed by modifying the first term of Eq (2) to encode an S-System implementation of the nested canalysing Boolean functions associated with $n_{i}^{P}$ inputs [74]. It would be instructive to investigate whether such extensions to the S-System framework resulted in models yielding more accurate fits to data (*e.g*. the fits of MF2016KS to experimental DD release recordings), but with reduced complexity compared to Michaelis-Menten based models.

Finally, we note that although we have focused here on plant circadian clock models, the approach presented is potentially applicable to all GRNs characterised by entrainable, periodic oscillations.

## Supporting information

S1 Text Supporting Text. (PDF)

S1 Fig Plant clock model JL2006S—Optimal fits to synthetic training data. Blue solid lines show timeseries generated by JL2006 from its nominal parameter values for a simulated transition between a 12L:12D light-dark cycle and constant light (LL). Black dashed lines show timeseries obtained by optimising the parameters of JL2006S to this data in the same simulated light environment (see Fig 2B for the corresponding heatmaps). White and black bars at the top of the figure indicate light and dark intervals, respectively.
(EPS)

S2 Fig Plant clock model JL2006S—Fits to synthetic validation data. Blue solid lines show timeseries generated by JL2006 from its nominal parameter values for a simulated transition between a 12L:12D light-dark cycle and constant dark (DD). Black dashed lines show timeseries generated by JL2006S in the same simulated light environment using the parameters optimised to the training data (see Fig 2C for the corresponding heatmaps). White and black bars at the top of the figure indicate light and dark intervals, respectively.
(EPS)

S3 Fig Plant clock model AP2012S—Optimal fits to synthetic training data. Blue solid lines show timeseries generated by AP2012 from its nominal parameter values for a simulated transition between a 12L:12D light-dark cycle and constant light (LL). Black dashed lines show timeseries obtained by optimising the parameters of AP2012S to this data in the same simulated light environment (see Fig 3B for the corresponding heatmaps). White and black bars at the top of the figure indicate light and dark intervals, respectively.
(EPS)

S4 Fig Plant clock model AP2012S—Fits to synthetic validation data. Blue solid lines show timeseries generated by AP2012 from its nominal parameter values for a simulated transition between a 12L:12D light-dark cycle and constant dark (DD). Black dashed lines show timeseries generated by AP2012S in the same simulated light environment using the parameters optimised to the training data (see Fig 3C for the corresponding heatmaps). White and black bars at the top of the figure indicate light and dark intervals, respectively.
(EPS)

S5 Fig Plant clock model KF2014S—Optimal fits to synthetic training data. Blue solid lines show timeseries generated by KF2014 from its nominal parameter values for a simulated transition between a 12L:12D light-dark cycle and constant light (LL). Black dashed lines show timeseries obtained by optimising the parameters of KF2014S to this data in the same simulated light environment (see Fig 4B for the corresponding heatmaps). White and black bars at the top of the figure indicate light and dark intervals, respectively.
(EPS)

S6 Fig Plant clock model KF2014S—Fits to synthetic validation data. Blue solid lines show timeseries generated by KF2014 from its nominal parameter values for a simulated transition between a 12L:12D light-dark cycle and constant dark (DD). Black dashed lines show timeseries generated by KF2014S in the same simulated light environment using the parameters optimised to the training data (see Fig 4C for the corresponding heatmaps). White and black bars at the top of the figure indicate light and dark intervals, respectively.
(EPS)

S7 Fig Plant clock models MF2016KS, MF2016KSorig and MF2016K—Optimal fits to experimental training data. Blue solid lines show timeseries recorded experimentally during a transition between a 12L:12D light-dark cycle and constant light (LL). Black (MF2016KS) and green (MF2016KSorig) dashed lines show timeseries obtained by optimising the parameters of the S-System models to this data in the same simulated light environment. Red dashed lines show optimal fits of MF2016K to the same data, obtained previously in [15] (see Fig 5B for the corresponding heatmaps). White and black bars at the top of the figure indicate light and dark intervals, respectively.
(EPS)

S8 Fig Plant clock models MF2016KS, MF2016KSorig and MF2016K—Fits to experimental validation data. Blue solid lines show timeseries recorded experimentally during a transition between a 12L:12D light-dark cycle and constant dark (DD). Black (MF2016KS) and green (MF2016KSorig) dashed lines show timeseries generated by the S-System models in the same simulated light environment using the parameters optimised to the training data. Red dashed lines show the corresponding fits of MF2016K to the same data (see Fig 5C for the corresponding heatmaps). White and black bars at the top of the figure indicate light and dark intervals, respectively.
(EPS)

S9 Fig Correlation method. The output of the sine sweeping test *y*(*t*) is correlated with sin *ωt* and cos *ωt* prior to averaging to obtain the corresponding magnitude and phase values required to construct a Bode plot.
(EPS)

S10 FigLinear approximations to nonlinear Y protein degradation in JL2006.Blue lines show how the degradation rates of cytoplasmic Y protein (top panel) and nuclear Y protein (bottom panel) depend on the corresponding expression levels, $c_{Y}^{\left(c\right)}$ and $c_{Y}^{\left(n\right)}$, respectively. In each case, degradation rate is plotted for expression levels ranging between 0 and the maximum level observed in the synthetic training and validation datasets (see S1 and S2 Figs). In these ranges, the nonlinear functions are well-approximated by linear fits (red lines), the gradients of which are taken as the values of *γ*_*cy*_ and *γ*_*nu*_ used to derive eq. (S3.5) in S1 Text.(EPS)

S11 Fig Bode plot relating input *Y* mRNA to output Y nuclear protein in JL2006. Blue lines represent the second-order system given by eq. (S3.5) in S1 Text. Red lines represent the first-order system given by eq. (S3.7) that approximates eq. (S3.5).
(EPS)

S12 Fig Variation in optimised parameter values for the extended S-System models. **A-D**: Fits of JL2005S, JL2006S, AP2012S and KF2014S to synthetic data. **E**: Fits of MF2016KS to experimental data. Boxplots show parameter distributions obtained from six independent optimisation runs. In each boxplot, the horizontal line denotes the median value, the edges of the box are the 25th and 75th percentiles, the whiskers denote the most extreme datapoints not considered to be outliers, and outliers are plotted as red crosses. Model parameter indices are defined in S3 Table (JL2005S), S6 Table (JL2006S), S9 Table (AP2012S), S12 Table (KF2014S) and S14 Table (MF2016KS). In **B-E**, the thick black horizontal lines separate parameters whose values are plotted with respect to the left and right y-axes.
(EPS)

S1 Table Variables used in the equations for plant clock models JL2005 [11], JL2006 [43], AP2012 [44], KF2014 [45] and MF2016K [15]. (EPS)

S2 Table Nominal parameter values for JL2005, which were used to generate synthetic data. The parameters were taken from Figure 5 of [11].
(EPS)

S3 TableOptimal parameter values ${\hat{\Theta }}_{LL}^{S}$ for the extended S-System formulation JL2005S of JL2005, obtained by fitting the model to the synthetic training data.For each parameter, the number in brackets is the normalised median absolute deviation (nMAD). This is calculated using the value shown, together with those obtained from five additional, independent optimisation runs. The rightmost column shows the parameter indexing, counting left to right across rows, that is used in S12 Fig.(EPS)

S4 Table The component-wise (*W*_*i*_) and total (*W*) weighted mean squared error (WMSE) values obtained when fitting JL2005S to the synthetic training and validation datasets. (EPS)

S5 Table Nominal parameter values for JL2006, which were used to generate synthetic data. The parameter values were taken from inline Supplementary Table 1 of [43].
(EPS)

S6 TableOptimal parameter values ${\hat{\Theta }}_{LL}^{S}$ for the extended S-System formulation JL2006S of JL2006, obtained by fitting the model to the synthetic training data.For each parameter, the number in brackets is the normalised median absolute deviation (nMAD). This is calculated using the value shown, together with those obtained from five additional, independent optimisation runs. The rightmost column shows the parameter indexing, counting left to right across rows, that is used in S12 Fig.(EPS)

S7 Table The component-wise (*W*_*i*_) and total (*W*) weighted mean squared error (WMSE) values obtained when fitting JL2006S to the synthetic training and validation datasets. (EPS)

S8 Table Nominal parameter values for AP2012, used to generate synthetic data. The parameter values were taken from Supplemental Table 1 of [44]. (EPS)

S9 TableOptimal parameter values ${\hat{\Theta }}_{LL}^{S}$ for the extended S-System formulation AP2012S of AP2012, obtained by fitting the model to the synthetic training data.For each parameter, the number in brackets is the normalised median absolute deviation (nMAD). This is calculated using the value shown together with those obtained from five additional, independent optimisation runs. The rightmost column shows the parameter indexing, counting left to right across rows, that is used in S12 Fig.(EPS)

S10 Table The component-wise (*W*_*i*_) and total (*W*) weighted mean squared error (WMSE) values obtained when fitting AP2012S to the synthetic training and validation datasets. (EPS)

S11 Table Nominal parameter values for KF2014, which were used to generate synthetic data. The parameter values were taken from Table 3 (Parameter Set 2)^†^ and Table 4 in Supporting Information Text S1 of [45] (^†^We note that in the original paper, Table 3 incorrectly lists parameter *a*_3_ as *a*_1_—this has been fixed in our version of the table).
(EPS)

S12 TableOptimal parameter values ${\hat{\Theta }}_{LL}^{S}$ for the extended S-System formulation KF2014S of KF2014, obtained by fitting the model to the synthetic training data.For each parameter, the number in brackets is the normalised median absolute deviation (nMAD). This is calculated using the value shown, together with those obtained from five additional, independent optimisation runs. The rightmost column shows the parameter indexing, counting left to right across rows, that is used in S12 Fig.(EPS)

S13 Table The component-wise (*W*_*i*_) and total (*W*) weighted mean squared error (WMSE) values obtained when fitting KF2014 to the synthetic training and validation datasets. (EPS)

S14 TableOptimal parameter values ${\hat{\Theta }}_{LL}^{E}$ for the extended S-System formulation MF2016KS of MF2016K, obtained by fitting to the experimental training data.For each parameter, the number in brackets is the normalised median absolute deviation (nMAD). This is calculated using the value shown, together with the values obtained from five additional, independent optimisation runs. The rightmost column shows the parameter indexing, counting left to right across rows, that is used in S12 Fig.(EPS)

S15 Table The component-wise (*W*_*i*_) and total (*W*) weighted mean squared error (WMSE) values obtained when fitting MF2016KS to the experimental training and validation datasets. (EPS)

S16 TableOptimal parameter values ${\hat{\Theta }}_{LL}^{E}$ for the original S-System formulation MF2016KSorig of MF2016K, obtained by fitting to the experimental training data.(EPS)

S17 Table Optimal parameter values for MF2016K, which were obtained previously in [15] by fitting to the experimental training data used in this study with the same optimisation method. The values are reproduced from Tables S2 and S4 in the Supporting Information of [15].
(EPS)
