# Supplementary material for: A simplified modelling framework facilitates more complex representations of plant circadian clocks
Source: PLoS Comput Biol. 2020 Mar 16;16(3):e1007671. doi: 10.1371/journal.pcbi.1007671 (PMC7098658; doi:10.1371/journal.pcbi.1007671)
Supplement: S1 Text — (PDF) [file pcbi.1007671.s001.pdf]

A simplified modelling framework facilitates more complex representations of plant circadian clocks

Mathias Foo[1], Declan G. Bates[2], Ozgur E. Akman[3*],

1 School of Mechanical, Aerospace and Automotive Engineering, Coventry University, Coventry, CV1 5FB, UK
2 Warwick Integrative Synthetic Biology Centre, School of Engineering, University of Warwick, Coventry, CV4 7AL, UK
3 College of Engineering, Mathematics and Physical Sciences, University of Exeter, Exeter EX4 4QF, UK

* O.E.Akman@exeter.ac.uk

Supporting Text

Contents

1 Model equations 1
1.1 JL2005: original equations . . . 2
1.2 JL2005S: extended S-System formulation . . . 2
1.3 JL2006: original equations . . . 2
1.4 JL2006S: extended S-System formulation . . . 3
1.5 AP2012: original equations . . . 4
1.6 AP2012S: extended S-System formulation . . . 6
1.7 KF2014: original equations . . . 7
1.8 KF2014S: extended S-System formulation . . . 9
1.9 MF2016K: original equations . . . 11
1.10 MF2016KS: extended S-System formulation . . . 12
1.11 MF2016KSorig: original S-System formulation . . . 13

2 The sine-sweeping method 14

3 Transfer function analysis of protein shuttling in JL2006 15

References 16

1 Model equations

In each model below, $c_i^{(m)}(t)$ and $c_i(t)$ denote the cellular concentration of the mRNA and protein of gene i, respectively. For the models where cytoplasmic and nuclear protein are treated separately, these two forms are labelled as $c_i^{(c)}(t)$ and $c_i^{(n)}(t)$, respectively. The different gene products labelled by i are listed in **S1 Table**. $c_P(t)$ (or $c_P^{(n)}(t)$) denotes the light-activated PIF3-like protein introduced originally in JL2005 [S1] and subsequently used in all the other clock models considered in this study. The light input, $L_I(t)$, is modelled as a periodic square wave that switches between 0 and 1 (see eq. (3) of the main paper).

1.1 JL2005: original equations

$$
\begin{aligned}
\frac{dc_L^{(m)}}{dt} &= q_1 c_P^{(n)} L_I(t) + \frac{n_1 {c_T^{(n)}}^a}{{g_1}^a + {c_T^{(n)}}^a} - \frac{m_1 c_L^{(m)}}{k_1 + c_L^{(m)}}, \\
\frac{dc_L^{(c)}}{dt} &= p_1 c_L^{(m)} - r_1 c_L^{(c)} + r_2 c_L^{(n)} - \frac{m_2 c_L^{(c)}}{k_2 + c_L^{(c)}}, \\
\frac{dc_L^{(n)}}{dt} &= r_1 c_L^{(c)} - r_2 c_L^{(n)} - \frac{m_3 c_L^{(n)}}{k_3 + c_L^{(n)}}, \\
\frac{dc_T^{(m)}}{dt} &= \frac{n_2 {g_2}^b}{{g_2}^b + {c_L^{(n)}}^b} - \frac{m_4 c_T^{(m)}}{k_4 + c_T^{(m)}}, \\
\frac{dc_T^{(c)}}{dt} &= p_2 c_T^{(m)} - r_3 c_T^{(c)} + r_4 c_T^{(n)} - \frac{m_5 c_T^{(c)}}{k_5 + c_T^{(c)}}, \\
\frac{dc_T^{(n)}}{dt} &= r_3 c_T^{(c)} - r_4 c_T^{(n)} - \frac{m_6 c_T^{(n)}}{k_6 + c_T^{(n)}}, \\
\frac{dc_P^{(n)}}{dt} &= p_3(1 - L_I(t)) - \frac{m_7 c_P^{(n)}}{k_7 + c_P^{(n)}} - q_2 c_P^{(n)} L_I(t).
\end{aligned} \tag{S1.1}
$$

1.2 JL2005S: extended S-System formulation

$$
\begin{aligned}
\frac{dc_L^{(m)}}{dt} &= \alpha_1 {c_T^{(n)}}^{g_{1,1}} - \beta_{1,1} c_L^{(m)} + \gamma_{1,1} c_P^{(n)} L_I(t), \\
\frac{dc_L^{(c)}}{dt} &= \alpha_2 {c_L^{(m)}}^{g_{2,1}} - \beta_{2,1} c_L^{(c)}, \\
\frac{dc_L^{(n)}}{dt} &= \alpha_3 {c_L^{(c)}}^{g_{3,1}} - \beta_{3,1} c_L^{(n)}, \\
\frac{dc_T^{(m)}}{dt} &= \alpha_4 {c_L^{(n)}}^{g_{4,1}} - \beta_{4,1} c_T^{(m)}, \\
\frac{dc_T^{(c)}}{dt} &= \alpha_5 {c_T^{(m)}}^{g_{5,1}} - \beta_{5,1} c_T^{(c)}, \\
\frac{dc_T^{(n)}}{dt} &= \alpha_6 {c_T^{(c)}}^{g_{6,1}} - \beta_{6,1} c_T^{(n)}, \\
\frac{dc_P^{(n)}}{dt} &= -\beta_{7,1} c_P^{(n)} + \gamma_{7,1}(1 - L_I(t)) + \gamma_{7,2} c_P^{(n)} L_I(t).
\end{aligned} \tag{S1.2}
$$

1.3 JL2006: original equations

$$
\begin{aligned}
\frac{dc_L^{(m)}}{dt} &= \frac{{g_0}^\alpha}{{g_0}^\alpha + {c_A^{(n)}}^\alpha} \cdot \left(\left(q_1 c_P^{(n)} + n_0 \right) L_I(t) + \frac{n_1 {c_X^{(n)}}^a}{{g_1}^a + {c_X^{(n)}}^a} \right) - \frac{m_1 c_L^{(m)}}{k_1 + c_L^{(m)}}, \\
\frac{dc_L^{(c)}}{dt} &= p_1 c_L^{(m)} - r_1 c_L^{(c)} + r_2 c_L^{(n)} - \frac{m_2 c_L^{(c)}}{k_2 + c_L^{(c)}}, \\
\frac{dc_L^{(n)}}{dt} &= r_1 c_L^{(c)} - r_2 c_L^{(n)} - \frac{m_3 c_L^{(n)}}{k_3 + c_L^{(n)}},
\end{aligned}
$$

$$
\begin{aligned}
\frac{dc_T^{(m)}}{dt} &= \frac{n_2 {c_Y^{(n)}}^b}{{g_2}^b + {c_Y^{(n)}}^b} \cdot \frac{{g_3}^c}{{g_3}^c + {c_L^{(n)}}^c} - \frac{m_4 c_T^{(m)}}{k_4 + c_T^{(m)}}, \\
\frac{dc_T^{(c)}}{dt} &= p_2 c_T^{(m)} - r_3 c_T^{(c)} + r_4 c_T^{(n)} - (m_5(1 - L_I(t)) + m_6)\, \frac{c_T^{(c)}}{k_5 + c_T^{(c)}}, \\
\frac{dc_T^{(n)}}{dt} &= r_3 c_T^{(c)} - r_4 c_T^{(n)} - (m_7(1 - L_I(t)) + m_8)\, \frac{c_T^{(n)}}{k_6 + c_T^{(n)}}, \\
\frac{dc_X^{(m)}}{dt} &= \frac{n_3 {c_T^{(n)}}^d}{{g_4}^d + {c_T^{(n)}}^d} - \frac{m_9 c_X^{(m)}}{k_7 + c_X^{(m)}}, \\
\frac{dc_X^{(c)}}{dt} &= p_3 c_X^{(m)} - r_5 c_X^{(c)} + r_6 c_X^{(n)} - \frac{m_{10} c_X^{(c)}}{k_8 + c_X^{(c)}}, \\
\frac{dc_X^{(n)}}{dt} &= r_5 c_X^{(c)} - r_6 c_X^{(n)} - \frac{m_{11} c_X^{(n)}}{k_9 + c_X^{(n)}}, \\
\frac{dc_Y^{(m)}}{dt} &= \frac{{g_6}^f}{{g_6}^f + {c_L^{(n)}}^f} \cdot \left(q_2 c_P^{(n)} L_I(t) + \frac{(n_4 L_I(t) + n_5)\, {g_5}^e}{{g_5}^e + {c_T^{(n)}}^e} \right) - \frac{m_{12} c_Y^{(m)}}{k_{10} + c_Y^{(m)}}, \\
\frac{dc_Y^{(c)}}{dt} &= p_4 c_Y^{(m)} - r_7 c_Y^{(c)} + r_8 c_Y^{(n)} - \frac{m_{13} c_Y^{(c)}}{k_{11} + c_Y^{(c)}}, \\
\frac{dc_Y^{(n)}}{dt} &= r_7 c_Y^{(c)} - r_8 c_Y^{(n)} - \frac{m_{14} c_Y^{(n)}}{k_{12} + c_Y^{(n)}}, \\
\frac{dc_P^{(n)}}{dt} &= p_5(1 - L_I(t)) - \frac{m_{15} c_P^{(n)}}{k_{13} + c_P^{(n)}} - q_3 c_P^{(n)} L_I(t), \\
\frac{dc_A^{(m)}}{dt} &= q_4 c_P^{(n)} L_I(t) + \frac{n_6 {c_L^{(n)}}^g}{{g_7}^g + {c_L^{(n)}}^g} - \frac{m_{16} c_A^{(m)}}{k_{14} + c_A^{(m)}}, \\
\frac{dc_A^{(c)}}{dt} &= p_6 c_A^{(m)} - r_9 c_A^{(c)} + r_{10} c_A^{(n)} - \frac{m_{17} c_A^{(c)}}{k_{15} + c_A^{(c)}}, \\
\frac{dc_A^{(n)}}{dt} &= r_9 c_A^{(c)} - r_{10} c_A^{(n)} - \frac{m_{18} c_A^{(n)}}{k_{16} + c_A^{(n)}}.
\end{aligned}
\tag{S1.3}
$$

1.4 JL2006S: extended S-System formulation

$$
\begin{aligned}
\frac{dc_P^{(n)}}{dt} &= -\beta_{1,1} c_P^{(n)} + \gamma_{1,1}(1 - L_I(t)) + \gamma_{1,2} c_P^{(n)} L_I(t), \\
\frac{dc_L^{(m)}}{dt} &= \alpha_2 {c_X^{(n)}}^{g_{2,1}} {c_A^{(n)}}^{g_{2,2}} - \beta_{2,1} c_L^{(m)} + \gamma_{2,1} c_P^{(n)} {c_A^{(n)}}^{g_{2,3}} L_I(t), \\
\frac{dc_L^{(c)}}{dt} &= \alpha_3 {c_L^{(m)}}^{g_{3,1}} - \beta_{3,1} c_L^{(c)}, \\
\frac{dc_L^{(n)}}{dt} &= \alpha_4 {c_L^{(c)}}^{g_{4,1}} - \beta_{4,1} c_L^{(n)}, \\
\frac{dc_T^{(m)}}{dt} &= \alpha_5 {c_L^{(n)}}^{g_{5,1}} {c_Y^{(n)}}^{g_{5,2}} - \beta_{5,1} c_T^{(m)},
\end{aligned}
$$

$$\begin{aligned}
\frac{dc_T^{(c)}}{dt} &= \alpha_6 {c_T^{(m)}}^{g_{6,1}} - \beta_{6,1} c_T^{(c)} + \gamma_{6,1} c_T^{(c)} (1 - L_I(t)),\\
\frac{dc_T^{(n)}}{dt} &= \alpha_7 {c_T^{(c)}}^{g_{7,1}} - \beta_{7,1} c_T^{(n)} + \gamma_{7,1} c_T^{(n)} (1 - L_I(t)),\\
\frac{dc_X^{(m)}}{dt} &= \alpha_8 {c_T^{(n)}}^{g_{8,1}} - \beta_{8,1} c_X^{(m)},\\
\frac{dc_X^{(c)}}{dt} &= \alpha_9 {c_X^{(m)}}^{g_{9,1}} - \beta_{9,1} c_X^{(c)},\\
\frac{dc_X^{(n)}}{dt} &= \alpha_{10} {c_X^{(c)}}^{g_{10,1}} - \beta_{10,1} c_X^{(n)},\\
\frac{dc_Y^{(m)}}{dt} &= \alpha_{11} {c_L^{(n)}}^{g_{11,1}} {c_T^{(n)}}^{g_{11,2}} - \beta_{11,1} c_Y^{(m)} + \gamma_{11,1} c_P^{(n)} {c_L^{(n)}}^{g_{11,3}} L_I(t) + \gamma_{11,2} {c_L^{(n)}}^{g_{11,4}} {c_T^{(n)}}^{g_{11,5}} L_I(t),\\
\frac{dc_Y^{(c)}}{dt} &= \alpha_{12} {c_Y^{(m)}}^{g_{12,1}} - \beta_{12,1} c_Y^{(c)},\\
\frac{dc_Y^{(n)}}{dt} &= \alpha_{13} {c_Y^{(c)}}^{g_{13,1}} - \beta_{13,1} c_Y^{(n)},\\
\frac{dc_A^{(m)}}{dt} &= \alpha_{14} {c_L^{(n)}}^{g_{14,1}} - \beta_{14,1} c_A^{(m)} + \gamma_{14,1} c_P^{(n)} L_I(t),\\
\frac{dc_A^{(c)}}{dt} &= \alpha_{15} {c_A^{(m)}}^{g_{15,1}} - \beta_{15,1} c_A^{(c)},\\
\frac{dc_A^{(n)}}{dt} &= \alpha_{16} {c_A^{(c)}}^{g_{16,1}} - \beta_{16,1} c_A^{(n)}.
\end{aligned} \tag{S1.4}$$

1.5 AP2012: original equations

$$\begin{aligned}
\frac{dc_L^{(m)}}{dt} &= q_1 L_I(t) c_P + \frac{n_1 {g_1}^a}{{g_1}^a + (c_{P9} + c_{P7} + c_{NI} + c_T)^a} - (m_1 L_I(t) + m_2(1 - L_I(t))) c_L^{(m)},\\
\frac{dc_L}{dt} &= (p_1 L_I(t) + p_2) c_L^{(m)} - m_3 c_L - \frac{p_3 {c_L}^c}{{g_2}^c + {c_L}^c},\\
\frac{dc_{Lmod}}{dt} &= \frac{p_3 {c_L}^c}{{g_3}^c + {c_L}^c} - m_4 c_{Lmod},\\
\frac{dc_P}{dt} &= p_7 (1 - L_I(t)) (1 - c_P) - m_{11} c_P L_I(t),\\
\frac{dc_{P9}^{(m)}}{dt} &= q_3 L_I(t) c_P + \frac{g_8}{g_8 + c_{EC}} \left(n_4 + \frac{n_7 {c_L}^e}{{g_9}^e + {c_L}^e} \right) - m_{12} c_{P9}^{(m)},\\
\frac{dc_{P9}}{dt} &= p_8 c_{P9}^{(m)} - (m_{13} + m_{22}(1 - L_I(t)) c_{P9},\\
\frac{dc_{P7}^{(m)}}{dt} &= \frac{n_8 {c_{Ltot}}^e}{{g_{10}}^e + {c_{Ltot}}^e} + \frac{n_9 {c_{P9}}^f}{{g_{11}}^f + {c_{P9}}^f} - m_{14} c_{P7}^{(m)},\\
\frac{dc_{P7}}{dt} &= p_9 c_{P7}^{(m)} - (m_{15} + m_{23}(1 - L_I(t)) c_{P7},\\
\frac{dc_{NI}^{(m)}}{dt} &= \frac{n_{10} {c_{Lmod}}^e}{{g_{12}}^e + {c_{Lmod}}^e} + \frac{n_{11} {c_{P7}}^b}{{g_{13}}^b + {c_{P7}}^b} - m_{16} c_{NI}^{(m)},\\
\frac{dc_{NI}}{dt} &= p_{10} c_{NI}^{(m)} - (m_{17} + m_{24}(1 - L_I(t)))\, c_{NI},
\end{aligned}$$

$$\frac{dc_T^{(m)}}{dt} = n_2 \frac{g_4}{g_4 + c_{EC}} \cdot \frac{{g_5}^e}{{g_5}^e + {c_L}^e} - m_5 c_T^{(m)},$$
$$\frac{dc_T}{dt} = p_4 c_T^{(m)} - (m_6 + m_7(1 - L_I(t))c_T(p_5 c_{ZTL} + c_{ZG}) - m_8 c_T,$$
$$\frac{dc_{E4}^{(m)}}{dt} = n_{13} \frac{g_2}{g_2 + c_{EC}} \cdot \frac{{g_6}^e}{{g_6}^e + {c_L}^e} - m_{34} c_{E4}^{(m)},$$
$$\frac{dc_{E4}}{dt} = p_{23} c_{E4}^{(m)} - m_{35} c_{E4} - p_{25} c_{E4} c_{E3n} + p_{21} c_{E34},$$
$$\frac{dc_{E3}^{(m)}}{dt} = n_3 \frac{{g_{16}}^e}{{g_{16}}^e + {c_L}^e} - m_{26} c_{E3}^{(m)},$$
$$\frac{dc_{E3c}}{dt} = p_{16} c_{E3}^{(m)} - m_9 c_{E3c} c_{COP1c} - p_{17} c_{E3c} c_{Gc} - p_{19} c_{E3c} + p_{20} c_{E3n},$$
$$\frac{dc_{E3n}}{dt} = p_{19} c_{E3c} - p_{20} c_{E3n} - p_{17} c_{E3n} c_{Gn} - m_{30} c_{E3n} c_{COP1d} - m_{29} c_{E3n} c_{COP1n} + p_{21} c_{E34} - p_{25} c_{E4} c_{E3n},$$
$$\frac{dc_{LUX}^{(m)}}{dt} = n_{13} \frac{g_2}{g_2 + c_{EC}} \cdot \frac{{g_6}^e}{{g_6}^e + {c_L}^e} - m_{34} c_{LUX}^{(m)},$$
$$\frac{dc_{LUX}}{dt} = p_{27} c_{LUX}^{(m)} - m_{39} c_{LUX} - p_{26} c_{LUX} c_{E34},$$
$$\frac{dc_{COP1c}}{dt} = n_5 - p_6 c_{COP1c} - m_{27} c_{COP1c}(1 + p_{15} L_I(t)),$$
$$\frac{dc_{COP1n}}{dt} = p_6 c_{COP1c} - n_6 L_I(t) c_P c_{COP1n} - n_{14} c_{COP1n} - m_{27} c_{COP1n}(1 + p_{15} L_I(t)),$$
$$\frac{dc_{COP1d}}{dt} = n_{14} c_{COP1n} + n_6 L_I(t) c_P c_{COP1n} - m_{31}(1 + m_{33}(1 - L_I(t)))c_{COP1d},$$
$$\frac{dc_{EGc}}{dt} = p_{17} c_{E3c} c_{Gc} - m_9 c_{EGc} c_{COP1c} - p_{18} c_{EGc} + p_{31} c_{EGn},$$
$$\frac{dc_{EC}}{dt} = p_{26} c_{LUX} c_{E34} - m_{36} c_{EC} c_{COP1n} - m_{37} c_{EC} c_{COP1d} - m_{32} c_{EC} \left(1 + p_{24} L_I(t) \frac{{c_{Gn\_tot}}^d}{{g_7}^d + {c_{Gn\_tot}}^d}\right),$$
$$\frac{dc_{ZTL}}{dt} = p_{14} - p_{12} L_I(t) c_{ZTL} c_{Gc} + p_{13} c_{ZG}(1 - L_I(t)) - m_{20} c_{ZTL},$$
$$\frac{dc_{ZG}}{dt} = p_{12} L_I(t) c_{ZTL} c_{Gc} - p_{13} c_{ZG}(1 - L_I(t)) - m_{21} c_{ZG},$$
$$\frac{dc_G^{(m)}}{dt} = q_2 L_I(t) c_P + n_{12} \frac{g_{14}}{g_{14} + c_{EC}} \cdot \frac{{g_{15}}^e}{{g_{15}}^e + {c_L}^e} - m_{18} c_G^{(m)},$$
$$\frac{dc_{Gc}}{dt} = p_{11} c_G^{(m)} - p_{12} L_I(t) c_{ZTL} c_{Gc} + p_{13} c_{ZG}(1 - L_I(t)) - m_{19} c_{Gc} - p_{17} c_{E3c} c_{Gc} - p_{28} c_{Gc} + p_{29} c_{Gn},$$

$$c_{Ltot} = c_L + c_{Lmod},$$
$$c_{E34} = \frac{p_{25} c_{E4} c_{E3n}}{p_{26} c_{LUX} + p_{21} + m_{37} c_{COP1d} + m_{36} c_{COP1n}},$$
$$c_{EGn} = \frac{p_{18} c_{EGc} + p_{17} c_{E3n} c_{Gn}}{m_9 c_{COP1n} + m_{10} c_{COP1d} + p_{31}},$$
$$c_{Gn} = \frac{p_{28} c_{Gc}}{p_{29} + m_{19} + p_{17} c_{E3n}},$$

$$c_{Gn\_tot} = c_{Gn} + c_{EGn}. \tag{S1.5}$$

1.6 AP2012S: extended S-System formulation

$$
\begin{aligned}
\frac{dc_L^{(m)}}{dt} &= \alpha_1(c_{P9} + c_{P7} + c_{NI} + c_T)^{g_{1,1}} - \beta_{1,1}c_L^{(m)} + \gamma_{1,1}c_P L_I(t) + \gamma_{1,2}c_L^{(m)} L_I(t),\\
\frac{dc_L}{dt} &= \alpha_2 c_L^{(m)} - \beta_{2,1}c_L + \gamma_{2,1}c_L^{(m)} L_I(t),\\
\frac{dc_{Lmod}}{dt} &= \alpha_3 {c_L}^{g_{3,1}} - \beta_{3,1}c_{Lmod},\\
\frac{dc_P}{dt} &= \gamma_{4,1}(1 - L_I(t)) + \gamma_{4,2}c_P(1 - L_I(t)) + \gamma_{4,3}c_P L_I(t),\\
\frac{dc_{P9}^{(m)}}{dt} &= \alpha_5 {c_L}^{g_{5,1}} {c_{EC}}^{g_{5,2}} - \beta_{5,1}c_{P9}^{(m)} + \gamma_{5,1}L_I(t)c_P,\\
\frac{dc_{P9}}{dt} &= \alpha_6 c_{P9}^{(m)} - \beta_{6,1}c_{P9} + \gamma_{6,1}c_{P9}(1 - L_I(t)),\\
\frac{dc_{P7}^{(m)}}{dt} &= \alpha_7(c_L + c_{Lmod})^{g_{7,1}} {c_{P9}}^{g_{7,2}} - \beta_{7,1}c_{P7}^{(m)},\\
\frac{dc_{P7}}{dt} &= \alpha_8 c_{P7}^{(m)} - \beta_{8,1}c_{P7} + \gamma_{8,1}c_{P7}(1 - L_I(t)),\\
\frac{dc_{NI}^{(m)}}{dt} &= \alpha_9 {c_{Lmod}}^{g_{9,1}} {c_{P7}}^{g_{9,2}} - \beta_{9,1}c_{NI}^{(m)},\\
\frac{dc_{NI}}{dt} &= \alpha_{10} c_{NI}^{(m)} - \beta_{10,1}c_{NI} + \gamma_{10,1}c_{NI}(1 - L_I(t)),\\
\frac{dc_T^{(m)}}{dt} &= \alpha_{11} {c_L}^{g_{11,1}} {c_{EC}}^{g_{11,2}} - \beta_{11,1}c_T^{(m)},\\
\frac{dc_T}{dt} &= \alpha_{12} c_T^{(m)} - \beta_{12,1}c_T - \beta_{12,2}c_{ZTL}c_T - \beta_{12,3}c_{ZG}c_T + \gamma_{12,1}c_{ZTL}c_T(1 - L_I(t))\\
&\quad + \gamma_{12,2}c_{ZG}c_T(1 - L_I(t)),\\
\frac{dc_{E4}^{(m)}}{dt} &= \alpha_{13} {c_L}^{g_{13,1}} {c_{EC}}^{g_{13,2}} - \beta_{13,1}c_{E4}^{(m)},\\
\frac{dc_{E4}}{dt} &= \alpha_{14} c_{E4}^{(m)} - \beta_{14,1}c_{E4} - \beta_{14,2}c_{E4}c_{E3}^{(n)}c_{LUX} - \beta_{14,3}c_{E4}c_{E3}^{(n)},\\
\frac{dc_{E3}^{(m)}}{dt} &= \alpha_{15} {c_L}^{g_{15,1}} - \beta_{15,1}c_{E3}^{(m)},\\
\frac{dc_{E3}^{(c)}}{dt} &= \alpha_{16} {c_{E3}^{(m)}}^{g_{16,1}} - \beta_{16,1}c_{E3}^{(c)} - \beta_{16,2}c_{E3}^{(c)}c_{COP1}^{(c)} - \beta_{16,3}c_{E3}^{(c)}c_{GI},\\
\frac{dc_{E3}^{(n)}}{dt} &= \alpha_{17} {c_{E3}^{(c)}}^{g_{17,1}} - \beta_{17,1}c_{E3}^{(n)} - \beta_{17,2}c_{E3}^{(n)}c_{GI} - \beta_{17,3}c_{E3}^{(n)}c_{COP1d} - \beta_{17,4}c_{E3}^{(n)}c_{COP1}^{(n)}\\
&\quad - \beta_{17,5}c_{E3}^{(n)}c_{E4}c_{LUX} - \beta_{17,6}c_{E3}^{(n)}c_{E4},\\
\frac{dc_{LUX}^{(m)}}{dt} &= \alpha_{18} {c_L}^{g_{18,1}} {c_{EC}}^{g_{18,2}} - \beta_{18,1}c_{LUX}^{(m)},\\
\frac{dc_{LUX}}{dt} &= \alpha_{19} c_{LUX}^{(m)} - \beta_{19,1}c_{LUX} - \beta_{19,2}c_{E3}^{(n)}c_{E4}c_{LUX},\\
\frac{dc_{COP1}^{(c)}}{dt} &= \alpha_{20} - \beta_{20,1}c_{COP1}^{(c)} + \gamma_{20,1}c_{COP1}^{(c)} L_I(t),
\end{aligned}
$$

$$\begin{aligned}
\frac{dc^{(n)}_{COP1}}{dt} &= \alpha_{21} {c^{(c)}_{COP1}}^{g_{21,1}} - \beta_{21,1} c^{(n)}_{COP1} + \gamma_{21,1} c^{(n)}_{COP1} c_P L_I(t) + \gamma_{21,2} c^{(n)}_{COP1} L_I(t),\\
\frac{dc_{COP1d}}{dt} &= \alpha_{22} c^{(n)}_{COP1} - \beta_{22,1} c_{COP1d} + \gamma_{22,1} c^{(n)}_{COP1} c_P L_I(t) + \gamma_{22,2} c_{COP1d}(1 - L_I(t)),\\
\frac{dc_{EG}}{dt} &= \alpha_{23} c^{(c)}_{E3} c_{GI} - \beta_{23,1} c_{EG} - \beta_{23,2} c_{EG} c^{(c)}_{COP1} - \beta_{23,3} c_{GI} c^{(n)}_{E3},\\
\frac{dc_{EC}}{dt} &= \alpha_{24} c^{(n)}_{E3} c_{E4} c_{LUX} - \beta_{24,1} c_{EC} - \beta_{24,2} c_{EC} c^{(n)}_{COP1} - \beta_{24,3} c_{EC} c_{COP1d} + \gamma_{24,1} c_{EC} c_{GI} L_I(t),\\
\frac{dc_{ZTL}}{dt} &= \alpha_{25} - \beta_{25,1} c_{ZTL} + \gamma_{25,1} c_{ZTL} c_{GI} L_I(t) + \gamma_{25,2} c_{ZG}(1 - L_I(t)),\\
\frac{dc_{ZG}}{dt} &= -\beta_{26,1} c_{ZG} + \gamma_{26,1} c_{GI} c_{ZTL} L_I(t) + \gamma_{26,2} c_{ZG}(1 - L_I(t)),\\
\frac{dc^{(m)}_{GI}}{dt} &= \alpha_{27} {c_L}^{g_{27,1}} {c_{EC}}^{g_{27,2}} - \beta_{27,1} c^{(m)}_{GI} + \gamma_{27,1} c_P L_I(t),\\
\frac{dc_{GI}}{dt} &= \alpha_{28} c^{(m)}_{GI} - \beta_{28,1} c_{GI} - \beta_{28,2} c_{GI} c^{(c)}_{E3} + \gamma_{28,1} c_{GI} c_{ZTL} L_I(t) - \gamma_{28,2} c_{ZG}(1 - L_I(t)). \qquad \text{(S1.6)}
\end{aligned}$$

1.7 KF2014: original equations

$$\begin{aligned}
\frac{dc^{(m)}_{L}}{dt} &= \frac{LC_{common}}{1 + (r_{11} LC)^2} - m_1 c^{(m)}_{L},\\
\frac{dc_{L}}{dt} &= (L_I(t) + m_4(1 - L_I(t))) c^{(m)}_{L} - m_3 c_L,\\
\frac{dc^{(m)}_{C}}{dt} &= LC_{common} - m_1 c^{(m)}_{C},\\
\frac{dc_{C}}{dt} &= (L_I(t) + m_4(1 -_I L_I(t)) c^{(m)}_{C} - m_3 c_C,\\
\frac{dc_{P}}{dt} &= p_7(1 - L_I(t))(1 - c_P) - m_{11} c_P L_I(t),\\
\frac{dc^{(m)}_{P9}}{dt} &= q_3 c_P L_I(t) - m_{12} c^{(m)}_{P9}\\
&\quad + \frac{1 + a_3 r_{33} c_R}{(1 + r_{33} c_R)\left(1 + (r_5 LC)^2\right)\left(1 + (r_6 EC)^2\right)\left(1 + \left(r_7 c^{(n)}_T\right)^2\right)\left(1 + \left(r_{40} c^{(n)}_{P5}\right)^2\right)},\\
\frac{dc_{P9}}{dt} &= c^{(m)}_{P9} - m_{13} c_{P9},\\
\frac{dc^{(m)}_{P7}}{dt} &= \frac{1}{\left(1 + (r_8 LC)^2\right)\left(1 + (r_9 EC)^2\right)\left(1 + \left(r_{10} c^{(n)}_T\right)^2\right)\left(1 + \left(r_{41} c^{(n)}_{P5}\right)^2\right)} - m_{14} c^{(m)}_{P7},\\
\frac{dc_{P7}}{dt} &= c^{(m)}_{P7} - (m_{15} + m_{23}(1 - L_I(t))) c_{P7},\\
\frac{dc^{(m)}_{P5}}{dt} &= \frac{1 + a_4 r_{34} c_R}{(1 + r_{34} c_R)\left(1 + (r_{12} LC)^2\right)\left(1 + (r_{13} EC)^2\right)\left(1 + \left(r_{14} c^{(n)}_T\right)^2\right)} - m_{16} c^{(m)}_{P5},\\
\frac{dc^{(c)}_{P5}}{dt} &= c^{(m)}_{P5} - (m_{17} + m_{24} c_{ZTL}) c^{(c)}_{P5} - P5_{trans},\\
\frac{dc^{(n)}_{P5}}{dt} &= P5_{trans} - m_{42} c^{(n)}_{P5},
\end{aligned}$$

$$\frac{dc_T^{(m)}}{dt} = \frac{1 + a_5 r_{35} c_R}{(1 + r_{35} c_R)\left(1 + (r_{15} LC)^2\right)\left(1 + (r_{16} EC)^2\right)\left(1 + \left(r_{17} c_T^{(n)}\right)^2\right)} - m_5 c_T^{(m)},$$

$$\frac{dc_T^{(c)}}{dt} = c_T^{(m)} - (m_8 + m_6 c_{ZTL}) c_T^{(c)} - T_{trans},$$

$$\frac{dc_T^{(n)}}{dt} = T_{trans} - \frac{m_{43}}{1 + m_{38} c_{P5}^{(n)}} c_T^{(n)},$$

$$\frac{dc_{E4}^{(m)}}{dt} = \frac{1 + a_6 r_{36} c_R}{(1 + r_{36} c_R)(1 + (r_{18} EC)^2)(1 + (r_{19} LC)^2)\left(1 + \left(r_{20} c_T^{(n)}\right)^2\right)} - m_7 c_{E4}^{(m)},$$

$$\frac{dc_{E4}}{dt} = p_{23} c_{E4}^{(m)} - m_{35} c_{E4} - {c_{E4}}^2,$$

$$\frac{dc_{E4d}}{dt} = {c_{E4}}^2 - m_{36} c_{E4d} - E34_{prod},$$

$$\frac{dc_{E3}^{(m)}}{dt} = \frac{1}{1 + (r_{21} LC)^2} - m_{26} c_{E3}^{(m)},$$

$$\frac{dc_{E3}}{dt} = p_{16} c_{E3}^{(m)} - E34_{prod} - E3_{deg} c_{E3},$$

$$\frac{dc_{E34}}{dt} = E34_{prod} - m_{22} c_{E34} E3_{deg},$$

$$\frac{dc_{LUX}^{(m)}}{dt} = \frac{1 + a_7 r_{37} c_R}{(1 + r_{37} c_R)\left(1 + (r_{22} EC)^2\right)\left(1 + (r_{23} LC)^2\right)\left(1 + \left(r_{24} c_T^{(n)}\right)^2\right)} - m_{34} c_{LUX}^{(m)},$$

$$\frac{dc_{LUX}}{dt} = c_{LUX}^{(m)} - m_{39} c_{LUX},$$

$$\frac{dc_{COP1}^{(c)}}{dt} = n_5 - p_6 c_{COP1}^{(c)} - m_{27}(1 + p_{15} L_I(t)) c_{COP1}^{(c)},$$

$$\frac{dc_{COP1}^{(n)}}{dt} = p_6 c_{COP1}^{(c)} - (n_{14} + n_6 L_I(t) c_P) c_{COP1}^{(n)} - m_{27}(1 + p_{15} L_I(t)) c_{COP1}^{(n)},$$

$$\frac{dc_{COP1d}}{dt} = (n_{14} + n_6 L_I(t) c_P) c_{COP1}^{(n)} - m_{31}(1 + m_{33}(1 - L_I(t))) c_{COP1d},$$

$$\frac{dc_{ZTL}}{dt} = p_{14} - ZG_{prod} - m_{20} c_{ZTL},$$

$$\frac{dc_{ZG}}{dt} = ZG_{prod} - m_{21} c_{ZG},$$

$$\frac{dc_{GI}^{(m)}}{dt} = \frac{1 + a_8 r_{38} c_R}{(1 + r_{38} c_R)\left(1 + (r_{25} EC)^2\right)\left(1 + (r_{26} LC)^2\right)\left(1 + \left(r_{27} c_T^{(n)}\right)^2\right)} - m_{18} c_G^{(m)},$$

$$\frac{dc_{GI}^{(c)}}{dt} = p_{11} c_G^{(m)} - ZG_{prod} - G_{trans} - m_{19} c_G^{(c)},$$

$$\frac{dc_{GI}^{(n)}}{dt} = G_{trans} - m_{19} c_G^{(n)} - m_{25} c_{E3tot}(1 + m_{28} c_{COP1d} + m_{32} c_{COP1n}) c_G^{(n)},$$

$$\frac{dc_{NOX}^{(m)}}{dt} = \frac{1}{\left(1 + (r_{28} LC)^2\right)\left(1 + (r_{29} c_{P7})^2\right)} - m_{44} c_{NOX}^{(m)},$$

$$
\begin{aligned}
\frac{dc_{NOX}}{dt} &= c_{NOX}^{(m)} - m_{45}c_{NOX}, \\
\frac{dc_R^{(m)}}{dt} &= \frac{1}{1 + (r_{30}c_{P9})^2 + (r_{31}c_{P7})^2 + \left(r_{32}c_{P5}^{(n)}\right)^2} - m_{46}c_R^{(m)}, \\
\frac{dc_R}{dt} &= c_R^{(m)} - m_{47}c_R,
\end{aligned}
$$

$$
\begin{aligned}
LC &= c_L + f_5c_C, \\
LC_{common} &= \frac{q_1L_I(t)c_P + 1}{1 + (r_1c_{P9})^2 + (r_2c_{P7})^2 + \left(r_3c_{P5}^{(n)}\right)^2 + \left(r_4c_T^{(n)}\right)^2}, \\
EC &= \frac{(c_{LUX} + f_6c_{NOX})(c_{E34} + f_1c_{E3})}{1 + f_3(c_{LUX} + f_2c_{NOX}) + f_4(c_{E34} + f_1c_{E3})}, \\
P5_{trans} &= t_5c_{P5}^{(c)} - t_6c_{P5}^{(n)}, \\
T_{trans} &= t_7c_T^{(c)} - \frac{t_8}{1 + m_{37}c_{P5}^{(n)}}c_T^{(n)}, \\
E34_{prod} &= p_{25}c_{E3}c_{E4d}, \\
E3_{deg} &= m_{30}c_{COP1d} + m_{29}c_{COP1n} + m_9 + m_{10}c_G^{(n)}, \\
ZG_{prod} &= p_{12}c_{ZTL}c_G^{(c)} - (p_{13}(1 - L_I(t)) + p_{10}L_I(t))c_{ZG}, \\
c_{E3tot} &= c_{E3} + c_{E34}, \\
G_{trans} &= p_{28}c_G^{(c)} - \frac{p_{29}}{1 + t_9c_{E3tot}}c_G^{(n)}. \qquad \text{(S1.7)}
\end{aligned}
$$

1.8 KF2014S: extended S-System formulation

$$
\begin{aligned}
\frac{dc_L^{(m)}}{dt} &= \alpha_1\left(c_{P9} + c_{P7} + c_{P5}^{(n)} + c_T^{(n)}\right)^{g_{1,1}} - \beta_{1,1}c_L^{(m)} + \gamma_{1,1}c_PL_I(t), \\
\frac{dc_L}{dt} &= \alpha_2c_L^{(m)} - \beta_{2,1}c_L + \gamma_{2,1}c_L^{(m)}L_I(t) \\
\frac{dc_C^{(m)}}{dt} &= \alpha_3\left(c_{P9} + c_{P7} + c_{P5}^{(n)} + c_T^{(n)}\right)^{g_{3,1}} - \beta_{3,1}c_C^{(m)} + \gamma_{3,1}c_PL_I(t), \\
\frac{dc_C}{dt} &= \alpha_4c_C^{(m)} - \beta_{4,1}c_C + \gamma_{4,1}c_C^{(m)}L_I(t), \\
\frac{dc_P}{dt} &= \gamma_{5,1}(1 - L_I(t)) + \gamma_{5,2}c_P(1 - L_I(t)) + \gamma_{5,3}c_PL_I(t), \\
\frac{dc_{P9}^{(m)}}{dt} &= \alpha_6{c_{R8}}^{g_{6,1}}(c_L + c_C)^{g_{6,2}}(c_{LUX} + c_{NOX})^{g_{6,3}}(c_{E3} + c_{E34})^{g_{6,4}}{c_T^{(n)}}^{g_{6,5}}{c_{P5}^{(n)}}^{g_{6,6}} \\
&\qquad - \beta_{6,1}c_{P9}^{(m)} + \gamma_{6,1}c_PL_I(t), \\
\frac{dc_{P9}}{dt} &= \alpha_7c_{P9}^{(m)} - \beta_{7,1}c_{P9}, \\
\frac{dc_{P7}^{(m)}}{dt} &= \alpha_8(c_L + c_C)^{g_{8,1}}(c_{LUX} + c_{NOX})^{g_{8,2}}(c_{E3} + c_{E34})^{g_{8,3}}{c_T^{(n)}}^{g_{8,4}}{c_{P5}^{(n)}}^{g_{8,5}} - \beta_{8,1}c_{P7}^{(m)}, \\
\frac{dc_{P7}}{dt} &= \alpha_9c_{P7}^{(m)} - \beta_{9,1}c_{P7} + \gamma_{9,1}c_{P7}(1 - L_I(t)), \\
\frac{dc_{P5}^{(m)}}{dt} &= \alpha_{10}{c_{R8}}^{g_{10,1}}(c_L + c_C)^{g_{10,2}}(c_{LUX} + c_{NOX})^{g_{10,3}}(c_{E3} + c_{E34})^{g_{10,4}}{c_T^{(n)}}^{g_{10,5}} - \beta_{10,1}c_{P5}^{(m)},
\end{aligned}
$$

$$\frac{dc_{P5}^{(c)}}{dt} = \alpha_{11} {c_{P5}^{(m)}}^{g_{11,1}} - \beta_{11,1} c_{P5}^{(c)} - \beta_{11,2} c_{ZTL} c_{P5}^{(c)},$$

$$\frac{dc_{P5}^{(n)}}{dt} = \alpha_{12} {c_{P5}^{(c)}}^{g_{12,1}} - \beta_{12,1} c_{P5}^{(n)},$$

$$\frac{dc_{T}^{(m)}}{dt} = \alpha_{13} {c_{R8}}^{g_{13,1}} \left(c_L + c_C\right)^{g_{13,2}} \left(c_{LUX} + c_{NOX}\right)^{g_{13,3}} \left(c_{E3} + c_{E34}\right)^{g_{13,4}} {c_T^{(n)}}^{g_{13,5}} - \beta_{13,1} c_T^{(m)},$$

$$\frac{dc_{T}^{(c)}}{dt} = \alpha_{14} {c_{T}^{(m)}}^{g_{14,1}} - \beta_{14,1} c_{T}^{(c)} - \beta_{14,2} c_{ZTL} c_{T}^{(c)},$$

$$\frac{dc_{T}^{(n)}}{dt} = \alpha_{15} {c_{T}^{(c)}}^{g_{15,1}} - \beta_{15,1} c_{T}^{(n)},$$

$$\frac{dc_{E4}^{(m)}}{dt} = \alpha_{16} {c_{R8}}^{g_{16,1}} \left(c_L + c_C\right)^{g_{16,2}} \left(c_{LUX} + c_{NOX}\right)^{g_{16,3}} \left(c_{E3} + c_{E34}\right)^{g_{16,4}} {c_T^{(n)}}^{g_{16,5}} - \beta_{16,1} c_{E4}^{(m)},$$

$$\frac{dc_{E4}}{dt} = \alpha_{17} c_{E4}^{(m)} - \beta_{17,1} c_{E4},$$

$$\frac{dc_{E4d}}{dt} = \alpha_{18} c_{E4} - \beta_{18,1} c_{E4d} - \beta_{18,2} c_{E3} c_{E4d},$$

$$\frac{dc_{E3}^{(m)}}{dt} = \alpha_{19} \left(c_L + c_C\right)^{g_{19,1}} - \beta_{19,2} c_{E3},$$

$$\frac{dc_{E3}}{dt} = \alpha_{20} c_{E3}^{(m)} - \beta_{20,1} c_{E3} - \beta_{20,2} c_{E3} c_{E4d} - \beta_{20,3} c_{E3} c_{COP1}^{(n)} - \beta_{20,4} c_{E3} c_{COP1d} - \beta_{20,5} c_{E3} c_{GI}^{(n)},$$

$$\frac{dc_{E34}}{dt} = \alpha_{21} c_{E3} c_{E4d} - \beta_{21,1} c_{E34} - \beta_{21,2} c_{E34} c_{COP1d} - \beta_{21,3} c_{E34} c_{COP1}^{(n)} - \beta_{21,4} c_{E34} c_{GI}^{(n)},$$

$$\frac{dc_{LUX}^{(m)}}{dt} = \alpha_{22} {c_{R8}}^{g_{22,1}} \left(c_L + c_C\right)^{g_{22,2}} \left(c_{LUX} + c_{NOX}\right)^{g_{22,3}} \left(c_{E3} + c_{E34}\right)^{g_{22,4}} {c_T^{(n)}}^{g_{22,5}} - \beta_{22,1} c_{LUX}^{(m)},$$

$$\frac{dc_{LUX}}{dt} = \alpha_{23} c_{LUX}^{(m)} - \beta_{23,1} c_{LUX},$$

$$\frac{dc_{COP1}^{(c)}}{dt} = \alpha_{24} - \beta_{24,1} c_{COP1}^{(c)} + \gamma_{24,1} c_{COP1}^{(c)} L_I(t),$$

$$\frac{dc_{COP1}^{(n)}}{dt} = \alpha_{25} {c_{COP1}^{(c)}}^{g_{25,1}} - \beta_{25,1} c_{COP1}^{(n)} + \gamma_{25,1} c_P c_{COP1}^{(n)} L_I(t) + \gamma_{25,2} c_{COP1}^{(n)} L_I(t),$$

$$\frac{dc_{COP1d}}{dt} = \alpha_{26} c_{COP1}^{(n)} - \beta_{26,1} c_{COP1d} + \gamma_{26,1} c_P c_{COP1}^{(n)} L_I(t) + \gamma_{26,2} c_{COP1d} (1 - L_I(t)),$$

$$\frac{dc_{ZTL}}{dt} = \alpha_{27} - \beta_{27,1} c_{ZTL} - \beta_{27,2} c_{ZTL} c_{GI}^{(c)} + \gamma_{27,1} c_{ZG} L_I(t) + \gamma_{27,2} c_{ZG} (1 - L_I(t)),$$

$$\frac{dc_{ZG}}{dt} = \alpha_{28} c_{ZTL} c_{GI}^{(c)} - \beta_{28,1} c_{ZG} + \gamma_{28,1} c_{ZG} L_I(t) + \gamma_{28,2} c_{ZG} (1 - L_I(t)),$$

$$\frac{dc_{GI}^{(m)}}{dt} = \alpha_{29} {c_{R8}}^{g_{29,1}} \left(c_L + c_C\right)^{g_{29,2}} \left(c_{LUX} + c_{NOX}\right)^{g_{29,3}} \left(c_{E3} + c_{E34}\right)^{g_{29,4}} {c_T^{(n)}}^{g_{29,5}} - \beta_{29,1} c_{GI}^{(m)},$$

$$\frac{dc_{GI}^{(c)}}{dt} = \alpha_{30} {c_{GI}^{(m)}}^{g_{30,1}} - \beta_{30,1} c_{GI}^{(c)} - \beta_{30,2} c_{ZTL} c_{GI}^{(c)} + \gamma_{30,1} c_{ZG} L_I(t) + \gamma_{30,2} c_{ZG} (1 - L_I(t)),$$

$$\begin{aligned}\frac{dc_{GI}^{(n)}}{dt} = \alpha_{31} {c_{GI}^{(c)}}^{g_{31,1}} - \beta_{31,1} c_{GI}^{(n)} - \beta_{31,2} (c_{E3} + c_{E34}) c_{GI}^{(n)} - \beta_{31,3} (c_{E3} + c_{E34}) c_{COP1}^{(n)} c_{GI}^{(n)} \\ - \beta_{31,4} (c_{E3} + c_{E34}) c_{COP1d} c_{GI}^{(n)},\end{aligned}$$

$$\frac{dc_{NOX}^{(m)}}{dt} = \alpha_{32} \left(c_L + c_C\right)^{g_{32,1}} {c_{P7}}^{g_{32,2}} - \beta_{32,1} c_{NOX}^{(m)},$$

$$\frac{dc_{NOX}}{dt} = \alpha_{33} c_{NOX}^{(m)} - \beta_{33,1} c_{NOX},$$

$$\frac{dc_{R8}^{(m)}}{dt} = \alpha_{34}\left(c_{P9} + c_{P7} + c_{P5}^{(n)}\right)^{g_{34,1}} - \beta_{34,1}c_{R8}^{(m)},$$
$$\frac{dc_{R8}}{dt} = \alpha_{35}c_{R8}^{(m)} - \beta_{35,1}c_{R8}. \tag{S1.8}$$

1.9 MF2016K: original equations

$$\frac{dc_P^{(p)}}{dt} = \phi_1 L_I(t) + \phi_2 c_P^{(p)} + \phi_3,$$
$$\frac{dc_L^{(m)}}{dt} = \frac{\phi_4}{{\phi_5}^2 + \phi_6\left(\phi_7 c_{P9}^{(p)} + \phi_8 c_{P7}^{(p)} + \phi_9 c_{P5}^{(p)}\right)^2} + \phi_{10}c_L^{(m)},$$
$$\frac{dc_L^{(p)}}{dt} = \phi_{11}c_L^{(m)} + \phi_{12}c_L^{(p)},$$
$$\frac{dc_{P9}^{(m)}}{dt} = \phi_{13}c_P^{(p)}L_I(t) + \phi_{14} + \frac{\phi_{15}{c_L^{(p)}}^2}{{\phi_{16}}^2 + {c_L^{(p)}}^2} + \phi_{17}c_{P9}^{(m)},$$
$$\frac{dc_{P9}^{(p)}}{dt} = \phi_{18}c_{P9}^{(m)} + \phi_{19}c_{P9}^{(p)},$$
$$\frac{dc_{P7}^{(m)}}{dt} = \frac{\phi_{20}}{\left(\phi_{21} + c_{EC}^{(p)}\right)\left({\phi_{22}}^2 + {c_T^{(p)}}^2\right)} + \phi_{23}c_{P7}^{(m)},$$
$$\frac{dc_{P7}^{(p)}}{dt} = \phi_{24}c_{P7}^{(m)} + \phi_{25}c_{P7}^{(p)},$$
$$\frac{dc_{P5}^{(m)}}{dt} = \frac{\phi_{26}}{\left({\phi_{27}}^2 + {c_T^{(p)}}^2\right)\left({\phi_{28}}^2 + {c_L^{(p)}}^2\right)} + \phi_{29}c_{P5}^{(m)},$$
$$\frac{dc_{P5}^{(p)}}{dt} = \phi_{30}c_{P5}^{(m)} + \phi_{31}c_{P5}^{(p)},$$
$$\frac{dc_T^{(m)}}{dt} = \frac{\phi_{32}}{\left(\phi_{33} + c_{EC}^{(p)}\right)\left({\phi_{34}}^2 + {c_L^{(p)}}^2\right)} + \phi_{35}c_T^{(m)},$$
$$\frac{dc_T^{(p)}}{dt} = \phi_{36}c_T^{(m)} + \phi_{37}c_T^{(p)},$$
$$\frac{dc_{EC}^{(p)}}{dt} = \phi_{38}c_{E3}^{(p)}c_{E4}^{(p)}c_{LUX}^{(p)} + \phi_{39}c_{EC}^{(p)},$$
$$\frac{dc_{R8}^{(m)}}{dt} = \frac{\phi_{40}}{{\phi_{41}}^2 + \phi_9{c_{P5}^{(p)}}^2} + \phi_{42}c_{R8}^{(m)},$$
$$\frac{dc_{R8}^{(p)}}{dt} = \phi_{43}c_{R8}^{(m)} + \phi_{44}c_{R8}^{(p)},$$
$$\frac{dc_{E3}^{(m)}}{dt} = \frac{\phi_{45}}{{\phi_{46}}^2 + {c_L^{(p)}}^2} + \phi_{47}c_{E3}^{(m)},$$
$$\frac{dc_{E3}^{(p)}}{dt} = \phi_{48}c_{E3}^{(m)} + \phi_{49}c_{E3}^{(p)},$$

$$
\begin{aligned}
\frac{dc_{E4}^{(m)}}{dt} &= \frac{\phi_{50}}{\left(\phi_{51}+c_{EC}^{(p)}\right)\left({\phi_{52}}^2+{c_L^{(p)}}^2\right)}+\phi_{53}c_{E4}^{(m)},\\
\frac{dc_{E4}^{(p)}}{dt} &= \phi_{54}c_{E4}^{(m)}+\phi_{55}c_{E4}^{(p)},\\
\frac{dc_{LUX}^{(m)}}{dt} &= \frac{\phi_{56}}{\left(\phi_{57}+c_{EC}^{(p)}\right)\left({\phi_{58}}^2+{c_L^{(p)}}^2\right)}+\phi_{59}c_{LUX}^{(m)},\\
\frac{dc_{LUX}^{(p)}}{dt} &= \phi_{60}c_{LUX}^{(m)}+\phi_{61}c_{LUX}^{(p)},\\
\frac{dc_{GI}^{(m)}}{dt} &= \frac{\phi_{62}}{\left({\phi_{63}}^2+{c_L^{(p)}}^2\right)\left({\phi_{64}}^2+{c_T^{(p)}}^2\right)\left({\phi_{65}}^2+{c_{E3}^{(p)}}^2\right)}+\phi_{66}c_{GI}^{(m)},\\
\frac{dc_{GI}^{(p)}}{dt} &= \phi_{67}c_{GI}^{(m)}+\phi_{68}c_{COP1}^{(p)}c_{E3}^{(p)}c_{GI}^{(p)}+\phi_{69}c_{GI}^{(p)},\\
\frac{dc_{COP1}^{(p)}}{dt} &= \phi_{70}L_I(t)+\phi_{71}c_{COP1}^{(p)}+\phi_{72},\\
\frac{dc_{ZTL}^{(p)}}{dt} &= \frac{\phi_{73}c_{ZTL}^{(p)}c_{GI}^{(p)}}{1+\theta_{144}c_{ZTL}^{(p)}+\theta_{145}c_{GI}^{(p)}}+\phi_{74}c_{ZTL}^{(p)}+\phi_{75}.
\end{aligned}
\tag{S1.9}
$$

1.10 MF2016KS: extended S-System formulation

$$
\begin{aligned}
\frac{dc_P}{dt} &= \alpha_1-\beta_{1,1}c_P+\gamma_{1,1}L_I(t),\\
\frac{dc_L^{(m)}}{dt} &= \alpha_2\left(c_{P9}+c_{P7}+c_{P5}\right)^{g_{2,1}}-\beta_{2,1}c_L^{(m)},\\
\frac{dc_L}{dt} &= \alpha_3c_L^{(m)}-\beta_{3,1}c_L,\\
\frac{dc_{P9}^{(m)}}{dt} &= \alpha_4{c_L}^{g_{4,1}}-\beta_{4,1}c_{P9}^{(m)}+\gamma_{4,1}c_PL_I(t),\\
\frac{dc_{P9}}{dt} &= \alpha_5c_{P9}^{(m)}-\beta_{5,1}c_{P9},\\
\frac{dc_{P7}^{(m)}}{dt} &= \alpha_6{c_T}^{g_{6,1}}{c_{EC}}^{g_{6,2}}-\beta_{6,1}c_{P7}^{(m)},\\
\frac{dc_{P7}}{dt} &= \alpha_7c_{P7}^{(m)}-\beta_{7,1}c_{P7},\\
\frac{dc_{P5}^{(m)}}{dt} &= \alpha_8{c_L}^{g_{8,1}}{c_T}^{g_{8,2}}-\beta_{8,1}c_{P5}^{(m)},\\
\frac{dc_{P5}}{dt} &= \alpha_9c_{P5}^{(m)}-\beta_{9,1}c_{P5},\\
\frac{dc_T^{(m)}}{dt} &= \alpha_{10}{c_L}^{g_{10,1}}{c_{EC}}^{g_{10,2}}-\beta_{10,1}c_T^{(m)},\\
\frac{dc_T}{dt} &= \alpha_{11}c_T^{(m)}-\beta_{11,1}c_T,\\
\frac{dc_{EC}}{dt} &= \alpha_{12}c_{E3}c_{E4}c_{LUX}-\beta_{12,1}c_{EC},\\
\frac{dc_{R8}^{(m)}}{dt} &= \alpha_{13}{c_{P5}}^{g_{13,1}}-\beta_{13,1}c_{R8},
\end{aligned}
$$

$$
\begin{aligned}
\frac{dc_{R8}}{dt} &= \alpha_{14} c_{R8}^{(m)} - \beta_{14,1} c_{R8}, \\
\frac{dc_{E3}^{(m)}}{dt} &= \alpha_{15} {c_L}^{g_{15,1}} - \beta_{15,1} c_{E3}^{(m)}, \\
\frac{dc_{E3}}{dt} &= \alpha_{16} c_{E3}^{(m)} - \beta_{16,1} c_{E3}, \\
\frac{dc_{E4}^{(m)}}{dt} &= \alpha_{17} {c_L}^{g_{17,1}} {c_{EC}}^{g_{17,2}} - \beta_{17,1} c_{E4}^{(m)}, \\
\frac{dc_{E4}}{dt} &= \alpha_{18} c_{E4}^{(m)} - \beta_{18,1} c_{E4}, \\
\frac{dc_{LUX}^{(m)}}{dt} &= \alpha_{19} {c_L}^{g_{19,1}} {c_{EC}}^{g_{19,2}} - \beta_{19,1} c_{LUX}^{(m)}, \\
\frac{dc_{LUX}}{dt} &= \alpha_{20} c_{LUX}^{(m)} - \beta_{20,1} c_{LUX}, \\
\frac{dc_{GI}^{(m)}}{dt} &= \alpha_{21} {c_L}^{g_{21,1}} {c_T}^{g_{21,2}} {c_{E3}}^{g_{21,3}} - \beta_{21,1} c_{GI}^{(m)}, \\
\frac{dc_{GI}}{dt} &= \alpha_{22} c_{GI}^{(m)} - \beta_{22,1} c_{GI} - \beta_{22,2} c_{GI} c_{E3} c_{COP1}, \\
\frac{dc_{COP1}}{dt} &= \alpha_{23} - \beta_{23,1} c_{COP1} + \gamma_{23,1} L_I(t), \\
\frac{dc_{ZTL}}{dt} &= \alpha_{24} - \beta_{24,1} c_{ZTL} - \beta_{24,2} {c_{ZTL}}^{h_{24,1}} {c_{GI}}^{h_{24,2}}. \qquad \text{(S1.10)}
\end{aligned}
$$

Note: For the ZTL protein equation, we have included exponents $h_{i,k}$ controlling the stabilisation of ZTL by GI protein. This is because in MF2016K, this process was modelled as a rational function of the two protein concentrations (*cf.* eqs. (S1.9)), rather than as a product of the concentrations, as is usually the case (*cf.* eq. (13)).

1.11 MF2016KSorig: original S-System formulation

$$
\begin{aligned}
\frac{dc_P}{dt} &= \alpha_1 - \beta_{1,1} {L_I(t)}^{h_{1,1}} {c_P}^{h_{1,2}}, \\
\frac{dc_L^{(m)}}{dt} &= \alpha_2 {c_{P9}}^{g_{2,1}} {c_{P7}}^{g_{2,2}} {c_{P5}}^{g_{2,3}} - \beta_{2,1} c_L^{(m)}, \\
\frac{dc_L}{dt} &= \alpha_3 c_L^{(m)} - \beta_{3,1} c_L, \\
\frac{dc_{P9}^{(m)}}{dt} &= \alpha_4 {c_L}^{g_{4,1}} {c_P}^{g_{4,2}} {L_I(t)}^{g_{4,3}} - \beta_{4,1} c_{P9}^{(m)}, \\
\frac{dc_{P9}}{dt} &= \alpha_5 c_{P9}^{(m)} - \beta_{5,1} c_{P9}, \\
\frac{dc_{P7}^{(m)}}{dt} &= \alpha_6 {c_T}^{g_{6,1}} {c_{EC}}^{g_{6,2}} - \beta_{6,1} c_{P7}^{(m)}, \\
\frac{dc_{P7}}{dt} &= \alpha_7 c_{P7}^{(m)} - \beta_{7,1} c_{P7}, \\
\frac{dc_{P5}^{(m)}}{dt} &= \alpha_8 {c_L}^{g_{8,1}} {c_T}^{g_{8,2}} - \beta_{8,1} c_{P5}^{(m)}, \\
\frac{dc_{P5}}{dt} &= \alpha_9 c_{P5}^{(m)} - \beta_{9,1} c_{P5}, \\
\frac{dc_T^{(m)}}{dt} &= \alpha_{10} {c_L}^{g_{10,1}} {c_{EC}}^{g_{10,2}} - \beta_{10,1} c_T^{(m)},
\end{aligned}
$$

$$
\begin{aligned}
\frac{dc_T}{dt} &= \alpha_{11} c_T^{(m)} - \beta_{11,1} c_T, \\
\frac{dc_{EC}}{dt} &= \alpha_{12} c_{E3} c_{E4} c_{LUX} - \beta_{12,1} c_{EC}, \\
\frac{dc_{R8}^{(m)}}{dt} &= \alpha_{13} {c_{P5}}^{g_{13,1}} - \beta_{13,1} c_{R8}, \\
\frac{dc_{R8}}{dt} &= \alpha_{14} c_{R8}^{(m)} - \beta_{14,1} c_{R8}, \\
\frac{dc_{E3}^{(m)}}{dt} &= \alpha_{15} {c_L}^{g_{15,1}} - \beta_{15,1} c_{E3}^{(m)}, \\
\frac{dc_{E3}}{dt} &= \alpha_{16} c_{E3}^{(m)} - \beta_{16,1} c_{E3}, \\
\frac{dc_{E4}^{(m)}}{dt} &= \alpha_{17} {c_L}^{g_{17,1}} {c_{EC}}^{g_{17,2}} - \beta_{17,1} c_{E4}^{(m)}, \\
\frac{dc_{E4}}{dt} &= \alpha_{18} c_{E4}^{(m)} - \beta_{18,1} c_{E4}, \\
\frac{dc_{LUX}^{(m)}}{dt} &= \alpha_{19} {c_L}^{g_{19,1}} {c_{EC}}^{g_{19,2}} - \beta_{19,1} c_{LUX}^{(m)}, \\
\frac{dc_{LUX}}{dt} &= \alpha_{20} c_{LUX}^{(m)} - \beta_{20,1} c_{LUX}, \\
\frac{dc_{GI}^{(m)}}{dt} &= \alpha_{21} {c_L}^{g_{21,1}} {c_T}^{g_{21,2}} {c_{E3}}^{g_{21,3}} - \beta_{21,1} c_{GI}^{(m)}, \\
\frac{dc_{GI}}{dt} &= \alpha_{22} c_{GI}^{(m)} - \beta_{22,1} {c_{GI}}^{h_{22,1}} {c_{E3}}^{h_{22,2}} {c_{COP1}}^{h_{22,3}}, \\
\frac{dc_{COP1}}{dt} &= \alpha_{23} - \beta_{23,1} {L_I(t)}^{h_{23,1}} {c_{COP1}}^{h_{23,2}}, \\
\frac{dc_{ZTL}}{dt} &= \alpha_{24} {c_{ZTL}}^{g_{24,1}} {c_{GI}}^{g_{24,2}} - \beta_{24,1} c_{ZTL}.
\end{aligned}
\tag{S1.11}
$$

2 The sine-sweeping method

In sine-sweeping, the input to the system is a sinusoidal signal whose frequency is varied within the range of interest. By collecting the magnitude and phase values of the output responses, the transfer function of the system can be approximated. Here, a summary of the method is given. For more details see [S2].

Let the sinusoidal input signal be $x(t) = U_{in} \sin(\omega t)$, where U_{in} and ω are the amplitude and frequency respectively. According to linear systems theory, if the system is linear time invariant, the output response will be a sinusoid of the same frequency, but with scaled amplitude and a phase shift. In practice, nonlinearities, transients, and disturbances $V(t)$ will also affect the output. Thus, we can write the output $y(t)$ as

$$y(t) = U_{out} \sin(\omega t + \Phi) + V(t) + \text{nonlinearities} + \text{transients}, \tag{S2.12}$$

where $U_{out} = U_{in}|G(i\omega)|$, $\Phi = \angle G(i\omega) = \arctan\left(\frac{\Im|G(i\omega)|}{\Re|G(i\omega)|}\right)$ and $G(i\omega)$ is the transfer function relating the input and output.

By neglecting the initial part of the data and assuming that the linear dynamics dominate, we can ignore the effects of transients and nonlinearities, respectively. Furthermore, the effect of disturbances $V(t)$ can be reduced through the correlation method [S2], where the idea is to correlate the output response $y(t)$ with a sine and cosine of the same frequency, and then average over the signal length (**S9 Fig**). Writing the sampled output signal as $\{y(t_k) : 1 \leq k \leq L\}$, the resulting

averages are given below:

$$Q_S(L) = \frac{1}{L}\sum_{k=1}^{L} y(t_k)\sin(\omega t_k), \quad Q_C(L) = \frac{1}{L}\sum_{k=1}^{L} y(t_k)\cos(\omega t_k). \tag{S2.13}$$

By substituting (S2.12) into (S2.13) and performing some algebraic manipulation, we obtain

$$\begin{aligned} Q_S(L) &= \frac{U_{in}}{2}|G(i\omega)|\cos\Phi - \frac{U_i}{2}|G(i\omega)|\frac{1}{L}\sum_{k=1}^{L}\cos(2\omega t_k + \Phi) + \frac{1}{L}\sum_{k=1}^{L} V(t_k)\sin(\omega t_k), \\ Q_C(L) &= \frac{U_{in}}{2}|G(i\omega)|\sin\Phi - \frac{U_{in}}{2}|G(j\omega)|\frac{1}{L}\sum_{k=1}^{L}\sin(2\omega t_k + \Phi) + \frac{1}{L}\sum_{k=1}^{L} V(t_k)\cos(\omega t_k). \end{aligned} \tag{S2.14}$$

The second terms in the expressions for $Q_S(L)$ and $Q_C(L)$ converge to zero as $L \to \infty$. Furthermore, if $V(t)$ is a zero-mean stationary stochastic process with covariance function $R_c(\tau)$ such that $\sum_{\tau=0}^{\infty} \tau|R_c(\tau)| < \infty$, then it can be shown that the third terms in the expressions for $Q_S(L)$ and $Q_C(L)$ also converge to zero as $L \to \infty$ (see [S2] for complete details). From the remaining terms in (S2.14), the magnitude, $|G(i\omega)|$, and phase, $\angle G(i\omega)$, can therefore be estimated using the equations below:

$$|G(i\omega)| = \frac{2\sqrt{Q_S(L)^2 + Q_C(L)^2}}{U_{in}}, \quad \angle G(i\omega) = \arctan\left(\frac{Q_C(L)}{Q_S(L)}\right). \tag{S2.15}$$

Plotting $|G(i\omega)|$ and $\angle G(i\omega)$ against frequency ω then yields the Bode plot representation of the sine-sweeping approximation to the transfer function $G(i\omega)$.

3 Transfer function analysis of protein shuttling in JL2006

The Bode plots for JL2006 generated using sine-sweeping that are shown in Fig 6 of the main paper imply that the linear systems approximations to the functions relating input mRNAs to output nuclear proteins can be accurately represented as first-order transfer functions. In this section, we provide further evidence for the viability of this approximation using frequency response analysis, and also show that a first-order transfer function is equivalent to having an aggregated protein equation, rather than two separate equations modelling protein shuttling.

We start by noting that the protein shuttling mechanisms used in JL2006 all have the general form shown below (*cf.* eqs. (S1.3)):

$$\begin{aligned} \frac{dP_{j,cy}}{dt} &= \alpha G_i - \beta_{cy}P_{j,cy} + \beta_{nu}P_{j,nu} - \gamma_{cy}\frac{P_{j,cy}}{K_{cy} + P_{j,cy}}, \\ \frac{dP_{j,nu}}{dt} &= \beta_{cy}P_{j,cy} - \beta_{nu}P_{j,nu} - \gamma_{nu}\frac{P_{j,nu}}{K_{nu} + P_{j,cy}}. \end{aligned} \tag{S3.1}$$

Here, G_i is the gene, $P_{j,cy}$ is cytoplasmic protein, $P_{j,nu}$ is nuclear protein, α is the translation rate, $\{\gamma_{cy}, \gamma_{nu}\}$ are degradation rates, $\{K_{cy}, K_{nu}\}$ are Michaelis constants for degradation and $\{\beta_{cy}, \beta_{nu}\}$ are transport rates. Linearising the nonlinear degradation terms in eqs. (S3.1) and rescaling the degradation rates yields the following:

$$\begin{aligned} \frac{dP_{j,cy}}{dt} &= \alpha G_i - \beta_{cy}P_{j,cy} + \beta_{nu}P_{j,nu} - \gamma_{cy}P_{j,cy}, \\ \frac{dP_{j,nu}}{dt} &= \beta_{cy}P_{j,cy} - \beta_{nu}P_{j,nu} - \gamma_{nu}P_{j,nu}. \end{aligned} \tag{S3.2}$$

Taking Laplace transforms of (S3.1) and assuming zero initial conditions, we arrive at the following equations involving the transforms of $G_i(t)$, $P_{j,cy}(t)$ and $P_{j,nu}(t)$:

$$\begin{aligned} P_{j,cy}(s) &= \frac{\alpha}{s+\beta_{cy}+\gamma_{cy}} G_i(s) + \frac{\beta_{nu}}{s+\beta_{cy}+\gamma_{cy}} P_{j,nu}(s), \\ P_{j,nu}(s) &= \frac{\beta_{cy}}{s+\beta_{nu}+\gamma_{nu}} P_{j,cy}(s). \end{aligned} \tag{S3.3}$$

Some algebraic manipulation then shows that the transfer function relating input mRNA ($G_i(s)$) to output nuclear protein ($P_{j,nu}(s)$) is given by:

$$\frac{P_{j,nu}(s)}{G_i(s)} = \frac{\alpha\beta_{cy}}{s^2 + (\beta_{cy}+\gamma_{cy}+\beta_{nu}+\gamma_{nu})s + (\beta_{nu}\gamma_{cy}+\beta_{cy}\gamma_{nu}+\gamma_{cy}\gamma_{nu})}. \tag{S3.4}$$

We note that eq. (S3.4) is a second order-transfer function. The only way in which eq. (S3.4) can be approximated by a first-order transfer function, as suggested by the sine-sweeping results, is if its two poles (the roots of its denominator) are far apart; in other words, if eq. (S3.4) has one fast pole and one slow pole.

We demonstrate that is indeed the case for component Y of JL2006, and show the similarity between the Bode plots for the full second-order transfer function and its first-order approximation (similar results can be demonstrated for the other components of the model). In order to compute the full second-order transfer function of Y, we take the values $\alpha = p_4 = 0.2485$, $\beta_{cy} = r_7 = 2.2123$ and $\beta_{nu} = r_8 = 0.2002$ from **S5 Table**. For the nonlinear degradation terms, we use the best linear approximations (see **S10 Fig**), yielding the values $\gamma_{cy} = 0.073$ and $\gamma_{nu} = 0.324$. Substituting all these values into eq. (S3.4) gives

$$\frac{P_{j,nu}(s)}{G_i(s)} = \frac{0.04975}{s^2 + 2.809s + 0.25} = \frac{0.04975}{(s+0.092)(s+2.718)}. \tag{S3.5}$$

Note that the two poles differ in value by almost a factor of 30; there is thus one fast pole and one slow pole, as predicted. The Bode plot for eq. (S3.5), plotted over the same frequency range as that used for sine-sweeping, is shown as blue lines in **S11 Fig**.

We now consider the equation for an aggregated model of nuclear protein production (*cf.* eq. (10) of the main paper):

$$\frac{dP_{j,nu}}{dt} = aG_i - bP_{j,nu}. \tag{S3.6}$$

Here, a and b represent translation and degradation, respectively. Taking Laplace transforms and assuming zero initial conditions as before results in the following first-order transfer function:

$$\frac{P_{j,nu}(s)}{G_i(s)} = \frac{a}{s+b}. \tag{S3.7}$$

The values of a and b in eq. (S3.7) can be inferred from a Bode plot by using the fact that b is the corner frequency – the frequency at which the magnitude trace starts to deviate from its plateau value $A_{vdB} = 20\log_{10}(a/b)$. The Bode plot for eq. (S3.5) implies $b \approx 0.090$ and $A_{vdB} = -14$ dB, from which it follows that $a \approx 0.018$. Comparing Bode plots in **S11 Fig** shows that the full second-order transfer function for Y (eq. (S3.5)) is quite accurately modelled by the first-order approximation (eq. (S3.7)) with these values of a and b, particularly in the lower frequency range.

References

[S1] Locke JCW, Millar AJ, Turner MS. Modelling genetic networks with noisy and varied experimental data: the circadian clock in *Arabidopsis thaliana*. J Theor Biol. 2005;234:383–393. doi:10.1016/j.jtbi.2004.11.038.

[S2] Ljung L. System Identification: Theory for the User. 2nd ed. Prentice Hall; 1999.

Supplementary Figures

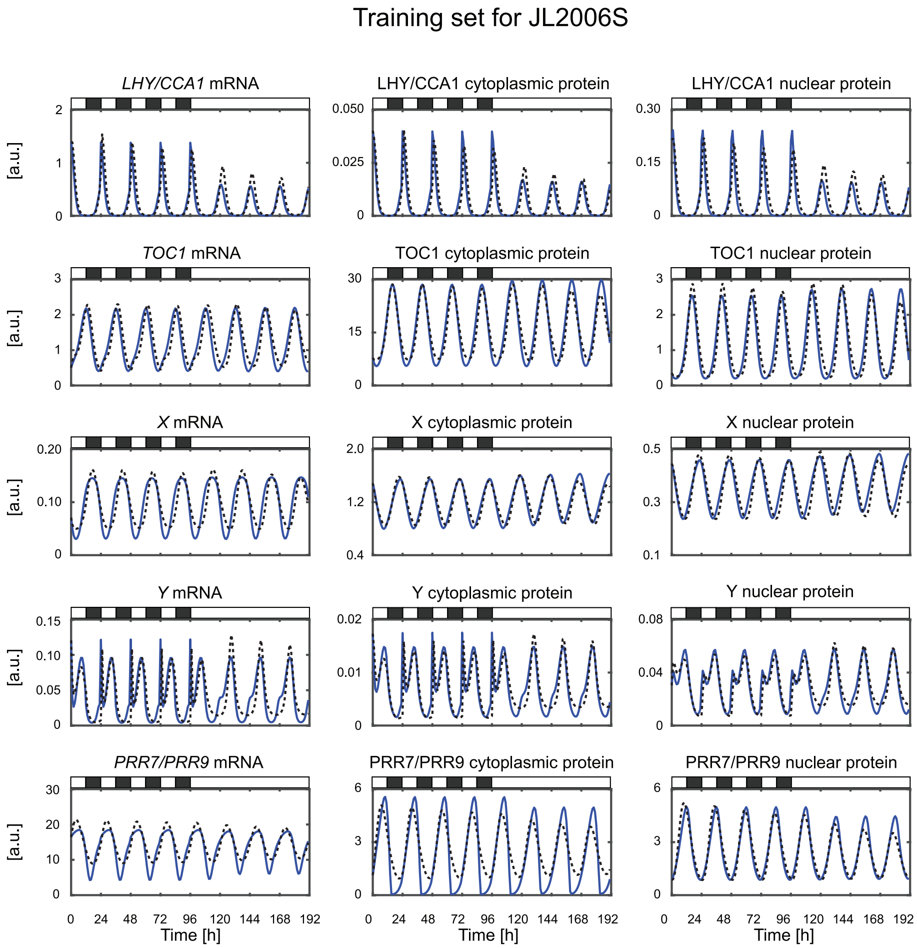

S1 Fig. Plant clock model JL2006S – optimal fits to synthetic training data. Blue solid lines show timeseries generated by JL2006 from its nominal parameter values for a simulated transition between a 12L:12D light-dark cycle and constant light (LL). Black dashed lines show timeseries obtained by optimising the parameters of JL2006S to this data in the same simulated light environment (see **Fig 2B** for the corresponding heatmaps). White and black bars at the top of the figure indicate light and dark intervals, respectively.

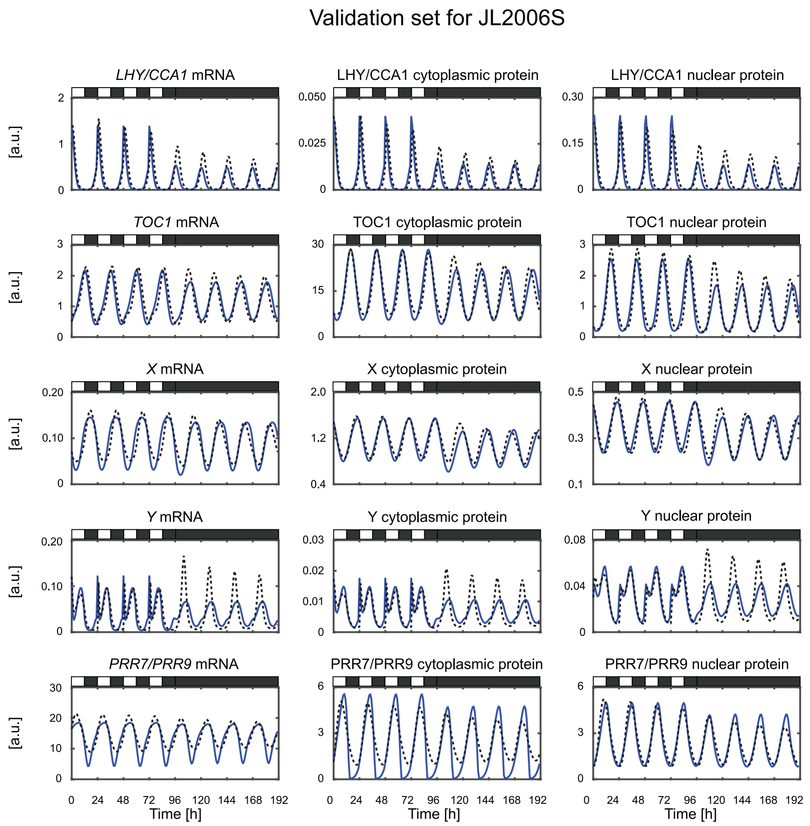

S2 Fig. Plant clock model JL2006S – fits to synthetic validation data. Blue solid lines show timeseries generated by JL2006 from its nominal parameter values for a simulated transition between a 12L:12D light-dark cycle and constant dark (DD). Black dashed lines show timeseries generated by JL2006S in the same simulated light environment using the parameters optimised to the training data (see **Fig 2C** for the corresponding heatmaps). White and black bars at the top of the figure indicate light and dark intervals, respectively.

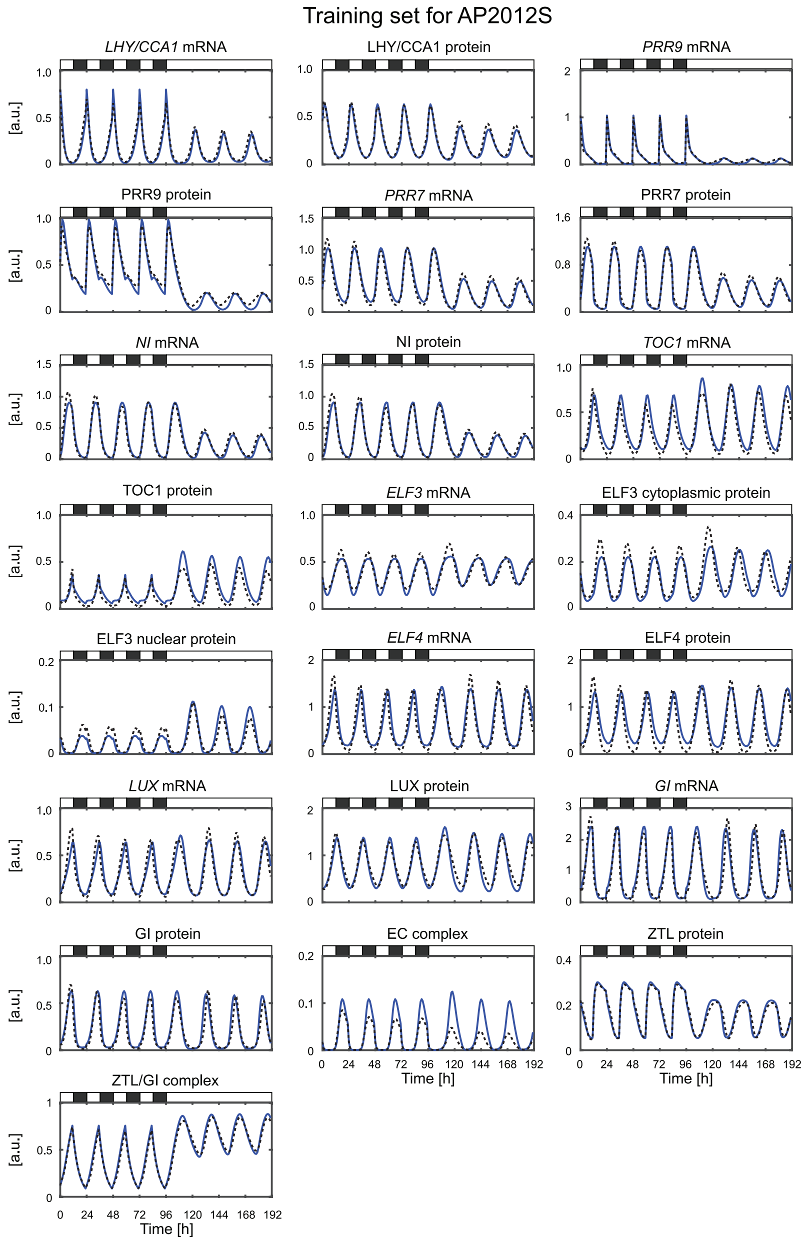

S3 Fig. Plant clock model AP2012S – optimal fits to synthetic training data. Blue solid lines show timeseries generated by AP2012 from its nominal parameter values for a simulated transition between a 12L:12D light-dark cycle and constant light (LL). Black dashed lines show timeseries obtained by optimising the parameters of AP2012S to this data in the same simulated light environment (see **Fig 3B** for the corresponding heatmaps). White and black bars at the top of the figure indicate light and dark intervals, respectively.

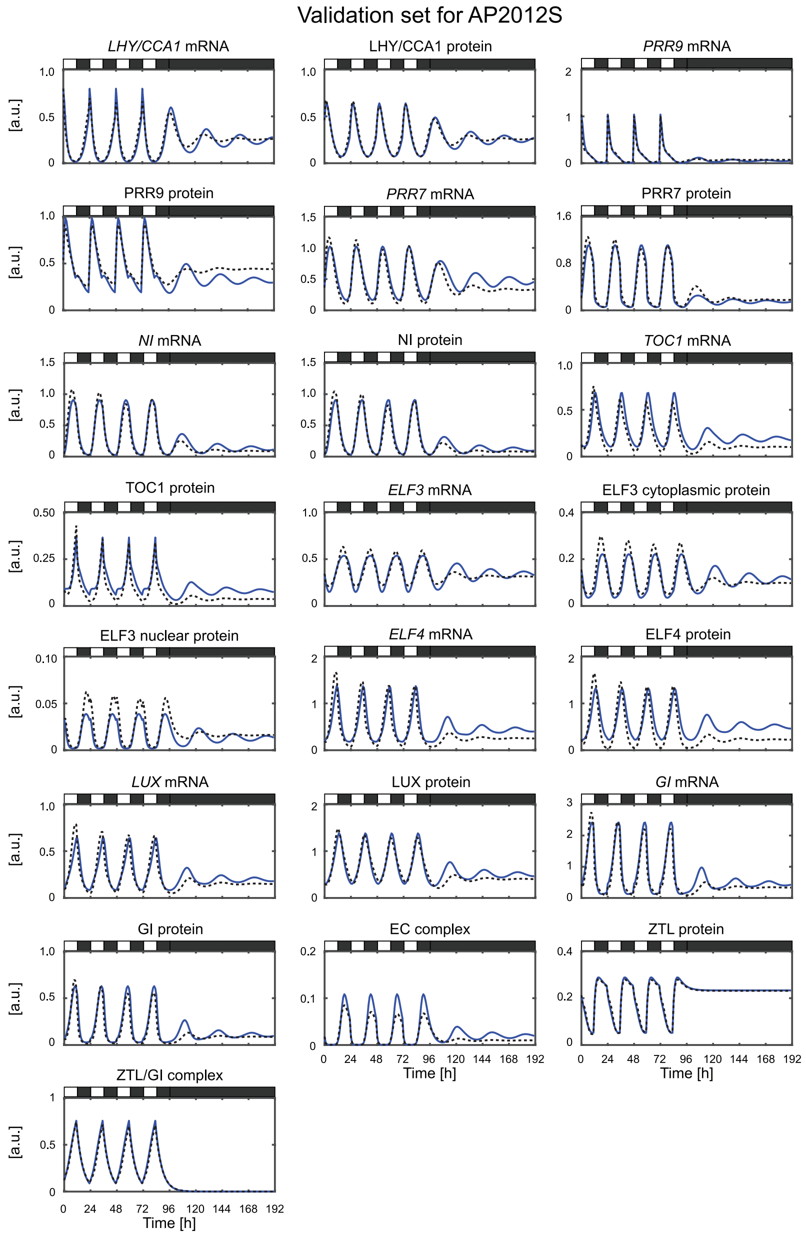

S4 Fig. Plant clock model AP2012S – fits to synthetic validation data. Blue solid lines show timeseries generated by AP2012 from its nominal parameter values for a simulated transition between a 12L:12D light-dark cycle and constant dark (DD). Black dashed lines show timeseries generated by AP2012S in the same simulated light environment using the parameters optimised to the training data (see **Fig 3C** for the corresponding heatmaps). White and black bars at the top of the figure indicate light and dark intervals, respectively.

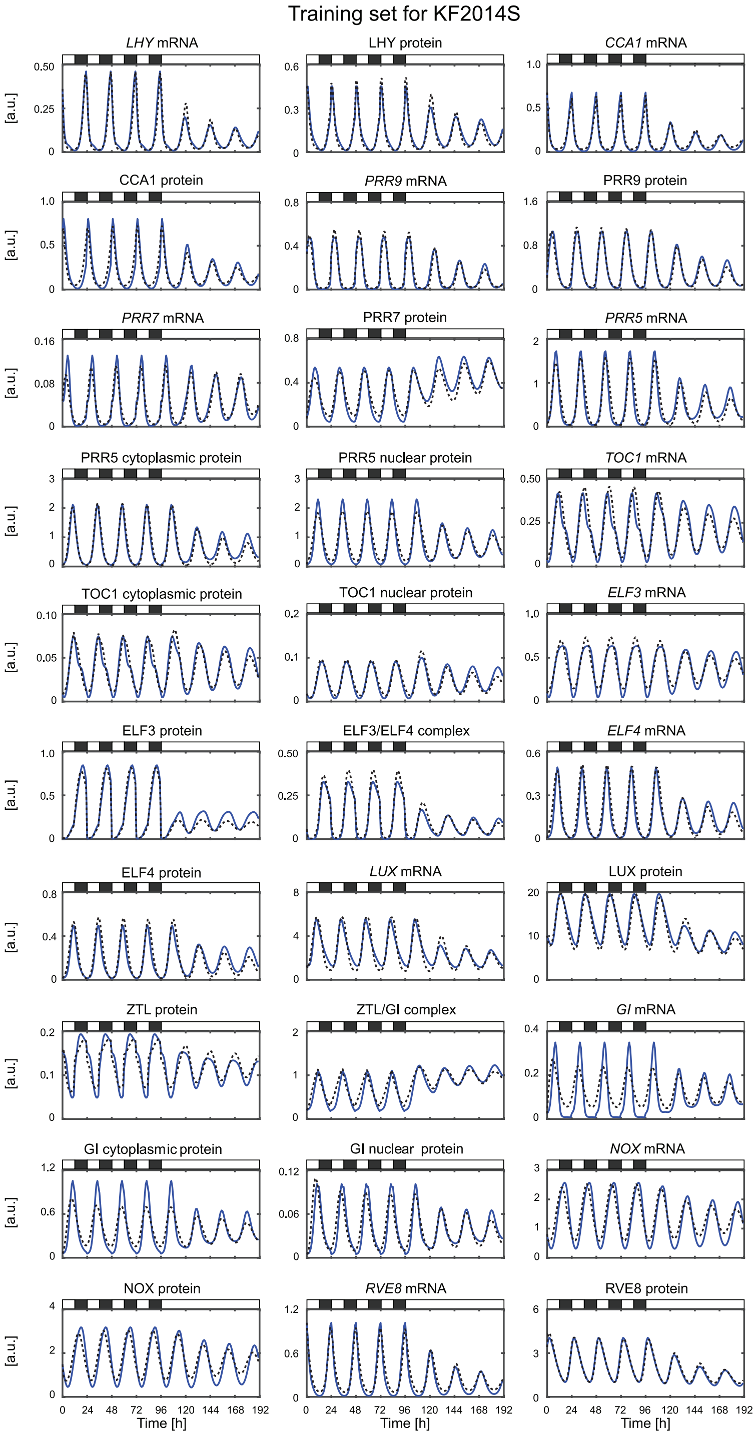

S5 Fig. Plant clock model KF2014S – optimal fits to synthetic training data. Blue solid lines show timeseries generated by KF2014 from its nominal parameter values for a simulated transition between a 12L:12D light-dark cycle and constant light (LL). Black dashed lines show timeseries obtained by optimising the parameters of KF2014S to this data in the same simulated light environment (see **Fig 4B** for the corresponding heatmaps). White and black bars at the top of the figure indicate light and dark intervals, respectively.

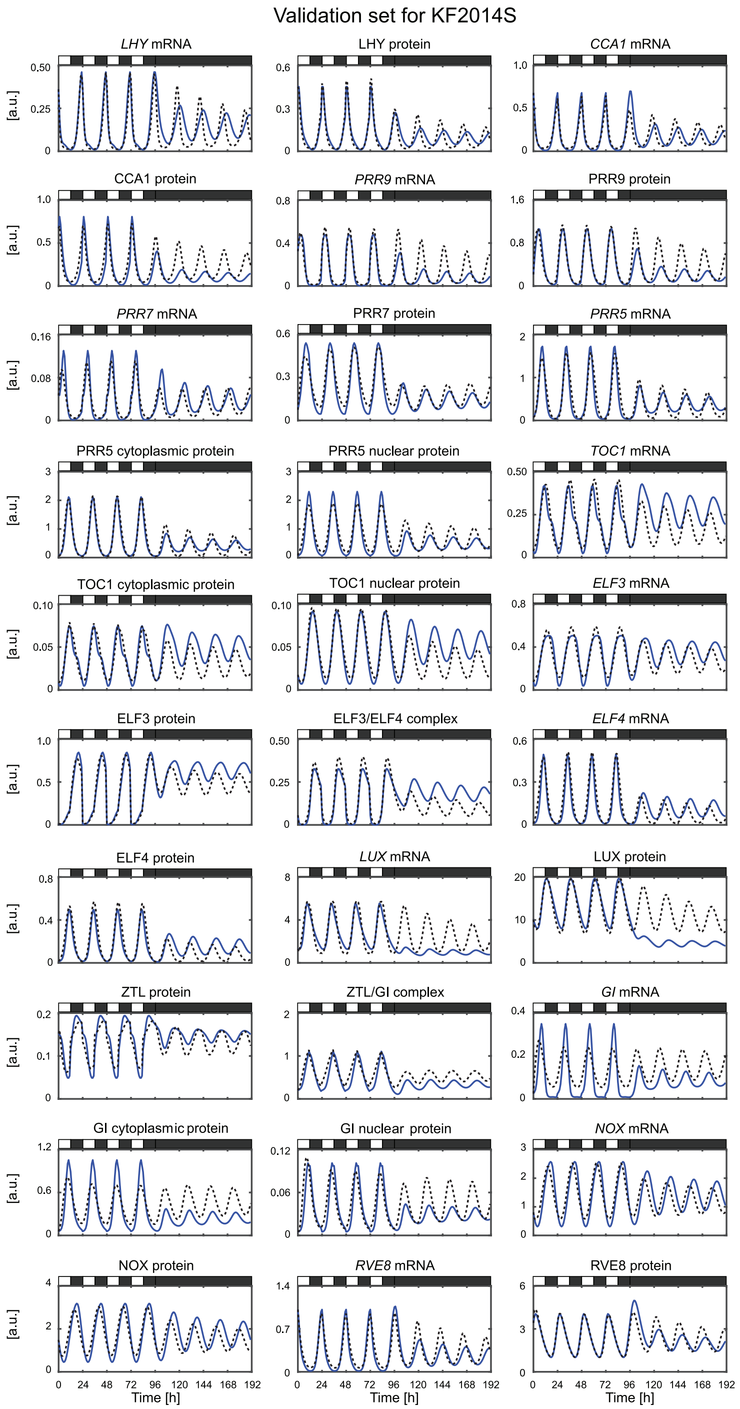

S6 Fig. Plant clock model KF2014S – fits to synthetic validation data. Blue solid lines show timeseries generated by KF2014 from its nominal parameter values for a simulated transition between a 12L:12D light-dark cycle and constant dark (DD). Black dashed lines show timeseries generated by KF2014S in the same simulated light environment using the parameters optimised to the training data (see **Fig 4C** for the corresponding heatmaps). White and black bars at the top of the figure indicate light and dark intervals, respectively.

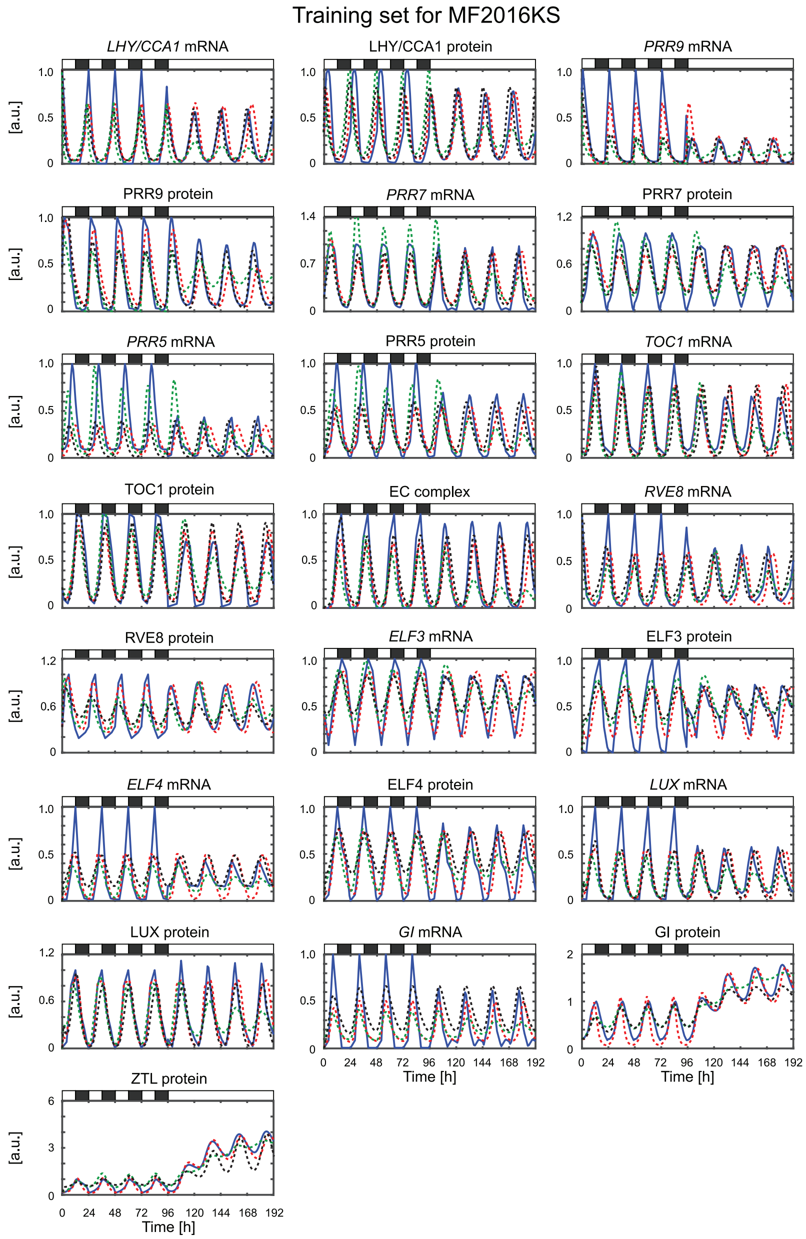

S7 Fig. Plant clock models MF2016KS, MF2016KSorig and MF2016K – optimal fits to experimental training data. Blue solid lines show timeseries recorded experimentally during a transition between a 12L:12D light-dark cycle and constant light (LL). Black (MF2016KS) and green (MF2016KSorig) dashed lines show timeseries obtained by optimising the parameters of the S-System models to this data in the same simulated light environment. Red dashed lines show optimal fits of MF2016K to the same data, obtained previously in [15] (see **Fig 5B** for the corresponding heatmaps). White and black bars at the top of the figure indicate light and dark intervals, respectively.

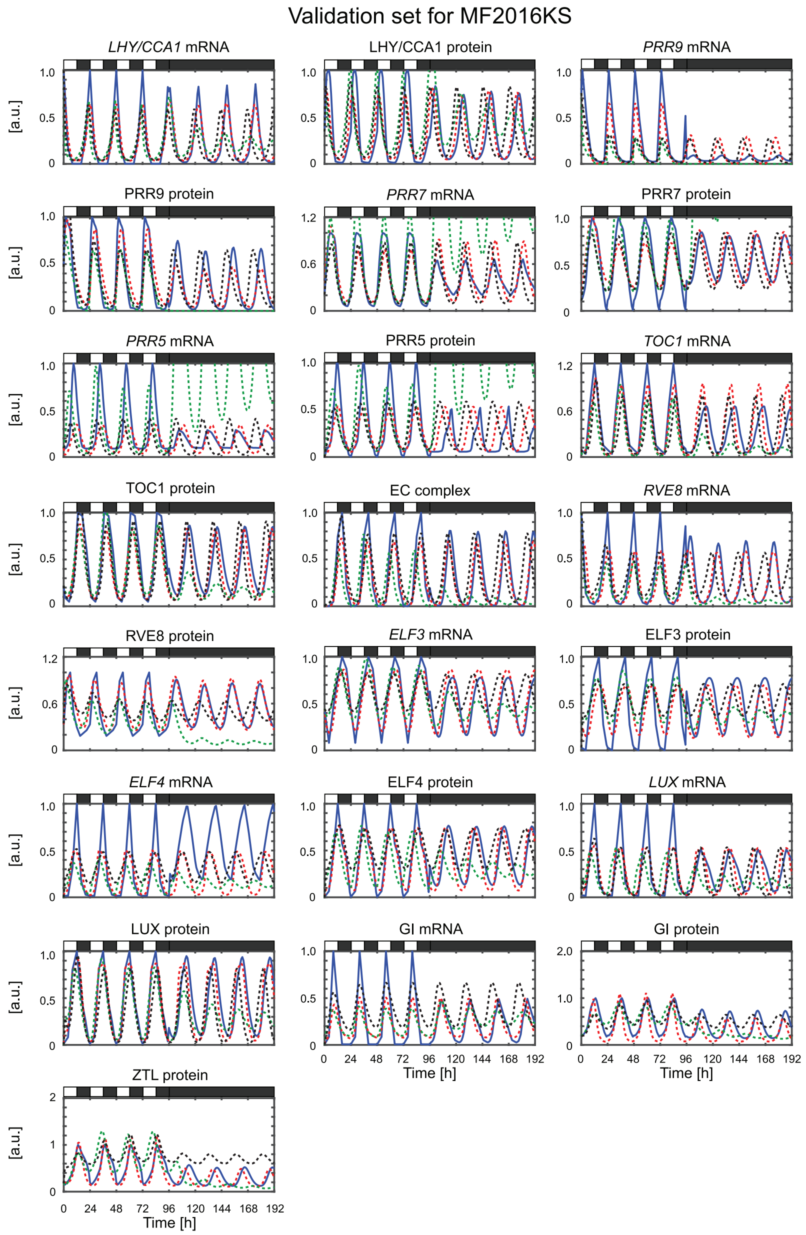

S8 Fig. Plant clock models MF2016KS, MF2016KSorig and MF2016K – fits to experimental validation data. Blue solid lines show timeseries recorded experimentally during a transition between a 12L:12D light-dark cycle and constant dark (DD). Black (MF2016KS) and green (MF2016KSorig) dashed lines show timeseries generated by the S-System models in the same simulated light environment using the parameters optimised to the training data. Red dashed lines show the corresponding fits of MF2016K to the same data (see **Fig 5C** for the corresponding heatmaps). White and black bars at the top of the figure indicate light and dark intervals, respectively.

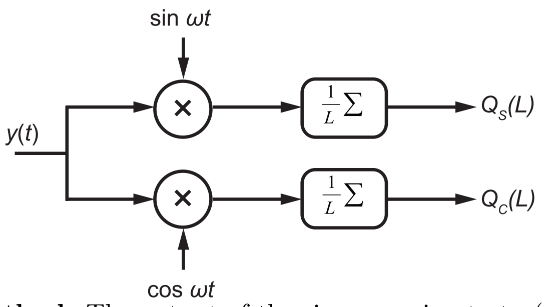

S9 Fig. Correlation method. The output of the sine sweeping test $y(t)$ is correlated with $\sin \omega t$ and $\cos \omega t$ prior to averaging to obtain the corresponding magnitude and phase values required to construct a Bode plot.

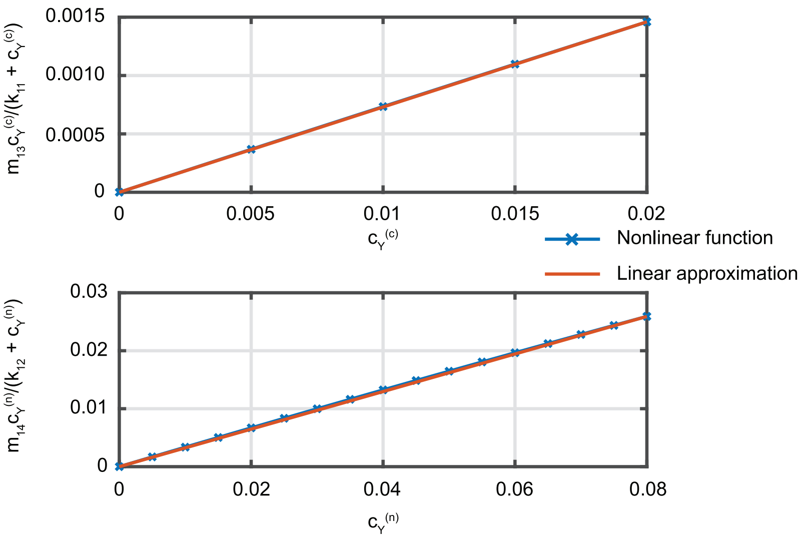

S10 Fig. Linear approximations to nonlinear Y protein degradation in JL2006. Blue lines show how the degradation rates of cytoplasmic Y protein (top panel) and nuclear Y protein (bottom panel) depend on the corresponding expression levels, $c_Y^{(c)}$ and $c_Y^{(n)}$, respectively. In each case, degradation rate is plotted for expression levels ranging between 0 and the maximum level observed in the synthetic training and validation datasets (see **S1 Fig** and **S2 Fig**). In these ranges, the nonlinear functions are well-approximated by linear fits (red lines), the gradients of which are taken as the values of γ_{cy} and γ_{nu} used to derive eq. (S3.5) in **S1 Text**.

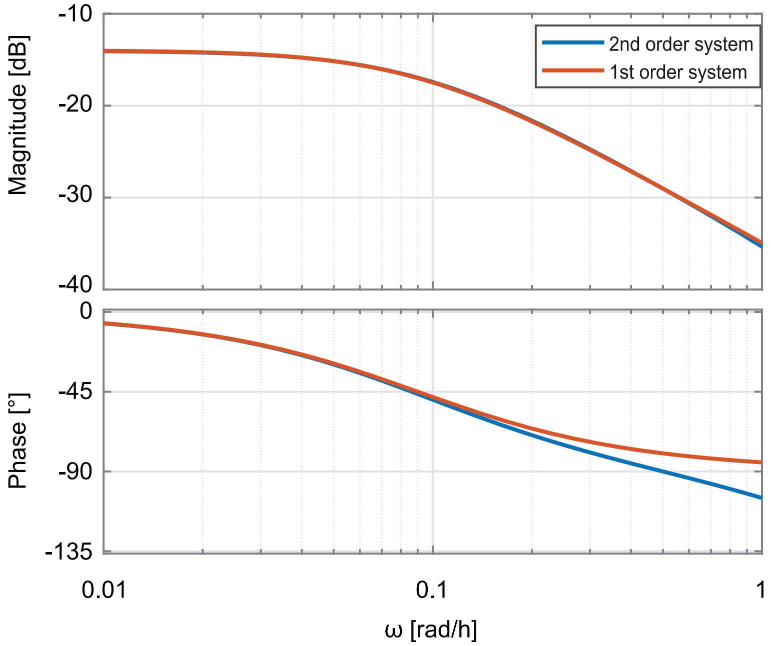

S11 Fig. Bode plot relating input *Y* mRNA to output Y nuclear protein in JL2006. Blue lines represent the second-order system given by eq. (S3.5) in **S1 Text**. Red lines represent the first-order system given by eq. (S3.7) that approximates eq. (S3.5).

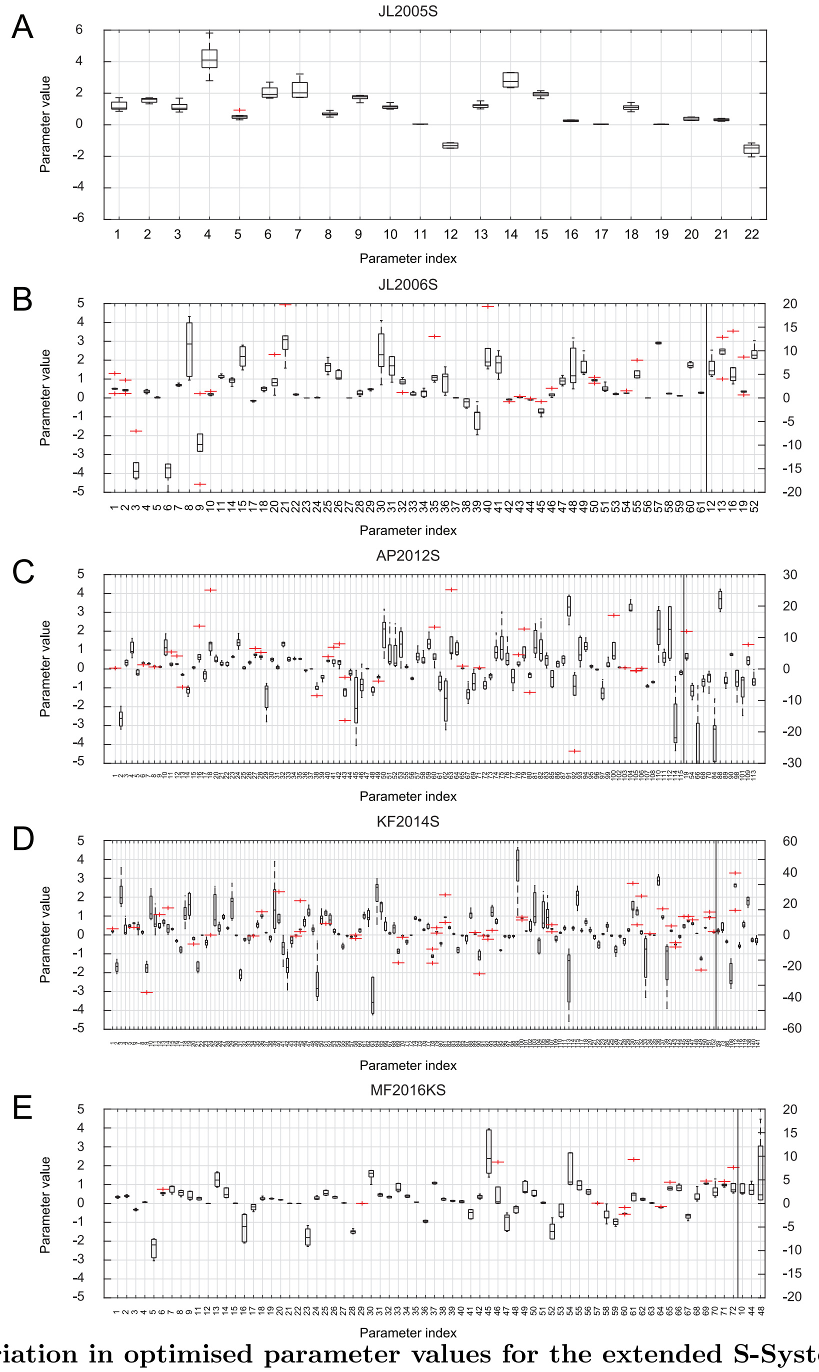

S12 Fig. Variation in optimised parameter values for the extended S-System models. **A-D:** Fits of JL2005S, JL2006S, AP2012S and KF2014S to synthetic data. **E:** Fits of MF2016KS to experimental data. Boxplots show parameter distributions obtained from six independent optimisation runs. In each boxplot, the horizontal line denotes the median value, the edges of the box are the 25th and 75th percentiles, the whiskers denote the most extreme datapoints not considered to be outliers, and outliers are plotted as red crosses. Model parameter indices are defined in **S3 Table** (JL2005S), **S6 Table** (JL2006S), **S9 Table** (AP2012S), **S12 Table** (KF2014S) and **S14 Table** (MF2016KS). In **B-E**, the thick black horizontal lines separate parameters whose values are plotted with respect to the left and right y-axes.

Supplementary Tables

i	Gene
L	*LHY/CCA1* or *LHY*
T	*TOC1*
X	*X*
Y	*Y/GI*
A	*PRR7/9*
$P9$	*PRR9*
$P7$	*PRR7*
$NI/P5$	*PRR5*
$Lmod$	Modified LHY/CCA1 protein
$Ltot$	Total amount of LHY/CCA1 protein
$E4$	*ELF4*
LUX	*LUX*
ZTL	*ZTL*
$E3$	*ELF3*
GI	*GI*
$E3c$	ELF3 cytoplasmic protein
Gc	GI cytoplasmic protein
$COP1c$	COP1 cytoplasmic protein
$E3n$	ELF3 nuclear protein
Gn	GI nuclear protein
$COP1d$	COP1 nuclear protein – day form
$COP1n$	COP1 nuclear protein – night form
EGc	ELF3-GI cytoplasmic protein complex
ZG	ZTL-GI cytoplasmic protein complex
EGn	ELF3-GI nuclear protein complex
$E34$	ELF3-ELF4 nuclear protein complex
EC	ELF3-ELF4-LUX nuclear protein complex
Gn_tot	Total amount of GI nuclear protein
$R/R8$	*RVE8*
C	*CCA1*
$E4d$	ELF4 dimer
NOX	*NOX*
$COP1$	COP1

S1 Table. Variables used in the equations for plant clock models JL2005 [11], JL2006 [43], AP2012 [44], KF2014 [45] and MF2016K [15].

Parameter	Value	Parameter	Value	Parameter	Value
a	1	b	2	n_1	16.9711
n_2	1.3043	g_1	3.0351	g_2	0.3860
m_1	9.3383	m_2	16.9058	m_3	1.0214
m_4	1.6859	m_5	0.4212	m_6	0.0484
m_7	1.2	k_1	1.3294	k_2	0.8085
k_3	0.1445	k_4	0.2089	k_5	0.3187
k_6	0.3505	k_7	1.2	p_1	4.9753
p_2	1.2947	p_3	0.5	r_1	1.4563
r_2	0.8421	r_3	0.0451	r_4	0.0018
q_1	2.9741	q_2	1	—	—

S2 Table. Nominal parameter values for JL2005, which were used to generate synthetic data. The parameters were taken from Figure 5 of [11].

Parameter	Value (nMAD)	Parameter	Value (nMAD)	Parameter	Value (nMAD)	
α_1	1.4361 (0.1320)	$g_{1,1}$	1.3276 (0.0439)	$\beta_{1,1}$	1.2875 (0.1499)	1–3
$\gamma_{1,1}$	4.7463 (0.1370)	α_2	0.4074 (0.1688)	$g_{2,1}$	2.0764 (0.1027)	4–6
$\beta_{2,1}$	1.7443 (0.1415)	α_3	0.9147 (0.0912)	$g_{3,1}$	1.7344 (0.0445)	7–9
$\beta_{3,1}$	1.4088 (0.0675)	α_4	0.0484 (0.1139)	$g_{4,1}$	−1.4874 (0.1198)	10–12
$\beta_{4,1}$	1.5163 (0.0687)	α_5	2.3917 (0.1373)	$g_{5,1}$	1.9657 (0.0519)	13–15
$\beta_{5,1}$	0.2075 (0.1665)	α_6	0.0378 (0.1310)	$g_{6,1}$	1.1309 (0.1009)	16–18
$\beta_{6,1}$	0.0268 (0.1353)	$\beta_{7,1}$	0.4886 (0.2460)	$\gamma_{7,1}$	0.4080 (0.1910)	19–21
$\gamma_{7,2}$	−1.5690 (0.1698)	—	—	—	—	22

S3 Table. Optimal parameter values $\hat{\Theta}^S_{LL}$ for the extended S-System formulation JL2005S of JL2005, obtained by fitting the model to the synthetic training data. For each parameter, the number in brackets is the normalised median absolute deviation (nMAD). This is calculated using the value shown, together with those obtained from five additional, independent optimisation runs. The rightmost column shows the parameter indexing, counting left to right across rows, that is used in **S12 Fig**.

Training dataset		Validation dataset	
Gene/protein	$W_i\left(X_i^{LL}, \hat{X}_i^{LL}\left(\hat{\Theta}_{LL}^S\right)\right)$ $(\times 10^{-3})$	Gene/protein	$W_i\left(X_i^{DD}, \hat{X}_i^{DD}\left(\hat{\Theta}_{LL}^S\right)\right)$ $(\times 10^{-3})$
LHY/CCA1	0.379	*LHY/CCA1*	0.388
LHY/CCA1 (cyt.)	0.393	LHY/CCA1 (cyt.)	0.405
LHY/CCA1 (nuc.)	1.005	LHY/CCA1 (nuc.)	0.998
TOC1	3.097	*TOC1*	2.915
TOC1 (cyt.)	4.461	TOC1 (cyt.)	4.530
TOC1 (nuc.)	0.179	TOC1 (nuc.)	0.196
Protein P	0.094	Protein P	0.407
$W\left(\mathbf{X}_{LL}, \hat{\mathbf{X}}_{LL}\left(\hat{\Theta}_{LL}^S\right)\right)$ $(\times 10^{-3})$	1.372	$W\left(\mathbf{X}_{DD}, \hat{\mathbf{X}}_{DD}\left(\hat{\Theta}_{LL}^S\right)\right)$ $(\times 10^{-3})$	1.406

S4 Table. The component-wise (W_i) and total (W) weighted mean squared error (WMSE) values obtained when fitting JL2005S to the synthetic training and validation datasets.

Parameter	Value	Parameter	Value	Parameter	Value
q_1	4.1954	n_0	0.0500	g_0	1.0000
α	4.0000	n_1	7.8142	a	1.2479
g_1	3.1383	m_1	1.9990	k_1	2.3920
p_1	0.8295	r_1	16.8363	r_2	0.1687
m_2	20.4400	k_2	1.5644	m_3	3.6888
k_3	1.2765	n_2	3.0087	b	1.0258
g_2	0.0368	g_3	0.2658	c	1.0258
m_4	3.8231	k_4	2.5734	p_2	4.3240
r_3	0.3166	r_4	2.1509	m_5	0.0013
m_6	3.1741	k_5	2.7454	m_7	0.0492
m_8	4.0424	k_6	0.4033	n_3	0.2431
d	1.4422	g_4	0.5388	m_9	10.1132
k_7	6.5585	p_3	2.1470	r_5	1.0352
r_6	3.3017	m_{10}	0.2179	k_8	0.6632
m_{11}	3.3442	k_9	17.1111	q_2	2.4017
n_4	0.0857	n_5	0.1649	g_5	1.1780
g_6	0.0645	e	3.6064	f	1.0237
m_{12}	4.2970	k_{10}	1.7303	p_4	0.2485
r_7	2.2123	r_8	0.2002	m_{13}	0.1347
k_{11}	1.8258	m_{14}	0.6114	k_{12}	1.8066
p_5	0.5000	k_{13}	1.2000	m_{15}	1.2000
q_3	1.0000	q_4	2.4514	g	1.0258
n_6	8.0706	g_7	0.0004	m_{16}	12.2398
k_{14}	10.3617	p_6	0.2907	r_9	0.2528
r_{10}	0.2212	m_{17}	4.4505	k_{15}	0.0703
m_{18}	0.0156	k_{16}	0.6104	—	—

S5 Table. Nominal parameter values for JL2006, which were used to generate synthetic data. The parameter values were taken from inline Supplementary Table 1 of [43].

Parameter	Value (nMAD)	Parameter	Value (nMAD)	Parameter	Value (nMAD)	
$\beta_{1,1}$	0.4597 (0.0503)	$\gamma_{1,1}$	0.3771 (0.0879)	$\gamma_{1,2}$	-4.2379 (0.0974)	1–3
α_2	0.4537 (0.1982)	$g_{2,1}$	0.0012 (0.7657)	$g_{2,2}$	-3.5046 (0.0566)	4–6
$\beta_{2,1}$	0.6117 (0.0567)	$\gamma_{2,1}$	3.0769 (0.4517)	$g_{2,3}$	-2.8305 (0.1872)	7–9
α_3	0.1151 (0.1971)	$g_{3,1}$	1.0993 (0.0323)	$\beta_{3,1}$	4.5548 (0.1768)	10–12
α_4	9.2696 (0.0549)	$g_{4,1}$	0.8282 (0.0967)	$\beta_{4,1}$	2.7978 (0.2342)	13–15
α_5	3.6836 (0.2521)	$g_{5,1}$	-0.2164 (0.0327)	$g_{5,2}$	0.5232 (0.1600)	16–18
$\beta_{5,1}$	1.2220 (0.0959)	α_6	2.3001 (0.2314)	$g_{6,1}$	1.5802 (0.1162)	19–21
$\beta_{6,1}$	0.2226 (0.1236)	$\gamma_{6,1}$	-0.0002 (0.2330)	α_7	0.0067 (0.6384)	22–24
$g_{7,1}$	1.8217 (0.1254)	$\beta_{7,1}$	1.0125 (0.0600)	$\gamma_{7,1}$	-0.0001 (0.7195)	25–27
α_8	0.1676 (0.4443)	$g_{8,1}$	0.4854 (0.0384)	$\beta_{8,1}$	1.7277 (0.3775)	28–30
α_9	2.1878 (0.2868)	$g_{9,1}$	1.0808 (0.1317)	$\beta_{9,1}$	0.1480 (0.1565)	31–33
α_{10}	0.3147 (0.3409)	$g_{10,1}$	1.1689 (0.1153)	$\beta_{10,1}$	1.1132 (0.2951)	34–36
α_{11}	0.0024 (0.6377)	$g_{11,1}$	-0.5448 (0.7978)	$g_{11,2}$	-1.6621 (0.3862)	37–39
$\beta_{11,1}$	1.8331 (0.1509)	$\gamma_{11,1}$	1.8447 (0.2400)	$g_{11,3}$	-0.0613 (0.2557)	40–42
$\gamma_{11,2}$	0.0150 (0.3048)	$g_{11,4}$	-0.1121 (0.0707)	$g_{11,5}$	-0.1966 (0.1436)	43–45
α_{12}	0.1160 (0.2427)	$g_{12,1}$	0.7780 (0.1837)	$\beta_{12,1}$	1.3022 (0.4499)	46–48
α_{13}	1.9436 (0.0615)	$g_{13,1}$	0.9501 (0.0406)	$\beta_{13,1}$	0.5854 (0.2023)	49–51
α_{14}	9.5387 (0.0763)	$g_{14,1}$	0.2558 (0.1048)	$\beta_{14,1}$	0.2512 (0.0113)	52–54
$\gamma_{14,1}$	1.0569 (0.0997)	α_{15}	0.0002 (0.0318)	$g_{15,1}$	2.9325 (0.0156)	55–57
$\beta_{15,1}$	0.2546 (0.0768)	α_{16}	0.1239 (0.0785)	$g_{16,1}$	1.6268 (0.0625)	58–60
$\beta_{16,1}$	0.2576 (0.0840)	—	—	—	—	61

S6 Table. Optimal parameter values $\hat{\Theta}^S_{LL}$ for the extended S-System formulation JL2006S of JL2006, obtained by fitting the model to the synthetic training data. For each parameter, the number in brackets is the normalised median absolute deviation (nMAD). This is calculated using the value shown, together with those obtained from five additional, independent optimisation runs. The rightmost column shows the parameter indexing, counting left to right across rows, that is used in **S12 Fig**.

Training dataset		Validation dataset	
Gene/protein	$W_i\left(X_i^{LL}, \hat{X}_i^{LL}\left(\hat{\Theta}_{LL}^S\right)\right)$ $(\times 10^{-2})$	Gene/protein	$W_i\left(X_i^{DD}, \hat{X}_i^{DD}\left(\hat{\Theta}_{LL}^S\right)\right)$ $(\times 10^{-2})$
Protein P	0.076	Protein P	0.152
LHY/CCA1	0.356	*LHY/CCA1*	0.547
LHY/CCA1 (cyt.)	0.175	LHY/CCA1 (cyt.)	0.250
LHY/CCA1 (nuc.)	0.399	LHY/CCA1 (nuc.)	0.536
TOC1	0.549	*TOC1*	1.236
TOC1 (cyt.)	0.478	TOC1 (cyt.)	1.464
TOC1 (nuc.)	0.476	TOC1 (nuc.)	1.809
X	1.094	*X*	1.551
X (cyt.)	0.165	X (cyt.)	0.678
X (nuc.)	0.185	X (nuc.)	0.458
Y	0.929	*Y*	4.227
Y (cyt.)	0.552	Y (cyt.)	3.411
Y (nuc.)	0.539	Y (nuc.)	3.811
PRR9/PRR7	1.440	*PRR9/PRR7*	1.566
PRR9/PRR7 (cyt.)	3.294	PRR9/PRR7 (cyt.)	3.233
PRR9/PRR7 (nuc.)	1.171	PRR9/PRR7 (nuc.)	1.000
$W\left(\mathbf{X}_{LL}, \hat{\mathbf{X}}_{LL}\left(\hat{\Theta}_{LL}^S\right)\right)$ $(\times 10^{-2})$	0.742	$W\left(\mathbf{X}_{DD}, \hat{\mathbf{X}}_{DD}\left(\hat{\Theta}_{LL}^S\right)\right)$ $(\times 10^{-2})$	1.621

S7 Table. The component-wise (W_i) and total (W) weighted mean squared error (WMSE) values obtained when fitting JL2006S to the synthetic training and validation datasets.

Parameter	Value	Parameter	Value	Parameter	Value
n_1	2.600	n_2	0.640	n_3	0.290
n_4	0.070	n_5	0.230	n_6	20.000
n_7	0.200	n_8	0.500	n_9	0.200
n_{10}	0.400	n_{11}	0.600	n_{12}	12.500
n_{13}	1.300	n_{14}	0.100	m_1	0.540
m_2	0.240	m_3	0.200	m_4	0.200
m_5	0.300	m_6	0.300	m_7	0.700
m_8	0.400	m_9	1.100	m_{10}	1.000
m_{11}	1.000	m_{12}	1.000	m_{13}	0.320
m_{14}	0.400	m_{15}	0.700	m_{16}	0.500
m_{17}	0.500	m_{18}	3.400	m_{19}	0.200
m_{20}	0.600	m_{21}	0.080	m_{22}	0.100
m_{23}	1.800	m_{24}	0.100	m_{25}	1.800
m_{26}	0.500	m_{27}	0.100	m_{28}	20.000
m_{29}	5.000	m_{30}	3.000	m_{31}	0.300
m_{32}	0.200	m_{33}	13.000	m_{34}	0.600
m_{35}	0.300	m_{36}	0.100	m_{37}	0.800
m_{38}	0.500	m_{39}	0.300	p_1	0.130
p_2	0.270	p_3	0.100	p_4	0.560
p_5	4.000	p_6	0.600	p_7	0.300
p_8	0.600	p_9	0.800	p_{10}	0.540
p_{11}	0.510	p_{12}	3.400	p_{13}	0.100
p_{14}	0.140	p_{15}	3.000	p_{16}	0.620
p_{17}	4.800	p_{18}	4.000	p_{19}	1.000
p_{20}	0.100	p_{21}	1.000	p_{22}	0.500
p_{23}	0.370	p_{24}	10.000	p_{25}	8.000
p_{26}	0.300	p_{27}	0.800	p_{28}	2.000
p_{29}	0.100	p_{30}	0.900	p_{31}	0.100
g_1	0.100	g_2	0.010	g_3	0.600
g_4	0.010	g_5	0.150	g_6	0.300
g_7	0.600	g_8	0.010	g_9	0.300
g_{10}	0.500	g_{11}	0.700	g_{12}	0.200
g_{13}	1.000	g_{14}	0.004	g_{15}	0.400
g_{16}	0.300	a	2.000	b	2.000
c	2.000	d	2.000	e	2.000
f	2.000	q_1	1.000	q_2	1.560
q_3	2.800	—	—	—	—

S8 Table. Nominal parameter values for AP2012, used to generate synthetic data. The parameter values were taken from Supplemental Table 1 of [44].

Parameter	Value (nMAD)	Parameter	Value (nMAD)	Parameter	Value (nMAD)	
α_1	0.0290 (0.1534)	$g_{1,1}$	−1.9737 (0.1415)	$\beta_{1,1}$	0.2061 (0.3579)	1–3
$\gamma_{1,1}$	0.5920 (0.2203)	$\gamma_{1,2}$	−0.2176 (0.2879)	α_2	0.2947 (0.0596)	4–6
$\beta_{2,1}$	0.2925 (0.0727)	$\gamma_{2,1}$	0.1456 (0.0350)	α_3	0.1305 (0.1813)	7–9
$g_{3,1}$	1.8637 (0.3554)	$\beta_{3,1}$	0.1863 (0.2494)	$\gamma_{4,1}$	0.2521 (0.0768)	10–12
$\gamma_{4,2}$	−0.2893 (0.0921)	$\gamma_{4,3}$	−0.9038 (0.1275)	α_5	0.0559 (0.5552)	13–15
$g_{5,1}$	0.5870 (0.2165)	$g_{5,2}$	−0.2868 (0.6610)	$\beta_{5,1}$	1.3060 (0.1714)	16–18
$\gamma_{5,1}$	3.3540 (0.1516)	α_6	0.5235 (0.1730)	$\beta_{6,1}$	0.2691 (0.2261)	19–21
$\gamma_{6,1}$	0.1829 (0.1442)	α_7	0.7157 (0.0326)	$g_{7,1}$	1.5493 (0.1140)	22–24
$g_{7,2}$	0.0207 (0.9377)	$\beta_{7,1}$	0.3529 (0.1056)	α_8	0.7861 (0.0512)	25–27
$\beta_{8,1}$	0.6875 (0.0688)	$\gamma_{8,1}$	−0.7648 (0.2123)	α_9	0.5541 (0.1388)	28–30
$g_{9,1}$	0.0147 (0.8411)	$g_{9,2}$	1.3107 (0.0387)	$\beta_{9,1}$	0.6289 (0.1578)	31–33
α_{10}	0.5433 (0.0438)	$\beta_{10,1}$	0.5071 (0.0437)	$\gamma_{10,1}$	−0.0970 (0.2574)	34–36
α_{11}	0.0002 (0.4581)	$g_{11,1}$	−1.4163 (0.0776)	$g_{11,2}$	−0.6852 (0.1574)	37–39
$\beta_{11,1}$	0.3701 (0.0827)	α_{12}	0.4783 (0.1988)	$\beta_{12,1}$	0.4027 (0.2668)	40–42
$\beta_{12,2}$	1.1809 (0.1740)	$\beta_{12,3}$	0.3230 (0.4737)	$\gamma_{12,1}$	−2.8598 (0.5763)	43–45
$\gamma_{12,2}$	−0.7289 (0.3715)	α_{13}	0.0038 (0.3732)	$g_{13,1}$	−0.9857 (0.0849)	46–48
$g_{13,2}$	−0.6447 (0.0934)	$\beta_{13,1}$	1.1210 (0.3186)	α_{14}	0.3801 (0.3350)	49–51
$\beta_{14,1}$	0.2772 (0.4896)	$\beta_{14,2}$	−0.9820 (0.5148)	$\beta_{14,3}$	9.7979 (0.2392)	52–54
α_{15}	0.0708 (0.3394)	$g_{15,1}$	−0.5501 (0.0778)	$\beta_{15,1}$	0.4745 (0.2802)	55–57
α_{16}	0.6022 (0.1699)	$g_{16,1}$	1.5735 (0.1845)	$\beta_{16,1}$	0.7994 (0.2639)	58–60
$\beta_{16,2}$	0.3805 (0.5153)	$\beta_{16,3}$	0.8501 (0.6288)	α_{17}	0.8086 (0.2224)	61–63
$g_{17,1}$	0.8162 (0.1737)	$\beta_{17,1}$	0.0319 (0.5023)	$\beta_{17,2}$	27.0370 (0.3123)	64–66
$\beta_{17,3}$	1.3398 (0.1968)	$\beta_{17,4}$	3.2021 (0.2828)	$\beta_{17,5}$	7.7305 (0.4394)	67–69
$\beta_{17,6}$	1.1152 (0.4048)	α_{18}	0.0029 (0.8184)	$g_{18,1}$	−0.8698 (0.1971)	70–72
$g_{18,2}$	−0.5739 (0.2616)	$\beta_{18,1}$	0.8866 (0.3467)	α_{19}	0.5398 (0.5227)	73–75
$\beta_{19,1}$	0.1984 (0.5155)	$\beta_{19,2}$	0.7364 (0.7702)	α_{20}	0.2307 (0.2429)	76–78
$\beta_{20,1}$	0.7061 (0.2673)	$\gamma_{20,1}$	−0.2873 (0.2455)	α_{21}	0.6099 (0.3554)	79–81
$g_{21,1}$	0.9981 (0.4707)	$\beta_{21,1}$	0.1988 (0.3901)	$\gamma_{21,1}$	−19.7551 (0.2803)	82–84
$\gamma_{21,2}$	−0.2852 (0.8160)	α_{22}	0.0999 (0.3563)	$\beta_{22,1}$	0.3003 (0.2649)	85–87
$\gamma_{22,1}$	19.1516 (0.1202)	$\gamma_{22,2}$	−4.6314 (0.2319)	α_{23}	5.0022 (0.0584)	88–90
$\beta_{23,1}$	3.9124 (0.1589)	$\beta_{23,2}$	1.2039 (0.5806)	$\beta_{23,3}$	−0.1068 (0.6249)	91–93
α_{24}	1.1125 (0.1593)	$\beta_{24,1}$	0.1419 (0.3489)	$\beta_{24,2}$	0.0059 (0.5425)	94–96
$\beta_{24,3}$	1.0111 (0.2217)	$\gamma_{24,1}$	−5.5232 (0.6169)	α_{25}	0.1391 (0.2364)	97–99
$\beta_{25,1}$	0.6014 (0.2564)	$\gamma_{25,1}$	−3.7469 (0.2515)	$\gamma_{25,2}$	0.0920 (0.1545)	100–102
$\beta_{26,1}$	0.0805 (0.0431)	$\gamma_{26,1}$	3.4317 (0.0135)	$\gamma_{26,2}$	−0.0918 (0.0176)	103–105
α_{27}	0.0130 (0.2434)	$g_{27,1}$	−0.8984 (0.0410)	$g_{27,2}$	−0.7319 (0.0300)	106–108
$\beta_{27,1}$	3.8473 (0.4487)	$\gamma_{27,1}$	2.1840 (0.4260)	α_{28}	0.4535 (0.4570)	109–111
$\beta_{28,1}$	1.3219 (0.5762)	$\beta_{28,2}$	4.3258 (0.2449)	$\gamma_{28,1}$	−3.8148 (0.1300)	112–114
$\gamma_{28,2}$	−0.1205 (0.4523)	—	—	—	—	115

S9 Table. Optimal parameter values $\hat{\Theta}^{S}_{LL}$ for the extended S-System formulation AP2012S of AP2012, obtained by fitting the model to the synthetic training data. For each parameter, the number in brackets is the normalised median absolute deviation (nMAD). This is calculated using the value shown together with those obtained from five additional, independent optimisation runs. The rightmost column shows the parameter indexing, counting left to right across rows, that is used in **S12 Fig**.

Training dataset		Validation dataset	
Gene/protein	$W_i\left(X_i^{LL}, \hat{X}_i^{LL}\left(\hat{\Theta}_{LL}^S\right)\right)$ $(\times 10^{-2})$	Gene/protein	$W_i\left(X_i^{DD}, \hat{X}_i^{DD}\left(\hat{\Theta}_{LL}^S\right)\right)$ $(\times 10^{-2})$
LHY/CCA1	0.315	*LHY/CCA1*	0.361
LHY/CCA1	0.288	LHY/CCA1	0.186
LHY/CCA1 (mod.)	0.244	LHY/CCA1 (mod.)	0.994
Protein P	0.324	Protein P	1.113
PRR9	0.142	*PRR9*	0.140
PRR9	0.190	PRR9	0.697
PRR7	0.441	*PRR7*	1.119
PRR7	0.361	PRR7	0.358
NI	0.428	*NI*	0.470
NI	0.388	NI	0.392
TOC1	1.560	*TOC1*	1.877
TOC1	1.739	TOC1	1.486
ELF3	0.800	*ELF3*	0.727
ELF3 (cyt.)	1.393	ELF3 (cyt.)	1.654
ELF3 (nuc.)	2.861	ELF3 (nuc.)	7.345
ELF4	2.323	*ELF4*	1.811
ELF4	2.673	ELF4	3.643
LUX	1.573	*LUX*	1.082
LUX	1.833	LUX	0.777
COP1 (cyt.)	0.004	COP1 (cyt.)	0.003
COP1 (nuc.)	0.031	COP1 (nuc.)	0.031
COP1 (dark)	0.031	COP1 (dark)	0.029
ELF3/GI cplx.	5.575	ELF3/GI cplx.	1.058
GI	4.003	*GI*	0.720
GI	3.899	GI	0.986
EC	4.005	EC	1.906
ZTL	0.970	ZTL	0.074
ZTL/GI cplx.	0.703	ZTL/GI cplx.	0.148
$W\left(\mathbf{X}_{LL}, \hat{\mathbf{X}}_{LL}\left(\hat{\Theta}_{LL}^S\right)\right)$ $(\times 10^{-2})$	1.396	$W\left(\mathbf{X}_{DD}, \hat{\mathbf{X}}_{DD}\left(\hat{\Theta}_{LL}^S\right)\right)$ $(\times 10^{-2})$	1.114

S10 Table. The component-wise (W_i) and total (W) weighted mean squared error (WMSE) values obtained when fitting AP2012S to the synthetic training and validation datasets.

Parameter	Value	Parameter	Value	Parameter	Value
a_3	1.02200	a_4	9.86600	a_5	5.35300
a_6	2.27800	a_7	5.91700	a_8	4.36500
r_1	3.74700	r_2	2.38400	r_3	4.74700
r_4	16.30000	r_5	0.10000	r_6	0.47280
r_7	35.94000	r_8	2.22500	r_9	0.58850
r_{10}	21.80000	r_{11}	2.08600	r_{12}	6.03300
r_{13}	1.05300	r_{14}	12.66000	r_{15}	6.74300
r_{16}	0.15190	r_{17}	5.19900	r_{18}	1.20500
r_{19}	16.24000	r_{20}	0.14650	r_{21}	5.12700
r_{22}	1.97100	r_{23}	7.10000	r_{24}	16.33000
r_{25}	1.02700	r_{26}	5.46600	r_{27}	6.86400
r_{28}	8.39200	r_{29}	0.14230	r_{30}	2.71400
r_{31}	0.01041	r_{32}	4.77500	r_{33}	0.90260
r_{34}	0.05704	r_{35}	0.02929	r_{36}	0.49000
r_{37}	0.55400	r_{38}	0.05062	r_{40}	1.05100
r_{41}	0.33410	f_1	0.40900	f_2	2.02100
f_3	0.03313	f_4	0.10000	f_5	0.38530
f_6	0.25250	t_5	1.10300	t_6	0.58910
t_7	0.23170	t_8	0.14720	t_9	0.85430
m_1	0.99600	m_3	0.58890	m_4	0.37610
m_5	2.30000	m_6	0.01330	m_7	0.64870
m_8	5.43700	m_9	0.12250	m_{10}	0.01001
m_{11}	0.67710	m_{12}	1.98800	m_{13}	0.37600
m_{14}	4.91600	m_{15}	0.09303	m_{16}	0.58280
m_{17}	0.04744	m_{18}	2.42600	m_{19}	0.20000
m_{20}	1.80000	m_{21}	0.10000	m_{22}	0.30120
m_{23}	0.17640	m_{24}	2.84800	m_{25}	0.41760
m_{26}	1.57000	m_{27}	0.10000	m_{28}	0.02757
m_{29}	0.01000	m_{30}	5.49100	m_{31}	0.30000
m_{32}	5.68100	m_{33}	13.00000	m_{34}	0.11150
m_{35}	0.91880	m_{36}	0.57110	m_{37}	0.93910
m_{38}	7.97500	m_{39}	0.21600	m_{42}	0.37590
m_{43}	0.52140	m_{44}	0.35770	m_{45}	0.79440
m_{46}	7.54100	m_{47}	0.12930	p_6	0.60000
p_7	0.30000	p_{10}	0.20000	p_{11}	1.78000
p_{12}	8.00000	p_{13}	0.70000	p_{14}	0.30000
p_{15}	3.00000	p_{16}	0.40240	p_{23}	1.46100
p_{25}	1.11100	p_{28}	2.13000	p_{29}	25.20000
n_5	0.23000	n_6	20.00000	n_{14}	0.10000
q_1	0.12170	q_3	0.28730	—	—

S11 Table. Nominal parameter values for KF2014, which were used to generate synthetic data. The parameter values were taken from Table 3 (Parameter Set 2)[†] and Table 4 in Supporting Information Text S1 of [45] ([†]We note that in the original paper, Table 3 incorrectly lists parameter a_3 as a_1 – this has been fixed in our version of the table).

Parameter	Value (nMAD)	Parameter	Value (nMAD)	Parameter	Value (nMAD)	
α_1	0.1654 (0.1957)	$g_{1,1}$	−2.0558 (0.1338)	$\beta_{1,1}$	1.7392 (0.1980)	1–3
$\gamma_{1,1}$	0.1676 (0.6742)	α_2	0.4928 (0.1231)	$\beta_{2,1}$	0.6442 (0.0464)	4–6
$\gamma_{2,1}$	0.5449 (0.3852)	α_3	0.1344 (0.2636)	$g_{3,1}$	−1.5602 (0.1221)	7–9
$\beta_{3,1}$	0.8565 (0.2542)	$\gamma_{3,1}$	0.5030 (0.5635)	α_4	1.0750 (0.1336)	10–12
$\beta_{4,1}$	0.8219 (0.1316)	$\gamma_{4,1}$	0.0154 (0.6314)	$\gamma_{5,1}$	0.2759 (0.1062)	13–15
$\gamma_{5,2}$	−0.2823 (0.1282)	$\gamma_{5,3}$	−0.9646 (0.1703)	α_6	1.0913 (0.3099)	16–18
$g_{6,1}$	1.4395 (0.3645)	$g_{6,2}$	−0.1418 (0.2311)	$g_{6,3}$	−1.7397 (0.1179)	19–21
$g_{6,4}$	−0.0061 (0.5979)	$g_{6,5}$	−0.5398 (0.3225)	$g_{6,6}$	−0.00001 (0.4918)	22–24
$\beta_{6,1}$	0.8770 (0.4030)	$\gamma_{6,1}$	0.2028 (0.4994)	α_7	1.0909 (0.0882)	25–27
$\beta_{7,1}$	0.4303 (0.0934)	α_8	1.8273 (0.3023)	$g_{8,1}$	−0.0023 (0.8377)	28–30
$g_{8,2}$	−2.2365 (0.1103)	$g_{8,3}$	−0.1821 (0.2115)	$g_{8,4}$	−0.4600 (0.8290)	31–33
$g_{8,5}$	−0.0001 (0.6791)	$\beta_{8,1}$	0.4885 (0.1981)	α_9	1.0623 (0.0505)	34–36
$\beta_{9,1}$	0.1153 (0.0720)	$\gamma_{9,1}$	−0.0656 (0.5536)	α_{10}	0.3345 (0.7844)	37–39
$g_{10,1}$	1.0947 (0.2179)	$g_{10,2}$	−1.6729 (0.4925)	$g_{10,3}$	−1.2459 (0.2178)	40–42
$g_{10,4}$	−0.2541 (0.6014)	$g_{10,5}$	−0.0003 (0.5860)	$\beta_{10,1}$	1.8135 (0.0677)	43–45
α_{11}	0.9639 (0.2297)	$g_{11,1}$	1.0768 (0.1269)	$\beta_{11,1}$	0.5273 (0.3062)	46–48
$\beta_{11,2}$	1.9788 (0.1866)	α_{12}	1.3849 (0.2359)	$g_{12,1}$	0.5969 (0.0957)	49–51
$\beta_{12,1}$	1.1299 (0.3091)	α_{13}	0.2168 (0.4787)	$g_{13,1}$	0.0117 (0.6778)	52–54
$g_{13,2}$	−0.7642 (0.1369)	$g_{13,3}$	−0.0185 (0.4494)	$g_{13,4}$	−0.0537 (0.2395)	55–57
$g_{13,5}$	−0.0022 (0.2190)	$\beta_{13,1}$	4.0754 (0.2352)	α_{14}	0.1291 (0.1484)	58–60
$g_{14,1}$	0.8877 (0.0995)	$\beta_{14,1}$	0.4435 (0.2642)	$\beta_{14,2}$	3.3327 (0.1745)	61–63
α_{15}	2.9835 (0.1200)	$g_{15,1}$	1.7478 (0.0478)	$\beta_{15,1}$	0.2474 (0.0724)	64–66
α_{16}	0.0363 (0.3095)	$g_{16,1}$	0.2587 (0.5641)	$g_{16,2}$	−1.4757 (0.1274)	67–70
$g_{16,3}$	−0.0040 (0.3401)	$g_{16,4}$	−0.5084 (0.2028)	$g_{16,5}$	−0.0160 (0.7169)	70–72
$\beta_{16,1}$	9.8530 (0.3626)	α_{17}	0.9830 (0.0501)	$\beta_{17,1}$	0.8374 (0.0550)	73–75
α_{18}	0.2914 (0.1387)	$\beta_{18,1}$	0.3482 (0.0736)	$\beta_{18,2}$	1.4952 (0.0584)	76–78
α_{19}	0.0954 (0.0761)	$g_{19,1}$	−0.5924 (0.0846)	$\beta_{19,1}$	0.6617 (0.0188)	79–81
α_{20}	0.3773 (0.1210)	$\beta_{20,1}$	0.0911 (0.4273)	$\beta_{20,2}$	0.9687 (0.1628)	82–84
$\beta_{20,3}$	0.0102 (0.3749)	$\beta_{20,4}$	6.4433 (0.1107)	$\beta_{20,5}$	0.0077 (0.7329)	85–87
α_{21}	1.4329 (0.1150)	$\beta_{21,1}$	0.1281 (0.3959)	$\beta_{21,2}$	0.8576 (0.2013)	88–90
$\beta_{21,3}$	0.0123 (0.3396)	$\beta_{21,4}$	0.0019 (0.8196)	α_{22}	0.9879 (0.2061)	91–93
$g_{22,1}$	0.8199 (0.1257)	$g_{22,2}$	−0.8558 (0.0255)	$g_{22,3}$	−0.0400 (0.3991)	94–96
$g_{22,4}$	−0.0288 (0.7219)	$g_{22,5}$	−0.0170 (0.5535)	$\beta_{22,1}$	2.8521 (0.1502)	97–99
α_{23}	0.7927 (0.0158)	$\beta_{23,1}$	0.1775 (0.0482)	α_{24}	0.1696 (0.3730)	100–102
$\beta_{24,1}$	0.5155 (0.4003)	$\gamma_{24,1}$	−0.2182 (0.3942)	α_{25}	0.5644 (0.4300)	103–105
$g_{25,1}$	1.0340 (0.5346)	$\beta_{25,1}$	0.1672 (0.0666)	$\gamma_{25,1}$	−22.6595 (0.1185)	106–108
$\gamma_{25,2}$	−0.2877 (0.3616)	α_{26}	0.1043 (0.2607)	$\beta_{26,1}$	0.2457 (0.1609)	109–111
$\gamma_{26,1}$	19.5485 (0.0298)	$\gamma_{26,2}$	−4.5860 (0.3383)	α_{27}	0.3114 (0.1370)	112–114
$\beta_{27,1}$	1.5723 (0.1644)	$\beta_{27,2}$	8.0358 (0.0948)	$\gamma_{27,1}$	0.2021 (0.1401)	115–117
$\gamma_{27,2}$	0.6797 (0.1096)	α_{28}	7.5757 (0.2145)	$\beta_{28,1}$	0.1076 (0.2506)	118–120
$\gamma_{28,1}$	−0.2029 (0.7440)	$\gamma_{28,2}$	−0.7164 (0.2955)	α_{29}	0.0137 (0.2914)	121–123
$g_{29,1}$	0.4247 (0.1609)	$g_{29,2}$	−0.0043 (0.9354)	$g_{29,3}$	−0.6214 (0.2028)	124–126
$g_{29,4}$	−0.0041 (0.7542)	$g_{29,5}$	−0.5587 (0.3950)	$\beta_{29,1}$	0.2377 (0.2540)	127–129
α_{30}	1.7779 (0.1694)	$g_{30,1}$	1.4744 (0.1000)	$\beta_{30,1}$	0.2170 (0.4426)	130–132
$\beta_{30,2}$	0.1981 (0.8271)	$\gamma_{30,1}$	0.0026 (0.7952)	$\gamma_{30,2}$	−0.0002 (0.3149)	133–135
α_{31}	3.1990 (0.0760)	$g_{31,1}$	1.3817 (0.0602)	$\beta_{31,1}$	21.7247 (0.1037)	136–138
$\beta_{31,2}$	0.5155 (0.3443)	$\beta_{31,3}$	4.3588 (0.2647)	$\beta_{31,4}$	5.2823 (0.3421)	139–141
α_{32}	0.1731 (0.1577)	$g_{32,1}$	−0.6398 (0.0357)	$g_{32,2}$	−0.0134 (0.7423)	142–144
$\beta_{32,1}$	0.3799 (0.1749)	α_{33}	0.9841 (0.0441)	$\beta_{33,1}$	0.7988 (0.0527)	145–147
α_{34}	0.0913 (0.0449)	$g_{34,1}$	−1.8614 (0.0427)	$\beta_{34,1}$	0.3388 (0.0299)	148–150
α_{35}	1.2125 (0.0096)	$\beta_{35,1}$	0.1753 (0.0284)	—	—	151–152

S12 Table. Optimal parameter values $\hat{\Theta}^S_{LL}$ for the extended S-System formulation KF2014S of KF2014, obtained by fitting the model to the synthetic training data. For each parameter, the number in brackets is the normalised median absolute deviation (nMAD). This is calculated using the value shown, together with those obtained from five additional, independent optimisation runs. The rightmost column shows the parameter indexing, counting left to right across rows, that is used in **S12 Fig**.

Training dataset		Validation dataset	
Gene/protein	$W_i\left(X_i^{LL}, \hat{X}_i^{LL}\left(\hat{\Theta}_{LL}^S\right)\right)$ ($\times 10^{-2}$)	Gene/protein	$W_i\left(X_i^{DD}, \hat{X}_i^{DD}\left(\hat{\Theta}_{LL}^S\right)\right)$ ($\times 10^{-2}$)
LHY	0.272	*LHY*	2.173
LHY	0.342	LHY	0.944
CCA1	0.315	*CCA1*	1.386
LHY	0.876	LHY	2.791
Protein P	0.141	Protein P	0.139
PRR9	0.426	*PRR9*	3.551
PRR9	0.260	PRR9	3.126
PRR7	0.851	*PRR7*	1.449
PRR7	0.790	PRR7	0.882
PRR5	0.899	*PRR5*	0.746
PRR5 (cyt.)	0.389	PRR5 (cyt.)	0.457
PRR5 (nuc.)	0.665	PRR5 (nuc.)	1.105
TOC1	1.311	*TOC1*	4.574
TOC1 (cyt.)	0.848	TOC1 (cyt.)	3.975
TOC1 (nuc.)	0.734	TOC1 (nuc.)	3.126
ELF3	0.957	*ELF3*	2.538
ELF3	0.446	ELF3	1.693
ELF3/4 cplx.	0.580	ELF3/4 cplx.	4.329
ELF4	0.797	*ELF4*	1.445
ELF4	1.315	ELF4	2.222
ELF4 (dark)	1.008	ELF4 (dark)	0.723
LUX	0.752	*LUX*	5.856
LUX	0.340	LUX	6.002
COP1 (cyt.)	0.013	COP1 (cyt.)	0.011
COP1 (nuc.)	0.051	COP1 (nuc.)	0.369
COP1 (dark)	0.098	COP1 (dark)	0.052
ZTL	0.678	ZTL	0.899
ZTL/GI cplx.	1.240	ZTL/GI cplx.	2.750
GI	2.841	*GI*	4.867
GI (cyt.)	1.565	GI (cyt.)	3.816
GI (nuc.)	0.014	GI (nuc.)	0.033
NOX	1.344	*NOX*	2.326
NOX	1.222	NOX	2.223
RVE8	0.525	*RVE8*	1.302
RVE8	0.102	RVE8	0.925
$W\left(\mathbf{X}_{LL}, \hat{\mathbf{X}}_{LL}\left(\hat{\Theta}_{LL}^S\right)\right)$ ($\times 10^{-2}$)	0.757	$W\left(\mathbf{X}_{DD}, \hat{\mathbf{X}}_{DD}\left(\hat{\Theta}_{LL}^S\right)\right)$ ($\times 10^{-2}$)	2.240

S13 Table. The component-wise (W_i) and total (W) weighted mean squared error (WMSE) values obtained when fitting KF2014 to the synthetic training and validation datasets.

Parameter	Value (nMAD)	Parameter	Value (nMAD)	Parameter	Value (nMAD)	
α_1	0.2655 (0.0641)	$\beta_{1,1}$	0.3093 (0.0621)	$\gamma_{1,1}$	−0.2585 (0.0588)	1–3
α_2	0.0448 (0.2218)	$g_{2,1}$	−2.3152 (0.1407)	$\beta_{2,1}$	0.4314 (0.0594)	4–6
α_3	0.9112 (0.0275)	$\beta_{3,1}$	0.5660 (0.1865)	α_4	0.1780 (0.3857)	7–9
$g_{4,1}$	2.4323 (0.1074)	$\beta_{4,1}$	0.2501 (0.1580)	$\gamma_{4,1}$	0.00003 (0.8280)	10–12
α_5	1.5339 (0.2891)	$\beta_{5,1}$	0.5711 (0.3323)	α_6	0.0126 (0.4956)	13–15
$g_{6,1}$	−1.0164 (0.5353)	$g_{6,2}$	−0.2005 (0.7194)	$\beta_{6,1}$	0.3002 (0.1409)	16–18
α_7	0.2680 (0.0482)	$\beta_{7,1}$	0.2047 (0.0541)	α_8	0.0056 (0.3559)	19–21
$g_{8,1}$	−0.0002 (0.8012)	$g_{8,2}$	−1.3506 (0.2359)	$\beta_{8,1}$	0.4097 (0.1490)	22–24
α_9	0.5424 (0.1770)	$\beta_{9,1}$	0.2865 (0.0400)	α_{10}	0.0158 (0.4127)	25–27
$g_{10,1}$	−1.4978 (0.0413)	$g_{10,2}$	−0.0036 (0.0999)	$\beta_{10,1}$	1.0041 (0.0927)	28–30
α_{11}	0.5352 (0.1223)	$\beta_{11,1}$	0.3693 (0.0769)	α_{12}	0.7582 (0.1500)	31–33
$\beta_{12,1}$	0.3167 (0.1296)	α_{13}	0.0709 (0.0658)	$g_{13,1}$	−0.8926 (0.0513)	34–36
$\beta_{13,1}$	1.0452 (0.0182)	α_{14}	0.1568 (0.1125)	$\beta_{14,1}$	0.0930 (0.1129)	37–39
α_{15}	0.1438 (0.5179)	$g_{15,1}$	−0.3292 (0.3256)	$\beta_{15,1}$	0.3937 (0.2149)	40–42
α_{16}	3.1255 (0.3715)	$\beta_{16,1}$	3.5977 (0.3149)	α_{17}	2.1919 (0.9295)	43–45
$g_{17,1}$	−0.5002 (0.2369)	$g_{17,2}$	−0.1369 (0.3780)	$\beta_{17,1}$	17.8622 (0.5947)	46–48
α_{18}	0.6246 (0.1028)	$\beta_{18,1}$	0.3979 (0.1046)	α_{19}	0.0033 (0.8938)	49–51
$g_{19,1}$	−1.7339 (0.2632)	$g_{19,2}$	−0.7559 (0.5820)	$\beta_{19,1}$	1.0217 (0.1151)	52–54
α_{20}	1.1968 (0.2465)	$\beta_{20,1}$	0.7312 (0.1924)	α_{21}	0.0025 (0.3278)	55–57
$g_{21,1}$	−0.4076 (0.2263)	$g_{21,2}$	−1.2183 (0.1368)	$g_{21,3}$	−0.5346 (0.0086)	58–60
$\beta_{21,1}$	0.1365 (0.4582)	α_{22}	0.1667 (0.2345)	$\beta_{22,1}$	0.0462 (0.0550)	61–63
$\beta_{22,2}$	0.1660 (0.2186)	α_{23}	0.8759 (0.1023)	$\beta_{23,1}$	0.9016 (0.1738)	64–66
$\gamma_{23,1}$	−0.7762 (0.1125)	α_{24}	0.8941 (0.2909)	$\beta_{24,1}$	1.9106 (0.1944)	67–69
$\beta_{24,2}$	-1.2984 (0.0233)	$h_{24,1}$	1.0077 (0.3438)	$h_{24,2}$	1.0119 (0.0451)	70–72

S14 Table. Optimal parameter values $\hat{\Theta}^{E}_{LL}$ for the extended S-System formulation MF2016KS of MF2016K, obtained by fitting to the experimental training data. For each parameter, the number in brackets is the normalised median absolute deviation (nMAD). This is calculated using the value shown, together with the values obtained from five additional, independent optimisation runs. The rightmost column shows the parameter indexing, counting left to right across rows, that is used in **S12 Fig**.

Training dataset		Validation dataset	
Gene/protein	$W_i\left(D_i^{LL},\hat{X}_i^{LL}\left(\hat{\Theta}_{LL}^{E}\right)\right)$ $(\times 10^{-2})$	Gene/protein	$W_i\left(D_i^{DD},\hat{X}_i^{DD}\left(\hat{\Theta}_{DD}^{E}\right)\right)$ $(\times 10^{-2})$
Protein P	1.129	Protein P	1.398
LHY/CCA1	1.556	*LHY/CCA1*	7.479
LHY/CCA1	5.938	LHY/CCA1	15.590
PRR9	6.483	*PRR9*	75.888
PRR9	2.210	PRR9	10.053
PRR7	1.985	*PRR7*	7.371
PRR7	2.185	PRR7	5.475
PRR5	7.040	*PRR5*	23.256
PRR5	5.366	PRR5	20.200
TOC1	6.544	*TOC1*	16.076
TOC1	2.160	TOC1	7.310
EC	1.973	EC	7.654
RVE8	1.809	*RVE8*	9.331
RVE8	3.342	RVE8	5.313
ELF3	2.155	*ELF3*	8.870
ELF3	4.080	ELF3	8.904
ELF4	4.688	*ELF4*	9.684
ELF4	5.371	ELF4	7.174
LUX	3.084	*LUX*	10.656
LUX	1.821	LUX	7.245
GI	7.632	*GI*	18.080
GI	2.111	GI	5.779
COP1	0.703	COP1	0.444
ZTL	6.149	ZTL	36.542
$W\left(\mathbf{D}_{LL},\hat{\mathbf{X}}_{LL}\left(\hat{\Theta}_{LL}^{E}\right)\right)$ $(\times 10^{-2})$	3.647	$W\left(\mathbf{D}_{DD},\hat{\mathbf{X}}_{DD}\left(\hat{\Theta}_{LL}^{E}\right)\right)$ $(\times 10^{-2})$	13.557

S15 Table. The component-wise (W_i) and total (W) weighted mean squared error (WMSE) values obtained when fitting MF2016KS to the experimental training and validation datasets.

Parameter	Value	Parameter	Value	Parameter	Value
α_1	0.1233	$\beta_{1,1}$	0.8403	$h_{1,1}$	0.4190
$h_{1,2}$	0.6895	α_2	0.0588	$g_{2,1}$	−0.2158
$g_{2,2}$	−0.2705	$g_{2,3}$	−0.4991	$\beta_{2,1}$	1.9153
α_3	0.9834	$\beta_{3,1}$	0.4690	α_4	0.6552
$g_{4,1}$	0.7346	$g_{4,2}$	0.0309	$g_{4,3}$	1.3117
$\beta_{4,1}$	2.0981	α_5	1.3954	$\beta_{5,1}$	0.4393
α_6	0.0036	$g_{6,1}$	−2.2477	$g_{6,2}$	−0.0557
$\beta_{6,1}$	0.2495	α_7	0.2332	$\beta_{7,1}$	0.2131
α_8	0.0007	$g_{8,1}$	−0.00002	$g_{8,2}$	−2.9009
$\beta_{8,1}$	0.2543	α_9	0.5167	$\beta_{9,1}$	0.4078
α_{10}	0.0341	$g_{10,1}$	−1.6459	$g_{10,2}$	−0.0022
$\beta_{10,1}$	1.5880	α_{11}	0.3802	$\beta_{11,1}$	0.2508
α_{12}	0.9153	$\beta_{12,1}$	0.5608	α_{13}	0.0560
$g_{13,1}$	−0.8123	$\beta_{13,1}$	1.0326	α_{14}	0.3423
$\beta_{14,1}$	0.1800	α_{15}	0.0588	$g_{15,1}$	−0.7041
$\beta_{15,1}$	0.2594	α_{16}	1.8694	$\beta_{16,1}$	2.1363
α_{17}	0.0076	$g_{17,1}$	−1.5118	$g_{17,2}$	−0.4948
$\beta_{17,1}$	0.8314	α_{18}	1.2916	$\beta_{18,1}$	0.6880
α_{19}	0.0016	$g_{19,1}$	−2.1824	$g_{19,2}$	−0.8092
$\beta_{19,1}$	0.8623	α_{20}	1.1027	$\beta_{20,1}$	0.5859
α_{21}	0.0192	$g_{21,1}$	−0.7403	$g_{21,2}$	−0.5839
$g_{21,3}$	−0.4361	$\beta_{21,1}$	0.6222	α_{22}	0.1484
$\beta_{22,1}$	0.4972	$h_{22,1}$	2.1771	$h_{22,2}$	1.8459
$h_{22,3}$	1.3754	α_{23}	0.1013	$\beta_{23,1}$	1.2943
$h_{23,1}$	1.5038	$h_{23,2}$	1.3948	α_{24}	1.9545
$g_{24,1}$	0.0127	$g_{24,2}$	1.6218	$\beta_{24,1}$	1.2789

S16 Table. Optimal parameter values $\hat{\Theta}^{E}_{LL}$ for the original S-System formulation MF2016KSorig of MF2016K, obtained by fitting to the experimental training data.

Parameter	Value	Parameter	Value	Parameter	Value
ϕ_1	-0.4330	ϕ_2	-0.4949	ϕ_3	0.4358
ϕ_4	1.9601	ϕ_5	0.7412	ϕ_6	0.3821
ϕ_7	0.5264	ϕ_8	1.3764	ϕ_9	11.6684
ϕ_{10}	-2.7804	ϕ_{11}	0.8360	ϕ_{12}	-0.6472
ϕ_{13}	0.7337	ϕ_{14}	0.00006	ϕ_{15}	0.7598
ϕ_{16}	1.3352	ϕ_{17}	-0.5971	ϕ_{18}	0.5008
ϕ_{19}	-0.2234	ϕ_{20}	0.0099	ϕ_{21}	0.0347
ϕ_{22}	0.8773	ϕ_{23}	-0.2393	ϕ_{24}	0.2374
ϕ_{25}	-0.1857	ϕ_{26}	0.0267	ϕ_{27}	0.1320
ϕ_{28}	0.03159	ϕ_{29}	-40.9795	ϕ_{30}	4.0753
ϕ_{31}	-2.5872	ϕ_{32}	0.0150	ϕ_{33}	0.0369
ϕ_{34}	0.0113	ϕ_{35}	-12.2192	ϕ_{36}	0.4005
ϕ_{37}	-0.3117	ϕ_{38}	0.8082	ϕ_{39}	-0.3785
ϕ_{40}	2.2417	ϕ_{41}	1.4074	ϕ_{42}	-1.5159
ϕ_{43}	0.3285	ϕ_{44}	-0.1488	ϕ_{45}	0.0146
ϕ_{46}	0.2339	ϕ_{47}	-0.2726	ϕ_{48}	14.0123
ϕ_{49}	-17.3569	ϕ_{50}	0.0144	ϕ_{51}	0.0321
ϕ_{52}	0.0517	ϕ_{53}	-11.0070	ϕ_{54}	0.6736
ϕ_{55}	-0.3972	ϕ_{56}	0.0289	ϕ_{57}	0.0173
ϕ_{58}	0.0341	ϕ_{59}	-26.4166	ϕ_{60}	2.4610
ϕ_{61}	-1.4794	ϕ_{62}	0.0002	ϕ_{63}	0.0724
ϕ_{64}	0.1181	ϕ_{65}	0.4560	ϕ_{66}	-0.5845
ϕ_{67}	0.4025	ϕ_{68}	-0.7857	ϕ_{69}	-0.0208
ϕ_{70}	-0.8094	ϕ_{71}	-0.8102	ϕ_{72}	0.9142
ϕ_{73}	2.3354	ϕ_{74}	-0.4454	ϕ_{75}	0.0279
θ_{144}	1.275	θ_{145}	1.5983	—	—

S17 Table. Optimal parameter values for MF2016K, which were obtained previously in [15] by fitting to the experimental training data used in this study with the same optimisation method. The values are reproduced from Tables S2 and S4 in the Supporting Information of [15].
